# Supplementary material for: An omic and multidimensional spatial atlas from serial biopsies of an evolving metastatic breast cancer
Source: Cell Rep Med. 2022 Feb 15;3(2):100525. doi: 10.1016/j.xcrm.2022.100525 (PMC8861971; doi:10.1016/j.xcrm.2022.100525)
Supplement: Document S1. Figures S1–S6 and supplemental references [file mmc1.pdf]

Cell Reports Medicine, Volume *3*

Supplemental information

An omic and multidimensional spatial atlas from serial biopsies of an evolving metastatic breast cancer

Brett E. Johnson, Allison L. Creason, Jayne M. Stommel, Jamie M. Keck, Swapnil Parmar, Courtney B. Betts, Aurora Blucher, Christopher Boniface, Elmar Bucher, Erik Burlingame, Todd Camp, Koei Chin, Jennifer Eng, Joseph Estabrook, Heidi S. Feiler, Michael B. Heskett, Zhi Hu, Annette Kolodzie, Ben L. Kong, Marilyne Labrie, Jinho Lee, Patrick Leyshock, Souraya Mitri, Janice Patterson, Jessica L. Riesterer, Shamilene Sivagnanam, Julia Somers, Damir Sudar, Guillaume Thibault, Benjamin R. Weeder, Christina Zheng, Xiaolin Nan, Reid F. Thompson, Laura M. Heiser, Paul T. Spellman, George Thomas, Emek Demir, Young Hwan Chang, Lisa M. Coussens, Alexander R. Guimaraes, Christopher Corless, Jeremy Goecks, Raymond Bergan, Zahi Mitri, Gordon B. Mills, and Joe W. Gray

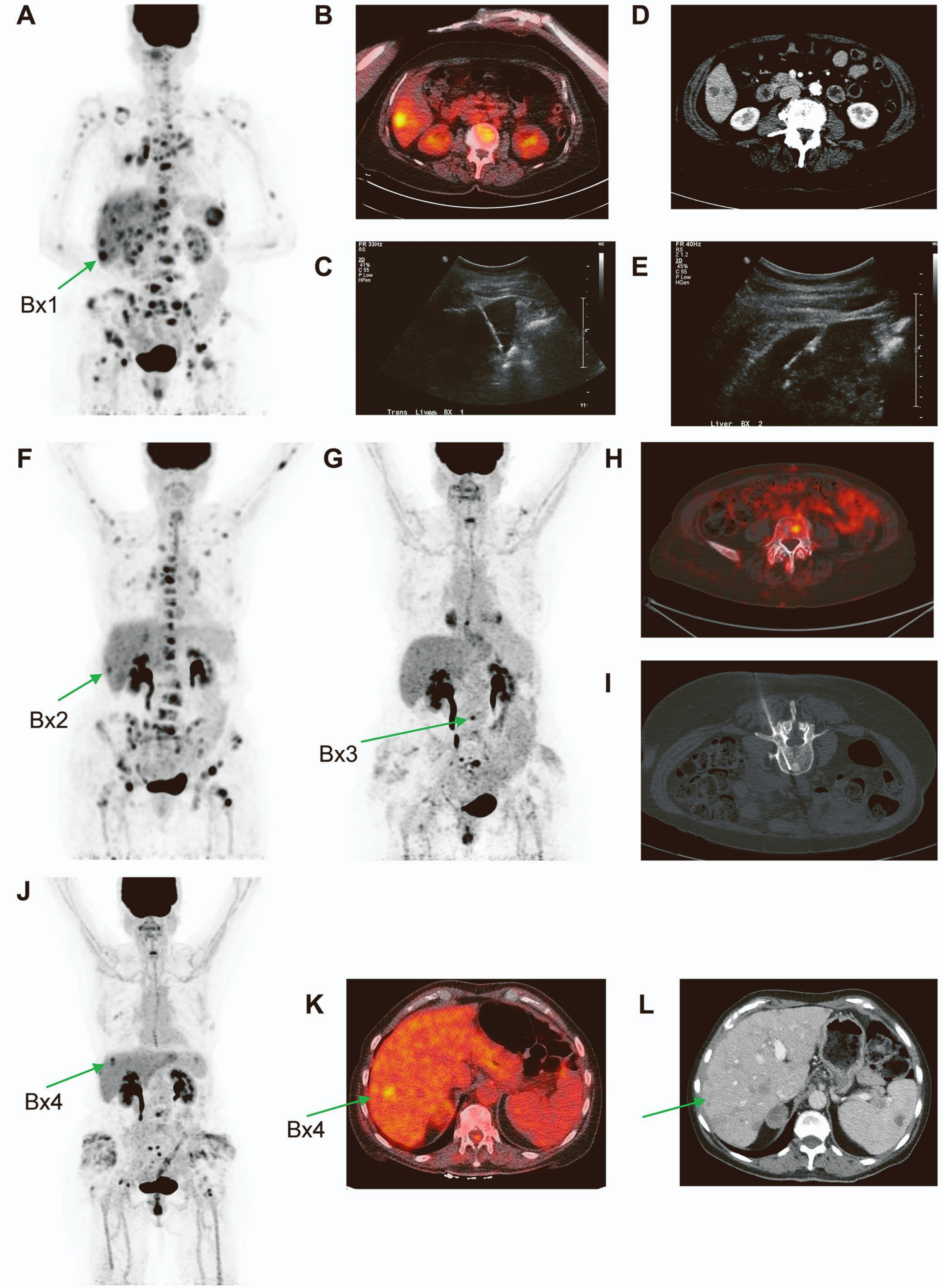

Figure S1. FDG-PET and CT Imaging, Related to Figure 2.
(A) Maximum intensity projection (MIP) FDG-PET from the beginning of Phase 1 treatment demonstrates multifocal FDG avid disease throughout the mediastinum, liver, spleen, and skeleton, including target of Bx1 (arrow). (B) Axial FDG-PET

(from same timepoint as in A) superimposed on CT showing FDG avid segment 6 liver lesion targeted by Bx1. (C) Ultrasound image from Bx1. (D) Axial contrast enhanced CT taken just before Bx2 demonstrates interval growth of a separate segment 5,6 liver lesion subsequently targeted by Bx2. (E) Ultrasound image from Bx2. (F) FDG-PET MIP image from the beginning of Phase 3 demonstrates a decrease in FDG avid lesions. Bx2 targeted lesion indicated (arrow). (G) FDG-PET taken in Phase 3 one month before Bx3 demonstrates new FDG avid lesions in the L4 vertebral body, including the Bx3 targeted lesion (arrow). (H) Axial FDG-PET (from same timepoint as in G) superimposed on attenuation correction CT showing FDG avid segment targeted by Bx3. (I) CT image from Bx3 demonstrates successful biopsy of the FDG avid, lytic lesion within the L4 vertebral body. Note the patient is prone during the biopsy. (J) FDG-PET from the end of Phase 3 demonstrates continued response in most organs but a possible new progressing liver lesion subsequently targeted by Bx4 (arrow). (K) Axial FDG-PET (from same timepoint as J) superimposed on CT showing FDG avid liver lesion targeted by Bx4 (arrow). (L) Axial contrast enhanced CT taken during month 37 demonstrates nodular, heterogeneous morphology of the liver showing signs of pseudocirrhosis (arrow).

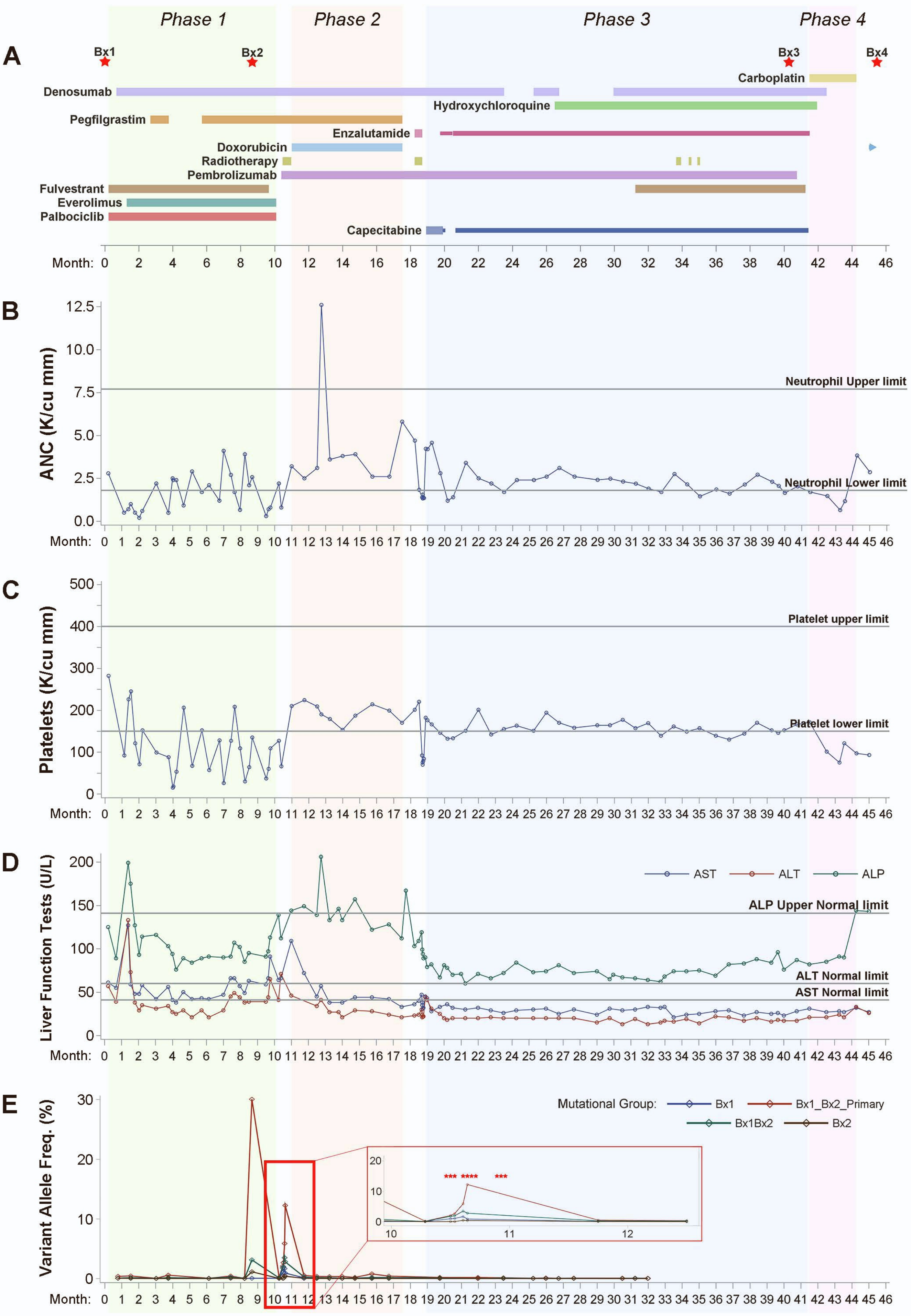
A
B
C
D
E
Phase 1
Phase 2
Phase 3
Phase 4
Bx1
Bx2
Bx3
Bx4
Carboplatin
Denosumab
Hydroxychloroquine
Pegfilgrastim
Enzalutamide
Doxorubicin
Radiotherapy
Pembrolizumab
Fulvestrant
Everolimus
Palbociclib
Capecitabine
Month:
ANC (K/cu mm)
Neutrophil Upper limit
Neutrophil Lower limit
Platelets (K/cu mm)
Platelet upper limit
Platelet lower limit
Liver Function Tests (U/L)
AST
ALT
ALP
ALP Upper Normal limit
ALT Normal limit
AST Normal limit
Variant Allele Freq. (%)
Mutational Group:
Bx1
Bx1_Bx2_Primary
Bx1Bx2
Bx2
***
****
***

Figure S2. Toxicity-Related Blood Chemistries and ctDNA Monitoring, Related to Figure 2.
(A) Treatment schedule and biopsy timing (red stars) over the course of four phases of treatment (green, orange, blue, and pink areas). Timeline sectioned into 28-day months. The duration and relative dose for each drug is indicated by the extent and width of a horizontal bar, respectively. Continuation of a drug after the end of Phase 4 is indicated by a right pointing arrow. (B) Clinically reported Absolute Neutrophil Count (ANC) in thousands per cubic millimeter (K/cu mm). (C) Clinically reported Platelet Count in thousands per cubic millimeter (K/cu mm). (D) Clinically reported results of liver function tests, including alkaline phosphatase (ALP), alanine aminotransferase (ALT), and aspartate aminotransferase (AST). (E) Longitudinal tracking of the average circulating tumor DNA (ctDNA) Variant Allele Frequency (VAF) of four different groups of mutations: Variants private to Bx1 (Bx1), private to Bx2 (Bx2), shared between the primary, Bx1, and Bx2 (Bx1_Bx2_Primary), and shared by Bx1 and Bx2 but not the primary (Bx1_Bx2). Red boxed inset shows expanded ctDNA VAF timeline during a course of palliative radiotherapy during month 10. Red asterisks indicate dates of individual radiation fractions given.

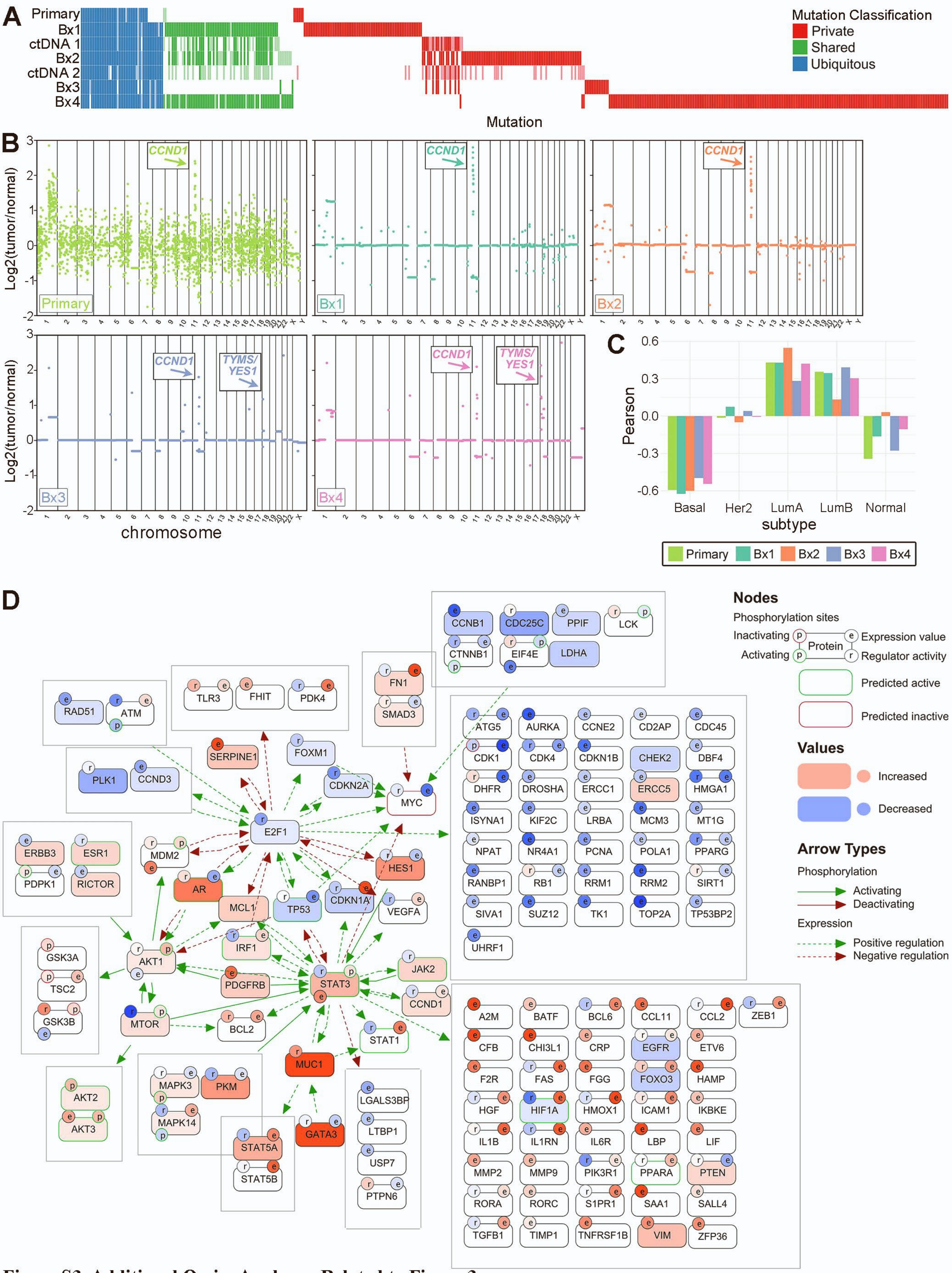

Figure S3. Additional Omics Analyses, Related to Figure 3.

(A) Non-silent SNVs and Indels identified from WES of tissue samples and classified as Ubiquitous (blue), Shared (green), or Private (red) (variants private to ctDNA timepoints not shown). Mutational status in each biopsy sample or in circulating tumor DNA from peripheral blood is indicated as independently called (colored), detected in at least 2 sequencing reads but not independently called (reduced opacity), or absent (white). (B) Scatter plots of genome-wide, segmented,

log2 copy number profiles from WES (Primary, Bx1, and Bx2) and LP-WGS (Bx3 and Bx4): Primary (yellow-green), Bx1 (green), Bx2 (orange), Bx3 (blue), and Bx4 (pink). (C) Molecular Subtype. Bar plots show the Pearson correlation of the Primary (yellow-green), Bx1 (green), Bx2 (orange), Bx3 (blue), and Bx4 (pink) samples to the PAM50 subtype centroids. (D) Integrated multi-omic pathway analysis. Pathway diagrams generated with CausalPath represent the integration of protein abundance (rectangles), phosphoprotein abundance (circle labeled ‘p’; green outline indicates activating; red outline indicates inactivating), gene expression (circle labeled ‘e’), and transcriptional regulator activity (circle labeled ‘r’), and show the change in Bx2 relative to Bx1. Networks were generated using protein/phosphoprotein abundance and gene expression, while transcriptional regulator activity was mapped on following network pruning. The red and blue fill represent higher and lower expression/activity, respectively.

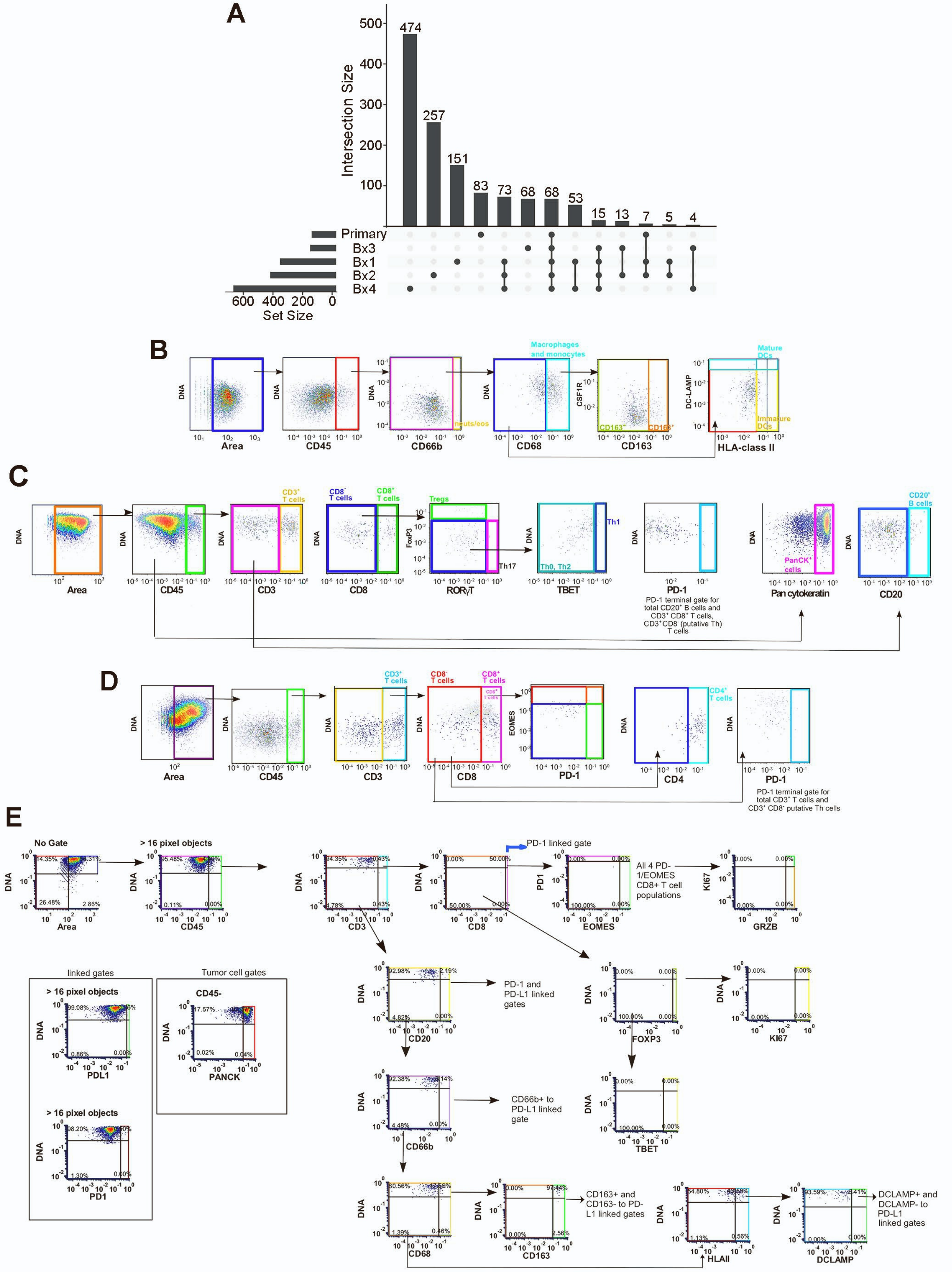

Figure S4. Predicted Neoepitopes and mIHC Gating, Related to Figure 4.
(A) Upset plot showing intersection of neoepitopes predicted to bind to at least one patient MHC allele. (B-E) Hierarchical gating via image cytometry for identification of cell types and functional states elaborated by mIHC for: (B) myeloid, (C) lymphoid, (D) functional, and (E) discovery panel of antibodies.

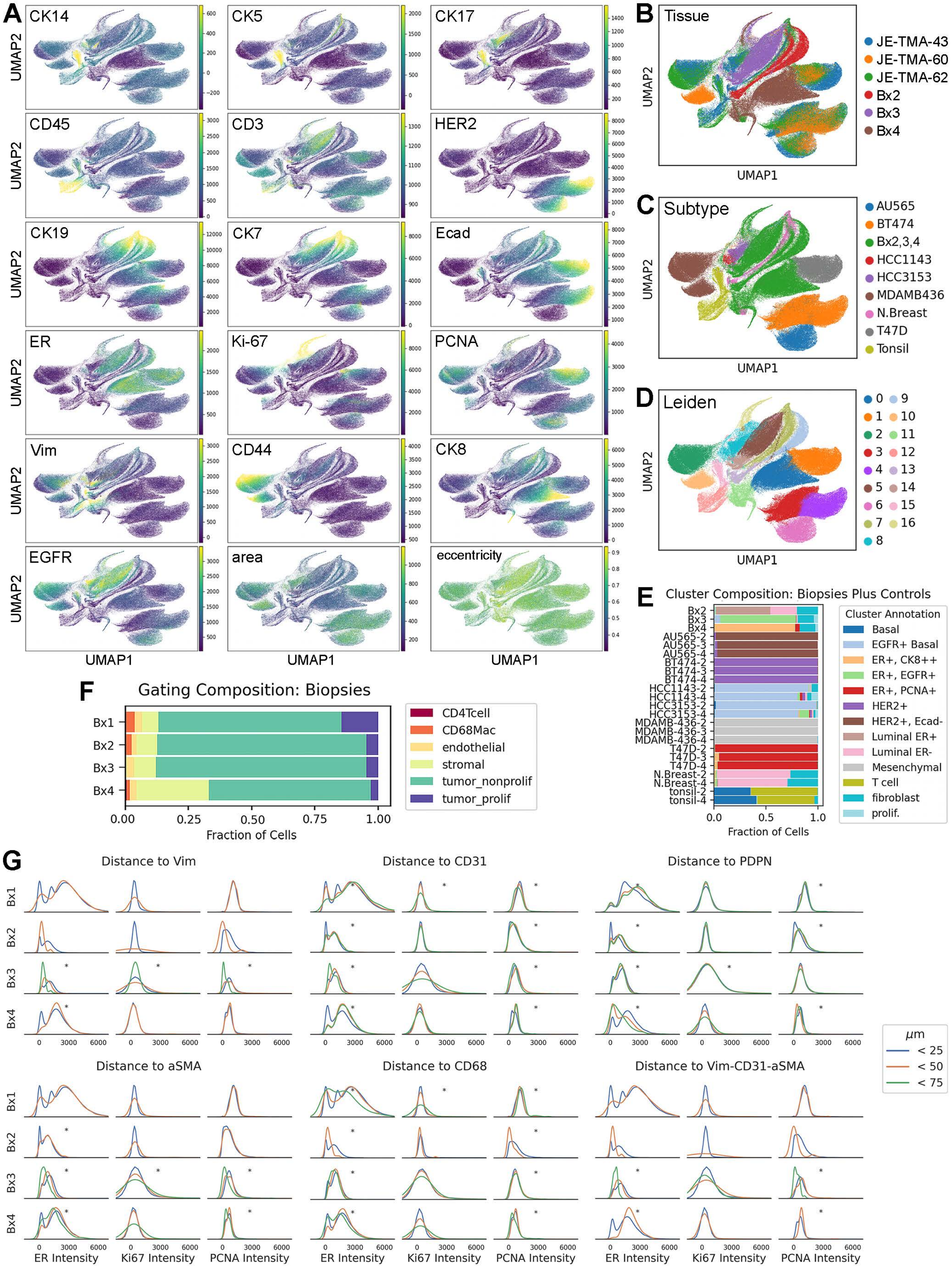

Figure S5. Additional CycIF Analyses, Related to Figure 5.

(A) Umap projection of 18 features used for clustering, colored by feature. (B) Tissues included in cluster analysis. Controls for each biopsy are as follows, Bx2: JE-TMA-43, Bx3: JE-TMA-60, Bx4: JE-TMA-62. TMAs were used for normalization, so mixing of cells from all TMAs indicates successful normalization. (C) Umap colored by cell lines and normal tissues included in clustering. (D) Umap projection colored by Leiden clustering result. (E) Composition of biopsies and

controls by annotated Leiden cluster. Number following cell line and tissue names indicates the biopsy that was paired with those controls. (F) Composition of biopsies based on manual thresholding and gating (see methods). (G) Intensity of ER, Ki67, and PCNA at 0-25, 25-50, and 50-75 μm away from various markers. Vim = vimentin, in mesenchymal cells such as fibroblasts; aSMA = Alpha Smooth Muscle Actin expressed in fibroblasts, pericytes and myoepithelial cells; PDPN = podoplanin, expressed in fibroblasts and lymphatic vessels; CD31= cluster of differentiation 31, expressed in endothelial cells; CD68 = Cluster of Differentiation 68, expressed by monocytes and macrophages; Vim-CD31-aSMA = pixels positive for any of vimentin, CD31, aSMA. Asterisks indicate significant ($p < 0.001$) difference in mean intensity between distances (ANOVA).

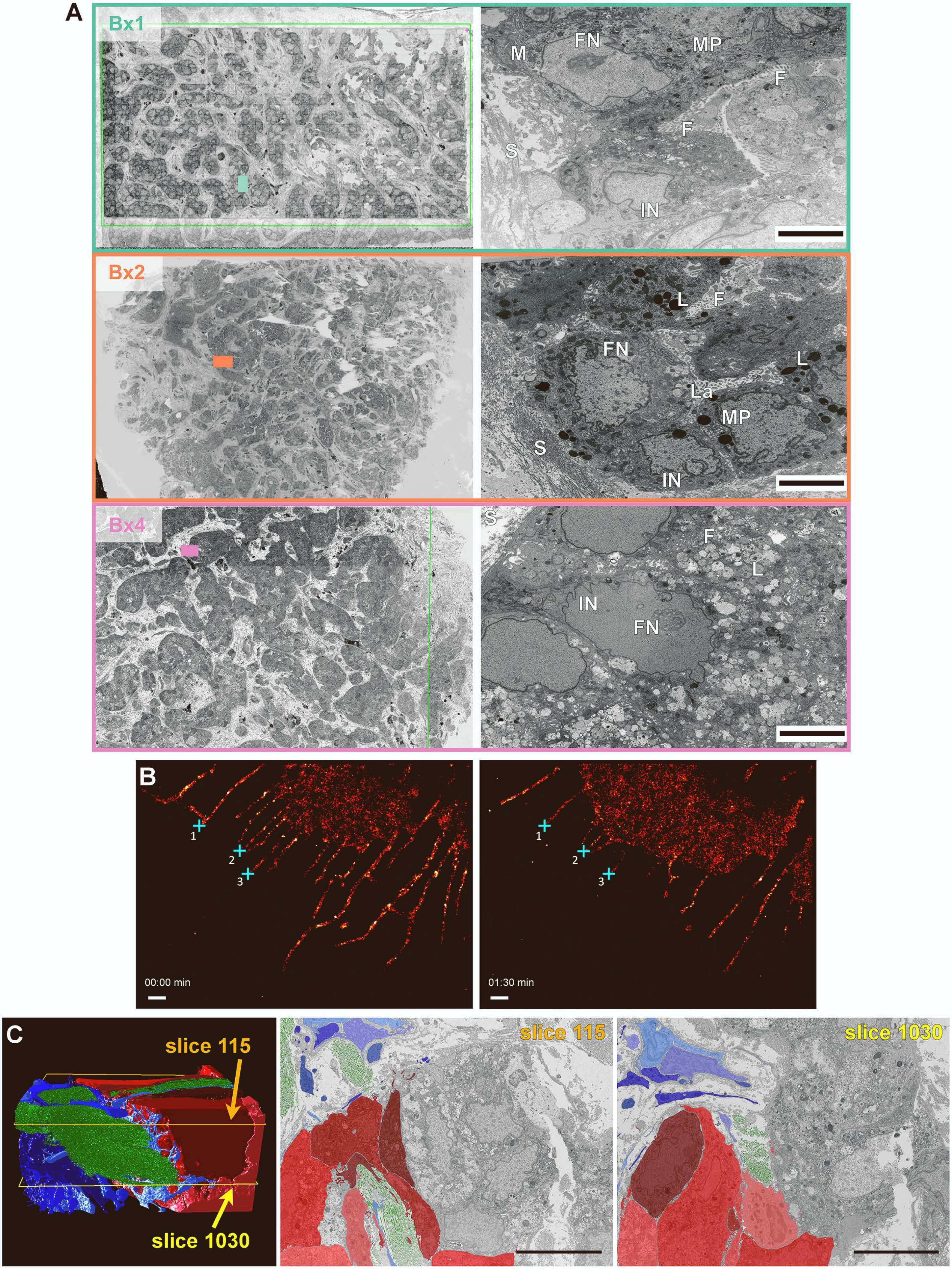

A
Bx1
M
FN
MP
F
F
S
IN
Bx2
L
F
FN
L
La
MP
S
IN
Bx4
S
F
L
IN
FN
B
1
2
3
00:00 min
1
2
3
01:30 min
C
slice 115
slice 1030
slice 115
slice 1030

Figure S6. 2-D SEM, STORM, and FIB-SEM imaging, Related to Figure 6.
(A) The left column shows top-down, high-resolution blockface maps collected via 2D SEM from Bx1, Bx2, and Bx4. The boxes marked on the maps measure 25 μm in the long direction and indicate where 3D FIB-SEM was collected. The right column shows the respective first slice from the FIB-SEM volumes. Ultrastructural features of interest are marked as the following: (IN) invaginated nuclei, (FN) fenestrated nucleoli, (M) mitochondria, (L) lysosome, (S) stroma, (F) filopodia, (La) lamellipodia, (MP) macropinosomes. Scale bars, 4 μm. Bx1 shows well-defined nests of tumor cells separated by thick bands of collagen. Bx2 also shows tumor cell nests, but the tissue is denser and collagen band thickness is reduced. Bx4 shows a return to thick stromal bands and clear tumor cell nest formation, but the high-resolution view on the right shows ultrastructure different to Bx1, particularly with respect to lysosomes and macropinosomes. (B) Filopodia-like protrusions (FLPs) direct EGF-induced cell movement of SKBR3 cells. Time-lapse stochastic optical reconstruction microscopy (STORM) images of SKBR3 cells labeled with Alexa Fluor 647-conjugated Herceptin, showing abundant FLPs decorated by HER2. Left image shows initial positions of the FLPs upon addition of EGF (10 ng/mL at around -30 s). Right image shows the same field of view after 1:30 min (90 s). The cyan crosses (1, 2, and 3) mark the original locations of the tips of corresponding FLPs. Scale bars, 1 mm. (C) Segmentation of any imaging modality is limited by the 2D plane being viewed. As shown by the FIB-SEM volume, the organization, number, and area of tumor cells (red), stromal cells (blue), and collagen (green), are different depending on the depth (image slice number) of the 2D plane within the tissue sample. Slices 115 and 1030 are separated by ~9 μm. Scale bars, 10 μm.

Supplemental References

S1. Hanker, A.B., Sudhan, D.R., and Arteaga, C.L. (2020). Overcoming Endocrine Resistance in Breast Cancer. Cancer Cell 37, 496-513.

S2. Shiino, S., Kinoshita, T., Yoshida, M., Jimbo, K., Asaga, S., Takayama, S., and Tsuda, H. (2016). Prognostic Impact of Discordance in Hormone Receptor Status Between Primary and Recurrent Sites in Patients With Recurrent Breast Cancer. Clin. Breast Cancer 16, e133-140.

S3. O'Leary, B., Cutts, R.J., Liu, Y., Hrebien, S., Huang, X., Fenwick, K., Andre, F., Loibl, S., Loi, S., Garcia-Murillas, I., et al. (2018). The Genetic Landscape and Clonal Evolution of Breast Cancer Resistance to Palbociclib plus Fulvestrant in the PALOMA-3 Trial. Cancer Discov. 8, 1390-1403.

S4. Razavi, P., Chang, M.T., Xu, G., Bandlamudi, C., Ross, D.S., Vasan, N., Cai, Y., Bielski, C.M., Donoghue, M.T.A., Jonsson, P., et al. (2018). The Genomic Landscape of Endocrine-Resistant Advanced Breast Cancers. Cancer Cell 34, 427-438 e426.

S5. Nayar, U., Cohen, O., Kapstad, C., Cuoco, M.S., Waks, A.G., Wander, S.A., Painter, C., Freeman, S., Persky, N.S., Marini, L., et al. (2019). Acquired HER2 mutations in ER(+) metastatic breast cancer confer resistance to estrogen receptor-directed therapies. Nat. Genet. 51, 207-216.

S6. Giltnane, J.M., Hutchinson, K.E., Stricker, T.P., Formisano, L., Young, C.D., Estrada, M.V., Nixon, M.J., Du, L., Sanchez, V., Ericsson, P.G., et al. (2017). Genomic profiling of ER(+) breast cancers after short-term estrogen suppression reveals alterations associated with endocrine resistance. Sci. Transl. Med. 9.

S7. Levine, K.M., Priedigkeit, N., Basudan, A., Tasdemir, N., Sikora, M.J., Sokol, E.S., Hartmaier, R.J., Ding, K., Ahmad, N.Z., Watters, R.J., et al. (2019). FGFR4 overexpression and hotspot mutations in metastatic ER+ breast cancer are enriched in the lobular subtype. NPJ Breast Cancer 5, 19.

S8. Fu, X., Creighton, C.J., Biswal, N.C., Kumar, V., Shea, M., Herrera, S., Contreras, A., Gutierrez, C., Wang, T., Nanda, S., et al. (2014). Overcoming endocrine resistance due to reduced PTEN levels in estrogen receptor-positive breast cancer by co-targeting mammalian target of rapamycin, protein kinase B, or mitogen-activated protein kinase kinase. Breast Cancer Res. 16, 430.

S9. Pearson, A., Proszek, P., Pascual, J., Fribbens, C., Shamsher, M.K., Kingston, B., O'Leary, B., Herrera-Abreu, M.T., Cutts, R.J., Garcia-Murillas, I., et al. (2020). Inactivating NF1 Mutations Are Enriched in Advanced Breast Cancer and Contribute to Endocrine Therapy Resistance. Clin. Cancer Res. 26, 608-622.

S10. Lu, R., Hu, X., Zhou, J., Sun, J., Zhu, A.Z., Xu, X., Zheng, H., Gao, X., Wang, X., Jin, H., et al. (2016). COPS5 amplification and overexpression confers tamoxifen-resistance in ERalpha-positive breast cancer by degradation of NCoR. Nat Commun 7, 12044.

S11. Gupta, A., Hossain, M.M., Miller, N., Kerin, M., Callagy, G., and Gupta, S. (2016). NCOA3 coactivator is a transcriptional target of XBP1 and regulates PERK-eIF2alpha-ATF4 signalling in breast cancer. Oncogene 35, 5860-5871.

S12. Haricharan, S., Punturi, N., Singh, P., Holloway, K.R., Anurag, M., Schmelz, J., Schmidt, C., Lei, J.T., Suman, V., Hunt, K., et al. (2017). Loss of MutL Disrupts CHK2-Dependent Cell-Cycle Control through CDK4/6 to Promote Intrinsic Endocrine Therapy Resistance in Primary Breast Cancer. Cancer Discov. 7, 1168-1183.

S13. Hinohara, K., Wu, H.J., Vigneau, S., McDonald, T.O., Igarashi, K.J., Yamamoto, K.N., Madsen, T., Fassl, A., Egri, S.B., Papanastasiou, M., et al. (2018). KDM5 Histone Demethylase Activity Links Cellular Transcriptomic Heterogeneity to Therapeutic Resistance. Cancer Cell 34, 939-953 e939.

S14. Simoes, B.M., O'Brien, C.S., Eyre, R., Silva, A., Yu, L., Sarmiento-Castro, A., Alferez, D.G., Spence, K., Santiago-Gomez, A., Chemi, F., et al. (2015). Anti-estrogen Resistance in Human Breast Tumors Is Driven by JAG1-NOTCH4-Dependent Cancer Stem Cell Activity. Cell Rep. 12, 1968-1977.

S15. Alves, C.L., Elias, D., Lyng, M.B., Bak, M., and Ditzel, H.J. (2018). SNAI2 upregulation is associated with an aggressive phenotype in fulvestrant-resistant breast cancer cells and is an indicator of poor response to endocrine therapy in estrogen receptor-positive metastatic breast cancer. Breast Cancer Res. 20, 60.

S16. Bacci, M., Lorito, N., Ippolito, L., Ramazzotti, M., Luti, S., Romagnoli, S., Parri, M., Bianchini, F., Cappellesso, F., Virga, F., et al. (2019). Reprogramming of Amino Acid Transporters to Support Aspartate and Glutamate Dependency Sustains Endocrine Resistance in Breast Cancer. Cell Rep. 28, 104-118 e108.

S17. Yang, J., AlTahan, A., Jones, D.T., Buffa, F.M., Bridges, E., Interiano, R.B., Qu, C., Vogt, N., Li, J.L., Baban, D., et al. (2015). Estrogen receptor-alpha directly regulates the hypoxia-inducible factor 1 pathway associated with antiestrogen response in breast cancer. Proc. Natl. Acad. Sci. U. S. A. 112, 15172-15177.

S18. Dunbier, A.K., Ghazoui, Z., Anderson, H., Salter, J., Nerurkar, A., Osin, P., A'Hern, R., Miller, W.R., Smith, I.E., and Dowsett, M. (2013). Molecular profiling of aromatase inhibitor-treated postmenopausal breast tumors identifies immune-related correlates of resistance. Clin. Cancer Res. 19, 2775-2786.

S19. Stender, J.D., Nwachukwu, J.C., Kastrati, I., Kim, Y., Strid, T., Yakir, M., Srinivasan, S., Nowak, J., Izard, T., Rangarajan, E.S., et al. (2017). Structural and Molecular Mechanisms of Cytokine-Mediated Endocrine Resistance in Human Breast Cancer Cells. Mol. Cell 65, 1122-1135 e1125.

S20. Joffroy, C.M., Buck, M.B., Stope, M.B., Popp, S.L., Pfizenmaier, K., and Knabbe, C. (2010). Antiestrogens induce transforming growth factor beta-mediated immunosuppression in breast cancer. Cancer Res. 70, 1314-1322.
