# Supplementary material for: An omic and multidimensional spatial atlas from serial biopsies of an evolving metastatic breast cancer
Source: Cell Rep Med. 2022 Feb 15;3(2):100525. doi: 10.1016/j.xcrm.2022.100525 (PMC8861971; doi:10.1016/j.xcrm.2022.100525)
Supplement: Document S2. Article plus supplemental information [file mmc9.pdf]

Cell Reports Medicine

Article

An omic and multidimensional spatial atlas from serial biopsies of an evolving metastatic breast cancer

Graphical abstract

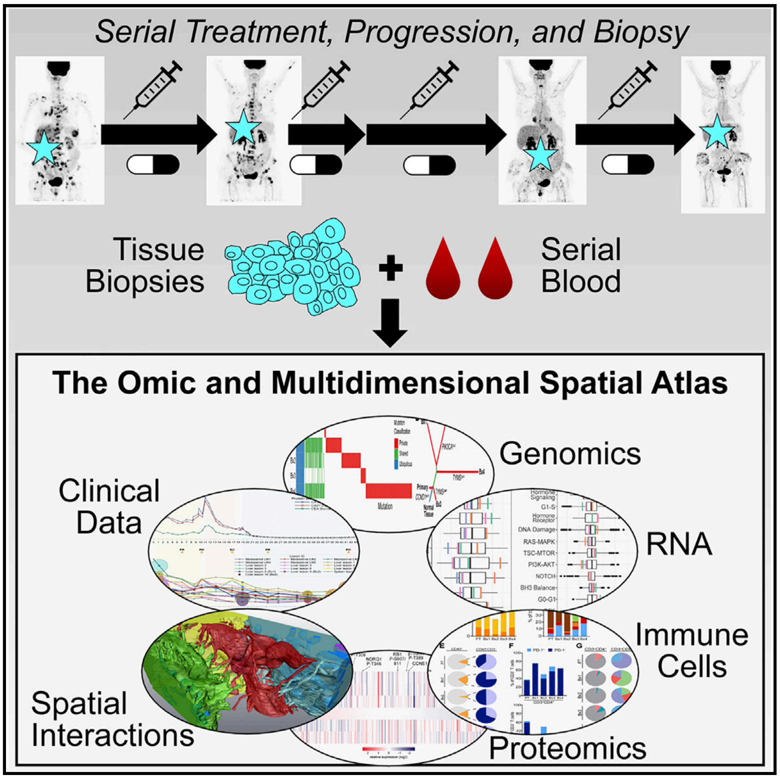

Highlights

- Safe and reliable workflows for multiplatform measurements from single biopsies
- Clinical metadata with 11 omic and imaging assays from serial biopsy and blood
- Omic, cellular, and structural evolution of metastatic cancer in a single individual
- Integrative analyses reveal new potential mechanisms of response and resistance

Authors

Brett E. Johnson, Allison L. Creason, Jayne M. Stommel, ..., Zahi Mitri, Gordon B. Mills, Joe W. Gray

Correspondence

grayjo@ohsu.edu

In brief

Identifying mechanisms of response and resistance to treatment in individual cancer patients is challenging but critical for improvement of precision medicine outcomes. Johnson et al. report a comprehensive atlas from a single individual with breast cancer and demonstrate how longitudinal, integrative analyses can provide new insights.

Johnson et al., 2022, Cell Reports Medicine *3*, 100525
February 15, 2022 © 2022 The Authors.
https://doi.org/10.1016/j.xcrm.2022.100525

CellPress

Cell Reports Medicine

CellPress OPEN ACCESS

Article

An omic and multidimensional spatial atlas from serial biopsies of an evolving metastatic breast cancer

Brett E. Johnson,[1,2] Allison L. Creason,[1,2] Jayne M. Stommel,[1,2] Jamie M. Keck,[1] Swapnil Parmar,[1] Courtney B. Betts,[1,3] Aurora Blucher,[1,3] Christopher Boniface,[2,4] Elmar Bucher,[2] Erik Burlingame,[2,5] Todd Camp,[1] Koei Chin,[1,2] Jennifer Eng,[2] Joseph Estabrook,[1,6] Heidi S. Feiler,[1,2] Michael B. Heskett,[6] Zhi Hu,[1,2] Annette Kolodzie,[1] Ben L. Kong,[1,7] Marilyne Labrie,[1,3] Jinho Lee,[1] Patrick Leyshock,[1] Souraya Mitri,[1] Janice Patterson,[1,8] Jessica L. Riesterer,[2,9] Shamilene Sivagnanam,[1,3,5] Julia Somers,[1,6] Damir Sudar,[10] Guillaume Thibault,[2] Benjamin R. Weeder,[2] Christina Zheng,[1] Xiaolin Nan,[2,4] Reid F. Thompson,[2,11] Laura M. Heiser,[1,2] Paul T. Spellman,[2,6] George Thomas,[1,12] Emek Demir,[1,6] Young Hwan Chang,[2,5] Lisa M. Coussens,[1,3] Alexander R. Guimaraes,[1,13] Christopher Corless,[7,12] Jeremy Goecks,[1,2] Raymond Bergan,[14] Zahi Mitri,[15,16] Gordon B. Mills,[1,3] and Joe W. Gray[1,2,17,*]

[1]Knight Cancer Institute, Oregon Health & Science University, Portland, OR 97239, USA
[2]Department of Biomedical Engineering, Oregon Health & Science University, Portland, OR 97239, USA
[3]Department of Cell, Developmental & Cancer Biology, Oregon Health & Science University, Portland, OR 97239, USA
[4]Cancer Early Detection Advanced Research Center, Oregon Health & Science University, Portland, OR 97239, USA
[5]Computational Biology Program, Oregon Health & Science University, Portland, OR 97239, USA
[6]Department of Molecular and Medical Genetics, Oregon Health & Science University, Portland, OR 97239, USA
[7]Department of Pharmacy Services, Oregon Health & Science University, Portland, OR 97239, USA
[8]Knight Diagnostic Laboratories, Oregon Health & Science University, Portland, OR 97239, USA
[9]Multiscale Microscopy Core, Oregon Health & Science University, Portland, OR 97239, USA
[10]Quantitative Imaging Systems LLC, Portland, OR 97239, USA
[11]Division of Hospital and Specialty Medicine, VA Portland Healthcare System, Portland, OR 97239, USA
[12]Department of Pathology & Laboratory Medicine, Oregon Health & Science University, Portland, OR 97239, USA
[13]Department of Diagnostic Radiology, Oregon Health & Science University, Portland, OR 97239, USA
[14]Fred & Pamela Buffett Cancer Center, University of Nebraska Medical Center, Omaha, NE 68198, USA
[15]Division of Hematology & Medical Oncology, Knight Cancer Institute, Oregon Health & Science University, Portland, OR 97239, USA
[16]Department of Medicine, Knight Cancer Institute, Oregon Health & Science University, Portland, OR 97239, USA
[17]Lead contact
*Correspondence: grayjo@ohsu.edu
https://doi.org/10.1016/j.xcrm.2022.100525

SUMMARY

Mechanisms of therapeutic resistance and vulnerability evolve in metastatic cancers as tumor cells and extrinsic microenvironmental influences change during treatment. To support the development of methods for identifying these mechanisms in individual people, here we present an omic and multidimensional spatial (OMS) atlas generated from four serial biopsies of an individual with metastatic breast cancer during 3.5 years of therapy. This resource links detailed, longitudinal clinical metadata that includes treatment times and doses, anatomic imaging, and blood-based response measurements to clinical and exploratory analyses, which includes comprehensive DNA, RNA, and protein profiles; images of multiplexed immunostaining; and 2- and 3-dimensional scanning electron micrographs. These data report aspects of heterogeneity and evolution of the cancer genome, signaling pathways, immune microenvironment, cellular composition and organization, and ultrastructure. We present illustrative examples of how integrative analyses of these data reveal potential mechanisms of response and resistance and suggest novel therapeutic vulnerabilities.

INTRODUCTION

Precision medicine has led to substantial improvements in clinical outcomes for some individuals with cancer, increasingly through use of analytical procedures that identify people with molecular characteristics associated with an increased likelihood of response.[1,2] Unfortunately, treatments deployed according to precision medicine principles do not always elicit positive responses, and durable control is achieved for only a subset of individuals with metastatic cancer.[3] We posit that the failure to control individual cancers using biomarker-guided treatments stems in large part from our imperfect understanding of the multitude of resistance mechanisms that drive an individual tumor's adaptive ability to survive as they evolve under therapy. These mechanisms may involve regulatory networks intrinsic to tumor cells, chemical and mechanical influences from proximal

© 2022 The Authors.
This is an open access article under the CC BY license (http://creativecommons.org/licenses/by/4.0/).

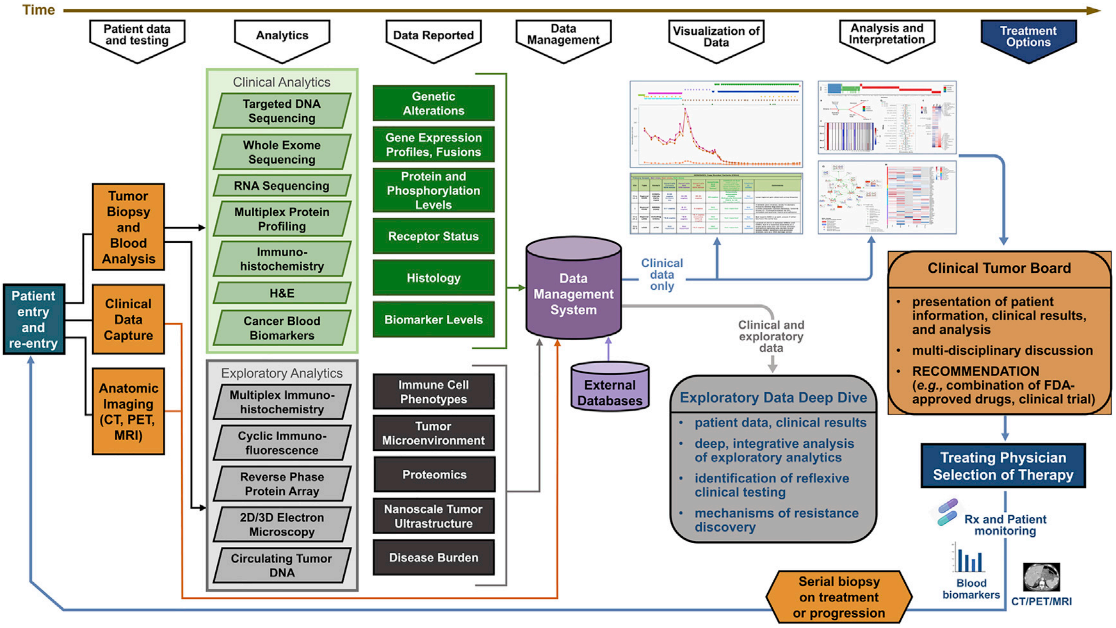

Figure 1. Workflows and analytical platforms used to generate the OMS atlas

or distal microenvironments, and/or aspects of the immune system. They may vary between individuals with similar biomarkers, across metastases within a person, or among cell subpopulations within a single lesion and may change during treatment.

To stimulate and support community-wide investigations of cancer resistance, response, and evolutionary mechanisms in individuals, we present a comprehensive omic and multidimensional spatial (OMS) atlas composed of clinical and research data, response correlates from an affected individuals, and illustrative analytical workflows from a single person with metastatic breast cancer during 3.5 years of treatment. We also illustrate how the atlas can be used to explore spatial and temporal heterogeneity, study tumor evolution, and identify candidate resistance mechanisms and therapeutic vulnerabilities. These studies were carried out in the Serial Measurements of Molecular and Architectural Responses to Therapy (SMMART) program with support from the Human Tumor Atlas Network (HTAN),[4,5] through which all data are available in standardized formats.

RESULTS

Longitudinal data generation from a single individual

The focus of this OMS atlas is a female individual diagnosed with hormone receptor-positive, HER2-normal, high-OncotypeDx recurrence score,[6] 0.6-cm right breast ductal carcinoma at the age of 64. She underwent a lumpectomy with intra-operative radiation therapy, followed by treatment with four cycles of adjuvant docetaxel and cyclophosphamide, 2 years of anastrozole, and 5 years of exemestane. Subsequent computed tomography (CT) and fluorodeoxyglucose-positron emission tomography (FDG-PET) scans revealed widespread metastatic disease.

The individual was then enrolled in the SMMART program (Figure 1). Management decisions were made by the treating physician based on all clinical information plus input from a multidisciplinary tumor board (STAR Methods). This led to four treatment phases over a 3.5-year period (Figure 2A). Temporary tumor control was achieved in the first three phases, with a new phase of therapy beginning at signs of progression. Toxicity of the combination therapies was effectively managed through supportive medication and dose reduction (Figure S2A). Standard toxicity-related blood chemistries were monitored, including absolute neutrophil and platelet counts and liver function tests (Figures S2B–S2D; Table S1).

The clinical metadata in Table S1 link detailed treatment doses and timelines (Figure 2A) to tumor response metrics. Serum levels of the tumor protein biomarkers carcinoembryonic antigen (CEA), cancer antigen 15-3 (CA 15-3), and cancer antigen 27-29 (CA 27-29) were routinely measured to monitor treatment response (Figure 2B; Table S1). Increasing biomarker levels are concerning for underlying progression, but National Comprehensive Cancer Network (NCCN) guidelines do not recommend changing therapies solely based on blood biomarkers.[7] Biomarker measurements were thus complemented by periodic CT and FDG-PET imaging, with response evaluated using response evaluation criteria in solid tumors (RECIST) 1.1 criteria[8] (Figure 2C; Table S1). Representative computed tomography (CT), fluorodeoxyglucose-positron emission tomography (FDG-PET), and ultrasound images highlight disease burden at key time points (Figure S1).

Biospecimens collected for analysis include serial blood samples, a primary breast tumor (PT), a liver biopsy taken immediately prior to phase 1 (Bx1), a biopsy of a different liver lesion taken at the end of phase 1 (Bx2), a bone lesion biopsy taken at the end of phase 3 (Bx3), and a biopsy of a third liver lesion taken at the end of phase 4 (Bx4; Figure 2A). Importantly, Bx2–

CellPress
OPEN ACCESS

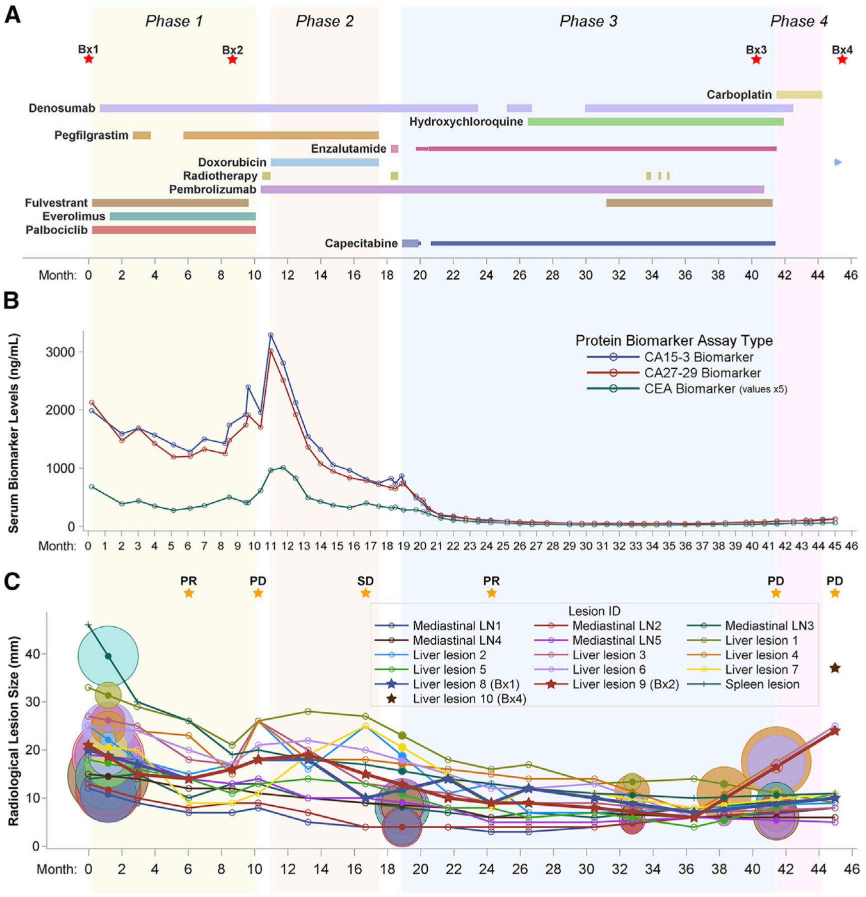

Figure 2. Timeline of clinical treatment and response metrics

(A) Treatment schedule and biopsy timing (red stars) over four phases of treatment (green, orange, blue, and pink areas). The timeline is sectioned into 28-day months. The duration and relative dose for each drug is indicated by the extent and width of a horizontal bar. Drug continuation after the end of phase 4 is indicated by a right arrow.

(B) Clinically reported serum levels of tumor protein biomarkers. CEA values were multiplied by 5 to ease visualization.

(C) RECIST 1.1 assessment of tumor response (orange stars) indicating partial response (PR), progressive disease (PD), or stable disease (SD). Shown are longitudinal tracking and variation in the longest-axis size of 16 representative metastatic lesions measured from serial CT images. Targets of metastatic biopsies are bolded and marked with stars. Circles represent FDG-PET imaging results, colored and centered on the lines of their corresponding lesion at interpolated lesion sizes. The diameter of each circle is proportional to the background-normalized maximum standardized uptake value (SUVmax).

Bx4 were acquired from metastatic lesions explicitly identified on serial CT and/or FDG-PET imaging as progressing near the end of each respective treatment phase (Figures 2A and S1A–S1I). These biospecimens were analyzed using 11 distinct omic and multiscale spatial imaging workflows to generate this OMS atlas (Figure 1).

Genomic differences between metastases were substantial

Targeted DNA sequencing (GeneTrails solid tumor panel, all biopsies), whole-exome sequencing (WES; primary tumor and Bx1–Bx4), and low-pass whole genome sequencing (LP-WGS; Bx3 and Bx4) were used to identify somatic genomic alterations, including single-nucleotide variants (SNVs), insertions or deletions (indels), and copy number changes (Figures 3A and S3A). Ubiquitous alterations included amplification of the CDK4/6 regulatory partner cyclin D1 (*CCND1*; Figure S3B). Other biologically and clinically relevant alterations were private to a single biopsy. For example, Bx2 contained a hotspot *PIK3CA* mutation (p.E542K; GenBank: NM_006218:c.1624G>A)[9,10] that was absent from other samples and was taken from a liver lesion that increased in size during treatment with drugs that target aspects of phosphatidylinositol 3-kinase (PI3K) signaling (Figures 2A and 2C). Bx3 and Bx4 both harbored similar amplified regions on chromosome 18 that were not detected in prior biopsies or the primary tumor; Bx3 had 8 copies of a 2.3-Mb region, and Bx4 had 14 copies of a 0.7-Mb region (Figures 3B and S3B). These amplicons contained the genes for thymidylate synthetase (*TYMS*) and the SRC family tyrosine kinase *YES1* and were accompanied by increased *TYMS* and *YES1* RNA relative to Bx2 (*TYMS*: B×3 = 6.8×, B×4 = 7.2×; *YES1*: B×3 = 2.0×, B×4 = 4.0×). Importantly, both biopsies were acquired after treatment with the TYMS inhibitor capecitabine (Figures 2A, 2C, and S1G).

A phylogenetic analysis revealed that Bx3 diverged from the primary tumor at an earlier evolutionary stage than Bx1, Bx2, or Bx4 (Figure 3B) but was only detected on FDG-PET imaging 1 month before the biopsy occurred (Figures S1A, S1F, and S1G).

WES of circulating tumor DNA (ctDNA) from blood collected immediately prior to Bx1 (ctDNA1) and 23 days after Bx2 (ctDNA2) showed that ctDNA1 carried mutations identified previously as private to Bx2, Bx3, or Bx4, whereas ctDNA2 had mutations that were private to each of the four biopsies (Figure S3A). Thus, at least some of the genomic features detected in later biopsies were present before initiation of treatment.

Tumor mutational burden (TMB) was assessed for the primary tumor and Bx1–Bx4 because a TMB of 10 or more mutations per megabase (mut/Mb) has been associated with a positive response to immune checkpoint blockade.[11] The TMB was low overall (1.2–5.2 mut/Mb), but we identified 1,271 unique neoepitopes (158–687 neoepitopes per biopsy) predicted to bind to at least one major histocompatibility complex (MHC) allele with an affinity of less than 500 nM (Table S2). Human leukocyte

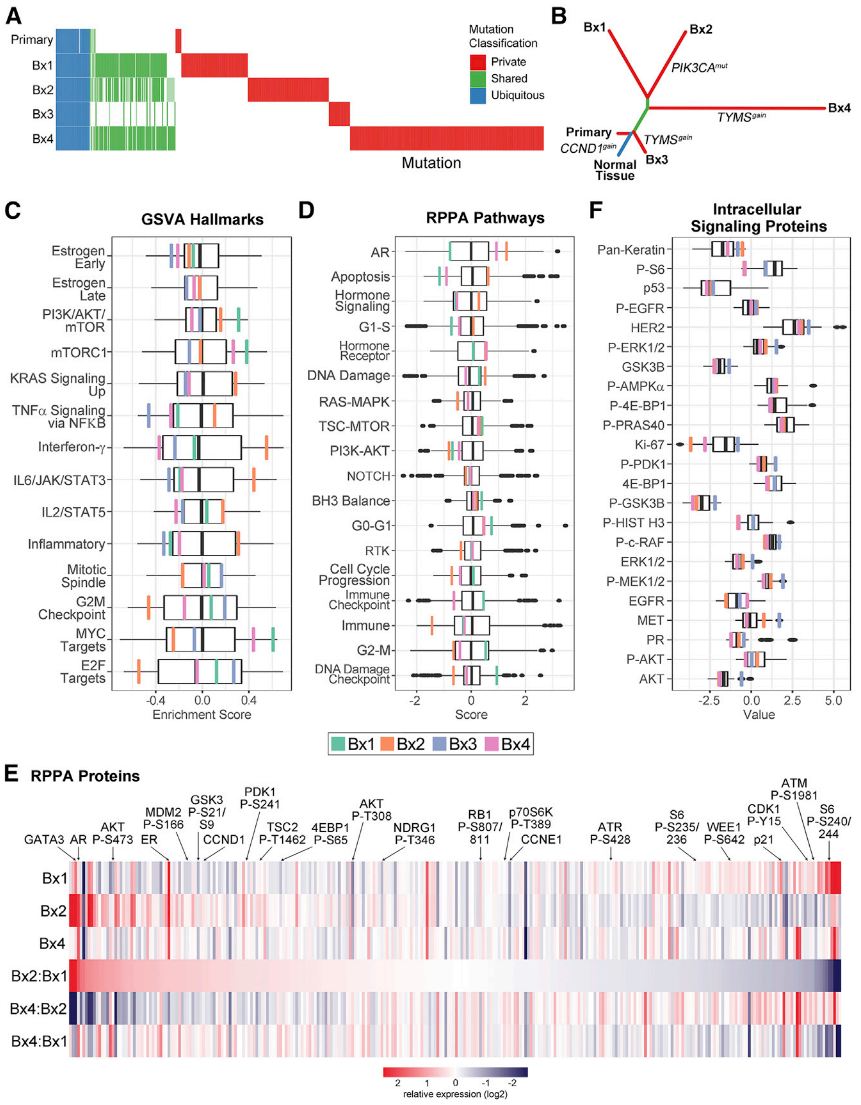

Figure 3. Genomic, transcriptomic, and proteomic profiles reveal spatiotemporal heterogeneity and evolution

(A) Comparison of somatic mutations. Columns represent individual, non-silent SNVs or indels identified from WES in at least one tissue sample and classified as ubiquitous (present in all samples, blue), shared (present in at least two samples, green), or private (present in only a single sample, red). Mutational status in each sample is indicated as independently called (colored), detected in at least 2 sequencing reads but not independently called (reduced opacity), or absent (white).

(B) Phylogenetic tree showing the evolutionary relationship between the PT and four metastases.

(C) Transcriptomic gene set variation analysis (GSVA) of cancer hallmark pathways. The boxplot represents the distribution (upper and lower quartiles and median) of GSVA scores for the TCGA luminal breast cancer cohort. Enrichment scores are shown for each of the biopsy samples: Bx1 (green), Bx2 (orange), Bx3 (blue), and Bx4 (pink).

(D) RPPA protein pathway activity assessment using pathway scores. The boxplots represent the distribution of the pathway activity of the TCGA breast cancer cohort. The pathway activities of three biopsy samples are marked as in (D).

(E) Total and phosphoprotein levels from RPPA normalized within the TCGA breast cancer cohort. The heatmap shows relative protein levels for three biopsies and the fold change between sample pairs. Proteins are ordered based on the fold change difference between Bx2 relative to Bx1. Selected proteins are highlighted.

(F) ISPP measurements of total and phosphoprotein levels. The boxplots represent the distribution of protein levels of 57 metastatic breast cancers. The protein levels of three biopsy samples are marked as in (D).

See also Figure S3 and Table S2.

antigen (HLA) subtypes were stable across all biopsies, and no loss of heterozygosity was observed. Notably, 68 neoepitopes that might serve as targets for a personalized cancer vaccine were present in the primary tumor and all four biopsies (Figure S4A).[12,13]

ctDNA increased during progression and radiotherapy

Dual index degenerate adaptor sequencing (DIDA-seq)[14] was performed, using a panel of 53 SNVs present in the individual's primary tumor, Bx1, and/or Bx2 to assess ctDNA levels from serial plasma samples collected over the first 32 months of treatment (Table S2). The average variant allele frequency (VAF) of the SNV panel remained below 0.3% of total cell free DNA during this period, with the exception of two transient increases (Figure S2E). The first occurred immediately prior to Bx2 (Figure S2E), coincident with rising CA 15-3 and CA 27-29 levels (Figure 2B), followed by progressive disease (PD; Figure 2C). The increase in ctDNA VAF was greatest for mutations shared by the primary tumor, Bx1, and Bx2 (30% VAF) compared with those private to the metastases (Bx1 and Bx2, 3.1%; Bx1, 0.05%; Bx2, 1.3%). A second ctDNA increase occurred after palliative radiation therapy to spinal lesions at C2–C5. Interestingly, the VAFs of all SNV groups in the panel increased at this time, including those private to Bx1 and Bx2 liver lesions.

Signaling and pathway activities evolved during therapy

Signaling and pathway activities were calculated from whole-transcriptome sequencing (RNA sequencing [RNA-seq]). Classification using the PAM50 subtype gene signature[15] showed liver biopsies Bx1, Bx2, and Bx4 to be luminal A, whereas the bone biopsy Bx3 was luminal B (Figure S3C). Table S2 summarizes RNA transcript levels and pathway activity estimates for Bx1–Bx4 relative to breast cancers in The Cancer Genome Atlas

(TCGA-BRCA)[16] and gene set variation analysis (GSVA) of enriched Molecular Signatures Database (MSigDB) cancer hallmarks relative to TCGA-BRCA luminal samples.[17,18] Proliferation, Immune, and Signaling were the most variable (MSigDB) Hallmark Process categories across the biopsies (Figure 3C). Notably, Bx2 harboring the *PIK3CA* p.E542K mutation had reduced "PI3K/AKT/mTOR" compared with Bx1, although that gene set was still increased relative to TCGA-BRCA samples.

Protein and phosphoprotein abundances were measured in Bx1, Bx2, and Bx4 using reverse-phase protein arrays (RPPAs), and proteomic pathway signatures were compared with TCGA-BRCA (Figures 3D and 3E).[19–22] Aspects of hormone signaling varied across biopsies. Estrogen receptor (ER) protein levels from RPPA and clinical immunohistochemistry (IHC) were high in all three biopsies (Table S1). The protein pathways "hormone signaling" and "hormone receptor" were higher in Bx2 (Figure 3D), whereas the GSVA RNA hallmarks "estrogen early" and "estrogen late" (Figure 3C) showed little change, an intriguing finding because protein levels of the hormone-regulated transcription factors ER, GATA3, and adrenergic receptor (AR) were increased in Bx2 relative to Bx1 after phase 1 treatments (Figure 3E). Bx4, taken after phase 4 treatment without hormone suppression, showed continued elevation of the "hormone receptor" pathway and ER and AR protein levels relative to Bx1. However, GATA3 protein levels, the "hormone signaling" protein pathway, and the "estrogen early" and "estrogen late" GSVA hallmarks were downregulated.

PI3K/AKT/mTOR pathway signaling from RPPA was generally similar across all biopsies, even though Bx2 was collected after treatment with the mTORC1 inhibitor everolimus and contained the hotspot mutation *PIK3CA* p.E542K (Figure 3D). Individual protein levels within these pathways varied but did not result in changes in overall signaling. For example, Bx2 showed decreased mTORC1 complex activity based on decreased S6 phosphorylation at S235/236 and S240/244 (0.7× and 0.1× versus Bx1) but increased activity downstream of mTORC2, including increased phosphorylation of AKT (S473: 2.7× versus Bx1) and its substrates GSK3A/B (S21/S9: 1.7× versus Bx1), TSC2 (T1462: 1.4× versus Bx1), and MDM2 (S166: 1.8× versus Bx1; Figure 3E). Likewise, Bx4 showed increased phosphorylation of AKT at S473 (2.7× versus Bx1, 1.0× versus Bx2) and NDRG1 (T346: 1.8× versus Bx1, 1.6× versus Bx2) but without an accompanying increase in AKT or mTORC1 substrate phosphorylation. We also used the clinical Intracellular Signaling Protein Panel (ISPP) to quantitate phosphoproteins and total proteins in Bx2–Bx4 (Figure 3F; Table S2).[23] ISPP showed that Bx2 had the highest AKT phosphorylation and lowest S6 phosphorylation relative to the other biopsies, consistent with RPPA results, whereas p-ERK, p-cRAF, and p-MEK were elevated in Bx3 and increased relative to other biopsies.

A transcriptional regulator analysis using a molecular interactions network derived from Pathway Commons[24] was used to infer regulator protein activity from the gene expression data. Integrative analysis of the longitudinal changes in proteomics, gene expression, and transcriptional regulator scores between Bx1 and Bx2 was also performed using CausalPath (Figure S3D).[25] These analyses showed strong inhibition of mTOR regulator activity (Bx2 5.1× > Bx1). Activities of multiple JAK-STAT family proteins were increased, including JAK2 (Bx2 1.8× > Bx1), phospho-STAT3 (Y705: Bx2 1.6× > Bx1), and STAT5 (Bx2 3.2× > Bx1), which, together with the oncoprotein mucin 1 (MUC1; protein Bx2 27.1× > Bx1; regulator Bx2 +3.15 versus Bx1) constitute a known feedforward loop whereby MUC1 binds STAT3 to facilitate its phosphorylation by JAK1.[26] These observations are reinforced by elevation in "IL6/JAK/STAT3" and "IL2/STAT5" signaling from GSVA (Figure 3C). This analysis also highlighted decreases in MYC and E2F regulator activity and E2F1 total protein, consistent with decreased enrichment of "MYC targets" and "E2F1 targets" in GSVA. These analyses provide a view of the interaction dynamics of cell cycle control networks, with decreases in the expression of cell cycle progression genes (*CCNB1*, *CDK4*, *CDK1*, *CCNE2*, *CCND3*, and *PLK1*) balanced by a sharp decrease in cell cycle inhibitor genes (*CDKN1A*, *CDKN1B*, and *CDKN2A*), leaving RB1 phosphorylation unchanged in Bx2.

Tumor immune microenvironment evolution and barriers to T cell activation

Changes in composition and functionality of lymphoid and myeloid lineage immune cells were assessed in the primary tumor and Bx1–Bx4 using multiplex IHC (mIHC),[27–29] noting possible discordance in Bx3 because of its bone origin (Figures 4 and S4B–S4E; Table S2). Total immune cell infiltration, as indicated by the percentage of $CD45^+$ cells, was lowest in Bx2 (2.1%) but comparable between the PT (7.2%), Bx1 (9.3%), and Bx4 (10.0%; Figures 4A and 4B). Myelomonocytic cells (macrophages and monocytes) comprised the dominant $CD45^+$ leukocyte lineage subgroup in the PT (65.8%; Figure 4C, green and brown), Bx1 (48.3%), and Bx2 (82.4%) and were reduced in Bx4 (6.1%). Analysis of the myeloid lineage revealed that the fraction of immature dendritic cells was higher in Bx1 (0.2%; Figure 4D) than in Bx2 (0.05%), whereas the proportions of $CD163^+$ and $CD163^-$ macrophages and monocytes were higher in Bx2 (51.2%) than in Bx1 (18.9%), with the largest increase in $CD163^+$ macrophages (Figure 4D). CD163 positivity is associated with differentiation of myelomonocytic cells toward an alternatively activated or "M2"-type state, which is considered to be pro-tumorigenic within solid tumors.[30,31] CD163 expression on monocytes and macrophages is induced by interleukin-10 (IL-10) and glucocorticoids and repressed by lipopolysaccharides, tumor necrosis factor alpha (TNF-α), and interferon γ (IFNγ) and is concordant with upregulation of IL-containing GSVA gene sets in Bx2 (Figure 3C; Table S2).[32] The dominance of macrophages and monocytes and relative lack of T cells in the PT, Bx1, and Bx2 was in stark contrast to Bx4, which had many more T cells than macrophages and monocytes (PT: 65.8% macrophages/monocytes, 10.9% T cells; Bx1: 48.3%, 20.6%; Bx2: 82.4%, 5.3%; Bx4: 6.1%, 33.1%; Figures 4C and 4E, orange).

Analyses of T cell subsets and functionality showed that only a small fraction of $CD3^+CD4^+$ and $CD3^+CD8^+$ T cells in the PT, Bx1, or Bx4 expressed the programmed cell death-1 (PD-1) protein, which is typically expressed on activated T cells following T cell priming or persistent antigen exposure (PT: 1.5% $CD4^+$ T cells, 4.1% $CD8^+$ T cells; Bx1: 1.3%, 1.6%; Bx4: 0%, 0.7%; compared with Bx2: 10.1%, 30.5%; Bx3: 11.3%, 6.3%;

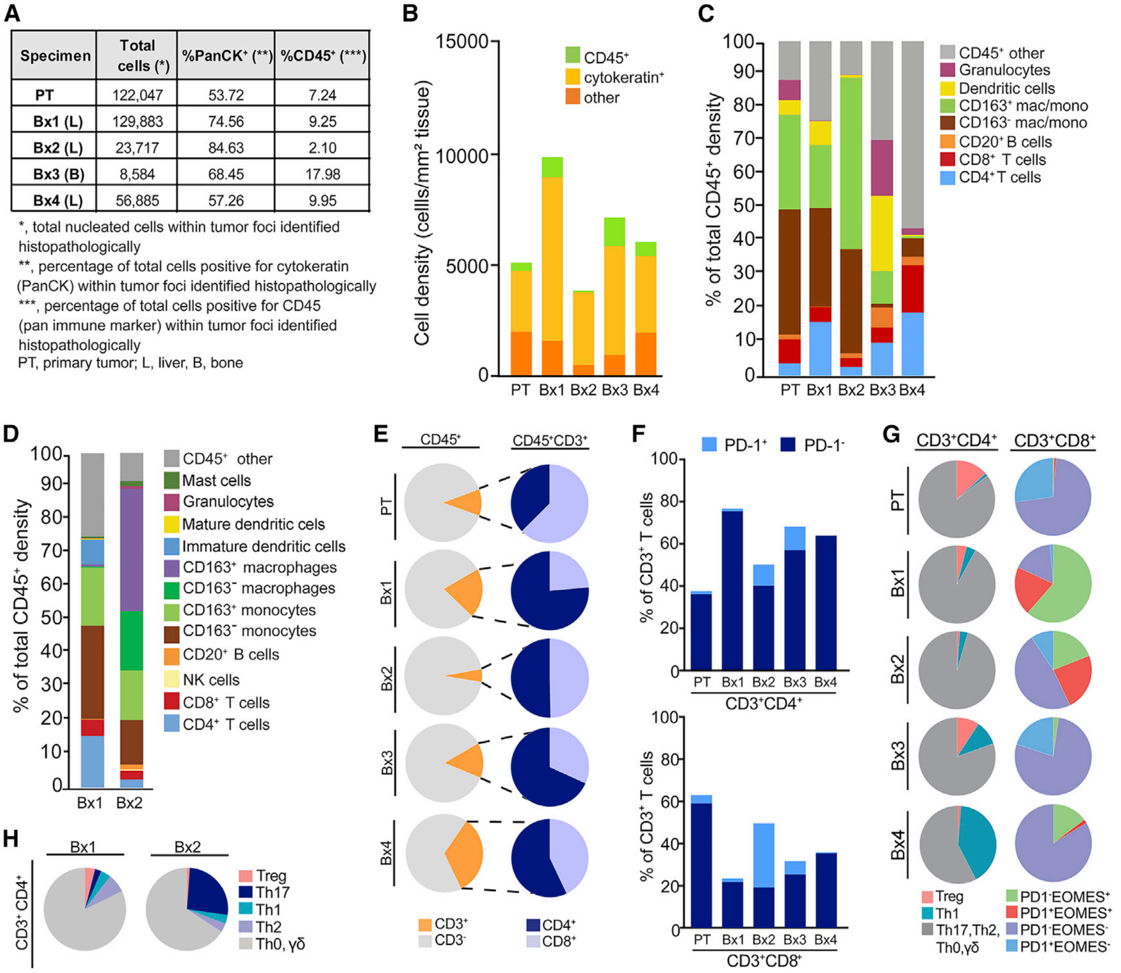

Figure 4. Monitoring response to therapy with deep *in situ* immune phenotyping by mIHC

(A) Primary tumor (PT) and Bx1–Bx4 were subjected to multiplex immunohistochemistry (mIHC) analyses measuring immune ($CD45^+$) and epithelial ($PanCK^+$) cells in tumor compartments as a percentage of total nucleated cells.

(B) Representation of tissue composition, showing density (number of cells per square millimeter of tissue analyzed) of $PanCK^+$ (cytokeratin), $CD45^+$, and $PanCK^-$ $CD45^-$ (other) nucleated cells.

(C) Immune composition of seven major leukocyte lineages, as a percentage of total $CD45^+$ cells.

(D) Deeper auditing of leukocyte lineages in Bx1 and Bx2, measuring 12 immune cell populations and functional states.

(E) $CD3^+$ T cell proportions of total $CD45^+$ cell populations (orange, left), and $CD4^+$ (blue) and $CD8^+$ T cells (periwinkle) proportions within $CD45^+CD3^+$ T cells (right).

(F) PD-1^+ cells as a percentage of total $CD3^+$T cells in the $CD3^+CD4^+$ (top) and $CD3^+CD8^+$ (bottom) T cell populations.

(G) Differentiation state of $CD3^+CD4^+$ T cells, reflected by regulatory T (Treg), Th1, and Th2, Th17, and Th0/$\gamma\delta$ subsets (left) and $CD3^+CD8^+$ T cells, as reflected by expression of PD-1 and EOMES.

(H) Differentiation state of $CD3^+CD4^+$ T cells reflected by Treg, Th17, Th1, Th2, and Th0/$\gamma\delta$ subsets in Bx1 and Bx2.

See also Figure S4 and Table S2.

Figure 4F).[33] However, T cell status was markedly altered in Bx2 (Figure 4C; Table S2). Notably, although T cells were least abundant in Bx2 compared with Bx1 and Bx4, the largest fraction of $CD3^+CD4^+$ and $CD3^+CD8^+$ T cells expressing PD-1 was observed in Bx2 (Bx2: 10.1% $CD4^+$ T cells, 30.5% $CD8^+$ T cells; compared with Bx1: 1.3%, 1.6%; Bx3: 11.3%, 6.3%; Bx4: 0%, 0.7%; Figure 4F), coincident with relatively reduced $FoxP3^+CD4^+$ regulatory T (Treg) cells (Bx1: 4.0%, Bx2: 1.1%, Bx3: 9.3%, Bx4: 0.5%) and expanded Th17 $CD4^+$ T cells (Bx1: 2.5%, Bx2: 26.1%, Bx3: 0%, Bx4: 0%; Figures 4G and 4H).

Analyses of PD-1 and eomesodermin (EOMES) expression showed that the PT contained predominately PD-1^-EOMES$^-$ (71.5%) and PD-1^+EOMES$^-$ $CD8^+$ T cells (27.2%; Figure 4G), likely reflecting naive and early effector subsets, respectively. Evolution of $CD8^+$ T cells in Bx1, Bx2, and Bx4 indicated progressive loss of late effector PD-1^-EOMES$^+$ (61.5%, 19.0%, and 11.0%) and exhausted PD-1^+EOMES$^+$ subsets (20.3%, 23.8%, and 1.7%), with replacement by likely naive PD-1^-EOMES$^-$ $CD8^+$ T cells in Bx1 and Bx2 (16.8%, 47.6%; Figure 4G).

The Bx3 bone metastasis differed from the PT and liver metastases and had the highest percentage of $CD45^+$ leukocytes (18.0%; Figures 4A and 4B) with comparatively high percentages of granulocytes (16.7%; Figure 4C), dendritic cells (22.6%), and $CD20^+$ B cells (6.0%). However, like Bx4, Bx3 contained a prominent granulocyte infiltrate that most likely was predominantly neutrophils (Bx4: 2.0%, Bx3: 16.7%, compare with Bx1: 0.2%, Bx2: 0.1%). Neutrophils can exert significant pro-metastatic activities, including suppressive effects on T cells, and are associated with poor prognosis in many solid tumors, including breast cancer.[34–38]

Tumor and stromal interactions defined using cyclic immunofluorescence (CycIF) and focused ion-beam-scanning electron microscopy (FIB-SEM)

Tumor and stromal compositions and organizations of Bx1–Bx4 plus control biospecimens were assessed using CycIF (Figure 5A).[39,40] This joint analysis revealed 17 tumor and stromal clusters (Figures 5B and S5A–S5E). Three of the stromal clusters (11, 13, and 14) and five of the tumor clusters (0, 1, 5, 7, and 9) comprised major subpopulations in Bx2–Bx4. The three stromal clusters were identified as fibroblast-like cells that differed in levels of vimentin (VIM; cluster 11: 3.2× other clustered means, 13: 2.6×, 14: 4.9×). Endothelial cells (CD31) and macrophages (CD68) were excluded from cluster analysis because of loss of the normal breast and tonsil tissues needed for normalization during staining; their presence was confirmed using manual gating (Figure S5F). All tumor clusters expressed CK7/CK19 but different levels of ER, EGFR, and CK8 (Figure 5B). An additional proliferative cluster, cluster 16, appeared in Bx3 and Bx4, comprised of tumor and stromal cells expressing high levels of Ki67 (31.3×) and/or PCNA (2.4×).

Spatial analyses indicated that tumor cells formed nests surrounded by immune, fibroblast, and endothelial cells as well as collagen I and collagen IV deposits. This was observed in all biopsies but was pronounced in Bx3. Quantitative analyses of nuclear ER and PCNA expression in Bx2–Bx4 as a function of distance to collagen I-rich tumor nest boundaries showed that cells expressing higher levels of ER and PCNA were closest to these boundaries and other stromal compositions (Bx2: mean ER intensity at 0–25 μm from collagen I = 780, 50–75 μm = 463, $p < 0.001$; Bx3: 0–25 μm = 1,058, 50–75 μm = 600, $p < 0.001$; Bx4: 0–25 μm = 1687, 50–75 μm = 1,105, $p < 0.001$. Bx2: mean PCNA intensity at 0–25 μm = 745, 50–75 μm = 218, $p = 0.17$; Bx3: 0–25 μm = 948, 50–75 μm = 567, $p < 0.001$; Bx4: 0–25 μm = 713, 50–75 μm = 406, $p < 0.001$; Figures 5C and S5G); p values describe differences in mean intensities between distances (ANOVA).

Tumor-tumor and tumor-stromal interactions in Bx1, Bx2, and Bx4 were explored at ~4-nm resolution using FIB-SEM.[41] Computational renderings of 3D images of Bx1 (Videos S1 and S2) and Bx2 (Video S3) revealed a previously unappreciated lattice-like structure for fibroblast-like cells surrounding tumor cell clusters and an intricate interaction pattern between these cells, collagen bundles, and tumor cells on the nest boundaries (Figure 5D). The production of collagen by tumor-associated fibroblast-like cells is particularly apparent in the 2D SEM image of Bx4 (Figure 6A).

Intracellular nanobiology defined by FIB-SEM

3D FIB-SEM images of cancer cells in Videos S1, S2, and S3 provide important details about intracellular structures and interactions that may influence cell function and therapeutic response. These include the following. (1) Numerous ~100-nm-diameter, micrometers-long, filopodium-like protrusions (FLPs) and lamellipodia that project from tumor cells into the stromal environment (Figures 6B–6D; Video S3). Published work and our studies in model systems show that these protrusions have actin-rich cores and are decorated with receptor tyrosine kinases that are transported along FLPs by the actin-motor protein Myosin-X.[42] Cultured SKBR3 breast cancer cells exhibit similar FLPs, and dynamic *in vitro* images acquired using stochastic optical reconstruction microscopy (STORM)[43] reveal that the FLPs respond to epidermal growth factor by rapidly decreasing in length, causing cell movement toward the anchored ends of the FLPs (Figure S6B; Video S4). (2) Alignment of mitochondria along the length of an elongated cell and insinuation into nuclear folds (Video S3; Figures 6B and 6E). (3) A high abundance of lamellipodia and macropinosomes, implicating nutrient scavenging via macropinocytosis as a possible tumor survival mechanism (Videos S1 and S3; Figures 6C, 6F, and 6G).[44,45] (4) A high prevalence of densely stained vesicles that appear to be lysosomes (Videos S1 and S3; Figures 6A and 6G).

Figure 6H presents a qualitative summary of the nanoscale features described in Bx1 and Bx2, made by visual analysis of large-format 2D SEM images (Figure S6A) and informed by 3D FIB-SEM images of selected features.

DISCUSSION

This OMS atlas is a compendium of cellular, molecular, and organizational features of four biopsies along with detailed clinical response data collected over a 3.5-year period from a single individual with metastatic breast cancer. It is intended to illustrate the feasibility of generating longitudinal multiplatform analyses in the clinical setting to support investigations of mechanisms of response and resistance beyond those that are apparent from routine omics analyses. Its features include DNA, RNA, and protein[19–21,23] profiles and spatially defined analyses, including mIHC,[27–29] CycIF,[39,40] and 2D and 3D electron microscopy.[41] Other key components are (1) preservation of samples starting within 2 min of biopsy to conserve labile molecular and architectural features, (2) precise temporal linking of clinical and molecular responses with drug treatments and doses, (3) quantitative assessment of individual lesion changes by CT imaging to measure response heterogeneity, (4) workflows that enable multiplatform measurements using material from a single biopsy, and (5) well-curated data and data standards developed by the HTAN program to facilitate community analyses and integration with other datasets.

Selected analyses encompassing multiple OMS atlas datasets illustrate approaches to uncovering mechanisms of drug resistance and response that arise over the course of treatment and that could be missed by limiting analyses to one or a few analytical platforms (Figure 7; Table S3; discussed below). It is important to note that the post hoc analyses described here were not used to guide treatment. They are not meant to definitively

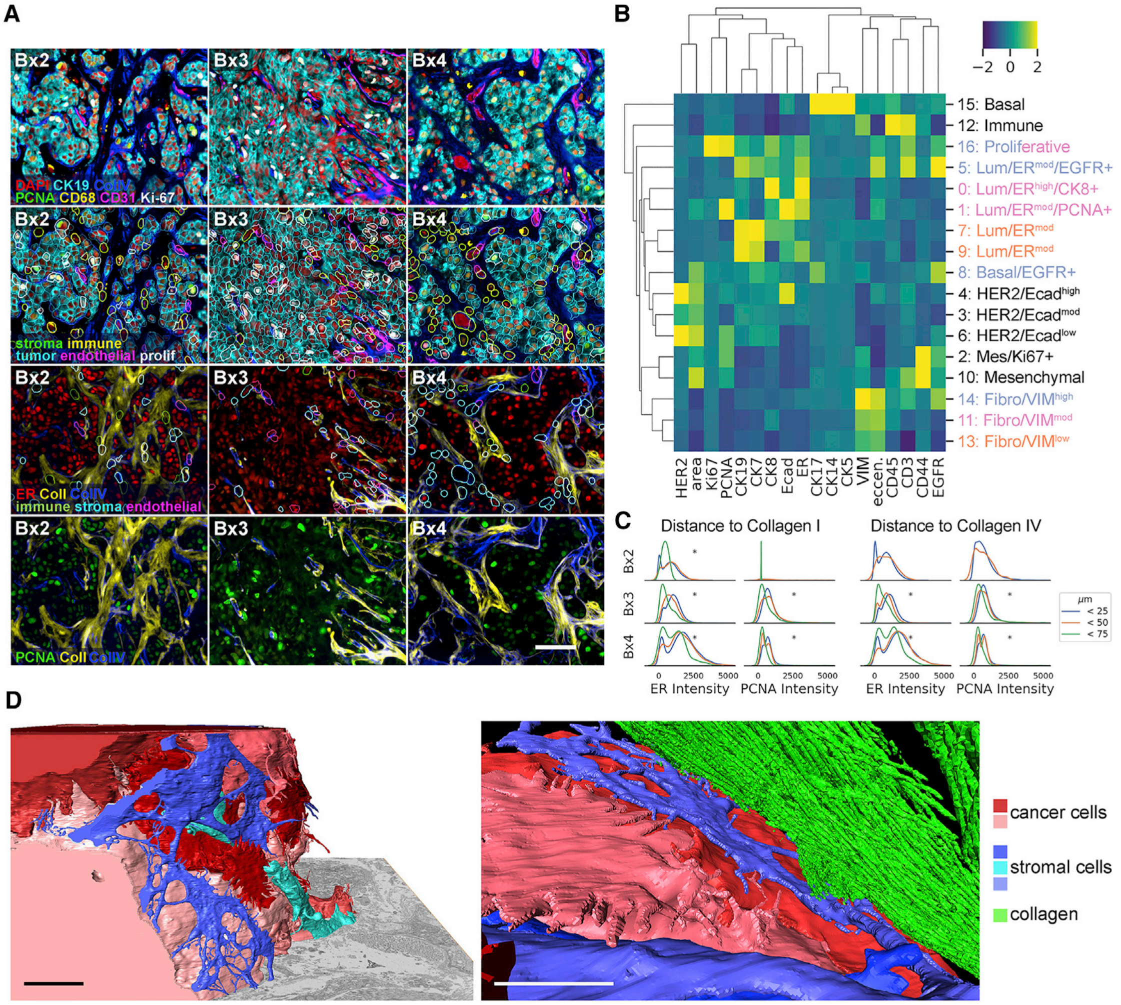

Figure 5. Monitoring tumor and stromal responses to therapy using CycIF and FIB-SEM

(A) Example images of antibody staining overlaid with segmentation borders, colored by cell type. Scale bar, 50 μm.

(B) Heatmap of mean *Z*-scored intensity of unsupervised Leiden clustering (resolution, 0.45) on single-cell mean intensity of biopsies and control tissues and cell lines, with annotations on the right. Lum, luminal; Mes, mesenchymal; Fibro, fibroblast. Colored row labels indicate which biopsy was most dominant for each cluster: Bx2 (orange), Bx3 (blue), or Bx4 (pink). Cluster 16 is evenly split between Bx3 and Bx4.

(C) Single-cell mean intensity distributions of ER and PCNA staining of cells 0–25, 25–50, and 50–75 μm from positive collagen staining. Asterisks indicate significant ($p < 0.001$) differences in mean intensity between distances (ANOVA).

(D) Two views of reconstructed 3D FIB-SEM data from Bx1 showing the relationship between cancer cells (red and pink), stromal cells (blue and turquoise), and collagen (green). A full-volume view (left) shows nanoscale cell-cell interactions of stromal cells surrounding a tumor nest (collagen is not rendered in this image), whereas the close-up view (right) shows a fibroblast-like cell interposed between the tumor and collagen. Scale bars, 5 μm.

See also Figure S5 and Table S5.

explain why the individual progressed on any given therapy but are intended to stimulate validation in follow-up studies.

Phase 1 treatment consisted of a combination of fulvestrant, palbociclib, and everolimus, supported by findings in Bx1 of high ER protein expression, wild-type *ESR1*, and two intact copies of wild-type *RB1*. Bx2 was taken when the tumor began to progress on this treatment (Figure 2). Interestingly, none of the four biopsies analyzed from this individual had mutations in *ESR1* or loss of expression of ER protein (Figure 3E; Table S1) even though *ESR1* mutations are frequently observed in individuals progressing on endocrine therapies.[46] Mutations in *PIK3CA, ERBB2*, and *NF1* also are observed in individuals progressing on endocrine therapies, and *RB1* is frequently lost after treatment with CDK4/6 inhibitors,[47,48] but only a *PIK3CA* mutation and immune-related pathway activation was seen in Bx2 (Figure 3; Table S3). Thus, we interrogated Bx2 data to identify additional bypass mechanisms. One known mechanism by which cells become resistant to everolimus and other mTORC1 inhibitors is through activation of mTORC2.[49,50] Consistent with this, phosphoprotein analyses of Bx2 revealed decreased S6 phosphorylation, which supports continued inhibition of mTORC1 by everolimus (Figure 3E). Concurrently, Bx2 had increased

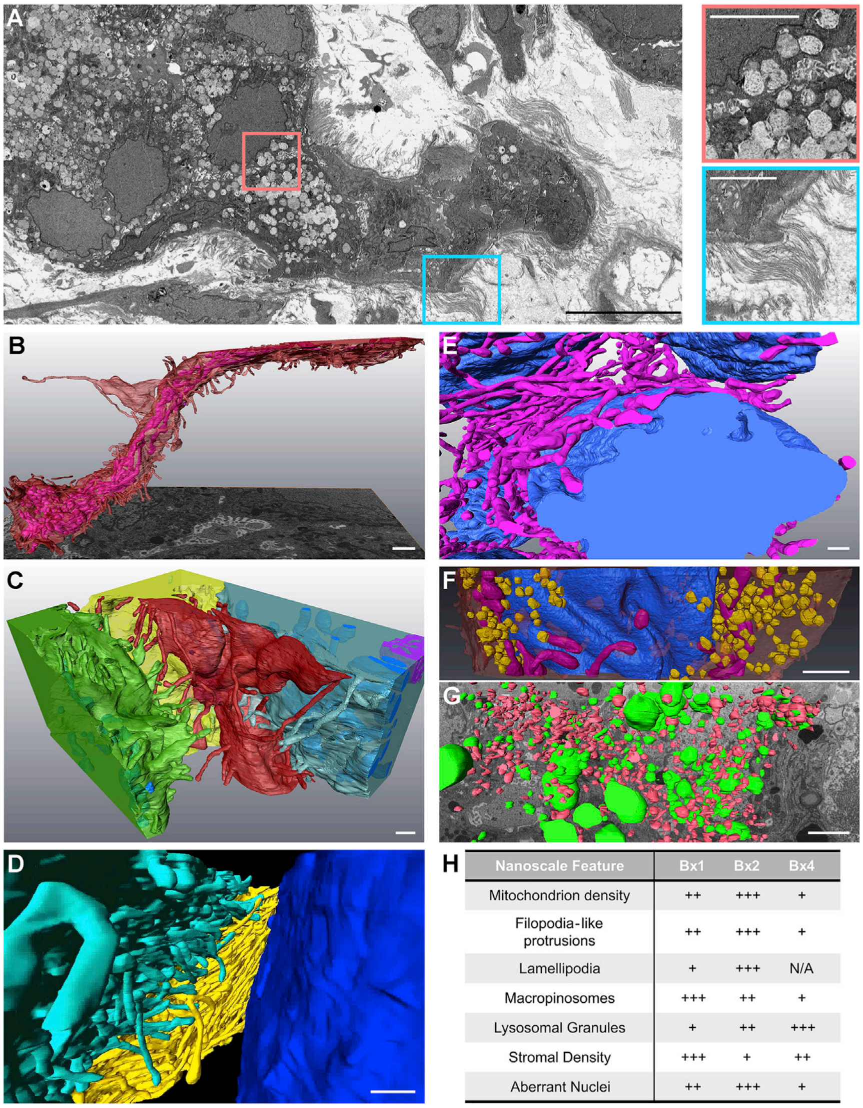

H

Nanoscale Feature	Bx1	Bx2	Bx4
Mitochondrion density	++	+++	+
Filopodia-like protrusions	++	+++	+
Lamellipodia	+	+++	N/A
Macropinosomes	+++	++	+
Lysosomal Granules	+	++	+++
Stromal Density	+++	+	++
Aberrant Nuclei	++	+++	+

Figure 6. Inter- and intracellular compositions and interactions revealed using FIB-SEM

(A) 2D SEM image from Bx4 showing the relationship between tumor cell nests and stromal collagen, along with a high density of extracted lysosomes. Scale bar, 10 μm. The selected insets show these features at high magnification. Scale bars, 3 μm.

(B) A side view of an elongated tumor cell from 3D FIB-SEM of Bx2 showing FLPs (red) and alignment of the internal mitochondria (fuchsia). Scale bar, 1 μm.

(C) Additional cells from Bx2 (the same red cell as in B) showing paddle-shaped lamellipodia (green cell) and long FLPs (red and blue cells) extending into the stroma and interacting with neighboring cells. Scale bar, 500 nm.

(D) Reconstructed 3D FIB-SEM data from Bx1 showing FLPs selectively extending toward neighboring cells and extracellular debris. Scale bar, 1 μm.

(E and F) Additional detail from Bx2 (E) and Bx1 (F) of the nuclear invaginations (blue), showing the organization of mitochondria (fuchsia) and macropinosomes (yellow) with respect to nuclear folds. Scale bars, 1 μm.

(G) 3D FIB-SEM volume of Bx2 showing large electron-dense lysosomal granules (green) dispersed between macropinosomes (red). Scale bar, 900 nm.

(H) Qualitative summary of ultrastructural feature prevalence within each biopsy. Bx4 scoring of lamellipodia is not available.

See also Figure S6 and Videos S1, S2, S3, and S4.

phosphorylation at an mTORC2 site on AKT (S473) and of multiple AKT substrates that together are predicted to maintain oncogenic PI3K/mTOR signaling in the presence of everolimus (Figures 3E and 7A). Everolimus efficacy might also have been reduced by the *PIK3CA* p.E542K activating mutation unique to Bx2, which is known to activate the PI3K/AKT/mTOR pathway.[51] Indeed, this variant was among the SNVs monitored in serial blood samples by DIDA-seq (Figure S2E) and was only detected in ctDNA significantly above background after 7 months on phase 1 therapy (0.06% VAF, p = 0.0071, Weitzman overlapping coefficient), indicating that this mutation may have emerged because of selective pressure from one or more phase 1 drugs.

Several analyses inform on mechanisms of resistance to the CDK4/6 inhibitor palbociclib in Bx2. Loss of *RB1* has been shown to drive resistance in multiple clinical trials,[48] but this gene was not mutated or deleted in Bx2. It is noteworthy that RB1 phosphorylation was at pre-treatment levels in this biopsy (P-S807/S811: 1.0× versus Bx1) because this modification promotes cell cycle progression and should have been decreased by palbociclib.[52] Evidence from protein profiling of key cell cycle regulators revealed that RB1 might have been phosphorylated by CDK2, which also inhibits RB1 but is not a target of palbociclib (Figure 7B).[53–55] First, protein levels of the CDK2 inhibitor p21 were decreased 2× from Bx1 to Bx2 (Figure 3E), possibly because of activated PI3K/AKT signaling, which maintains low p21 levels in CDK4/6 inhibitor-resistant cells.[55] Second, tumor cells with high CCND1 and activated PI3K can adapt to palbociclib via non-canonical binding of CDK2 to CCND1,[53] and Bx2 had higher CCND1 protein levels (1.7× versus Bx1) and PI3K/AKT signaling than Bx1. The CDK4/6 inhibitor abemaciclib has a broader spectrum of activity that includes CDK2[56] and might be expected to be effective in cases where palbociclib escape occurs via CDK2 activation. Indeed, abemaciclib administered subsequent to the period covered by this study showed efficacy (data not shown).

Capecitabine administered in phase 3 along with pembrolizumab, enzalutamide, and fulvestrant initially resulted in a partial response (PR), followed by PD (Figure 2B) at the time of Bx3. Analysis of Bx3 revealed a focal amplification of *TYMS* and *YES1*. TYMS is inhibited by capecitabine, and its overexpression

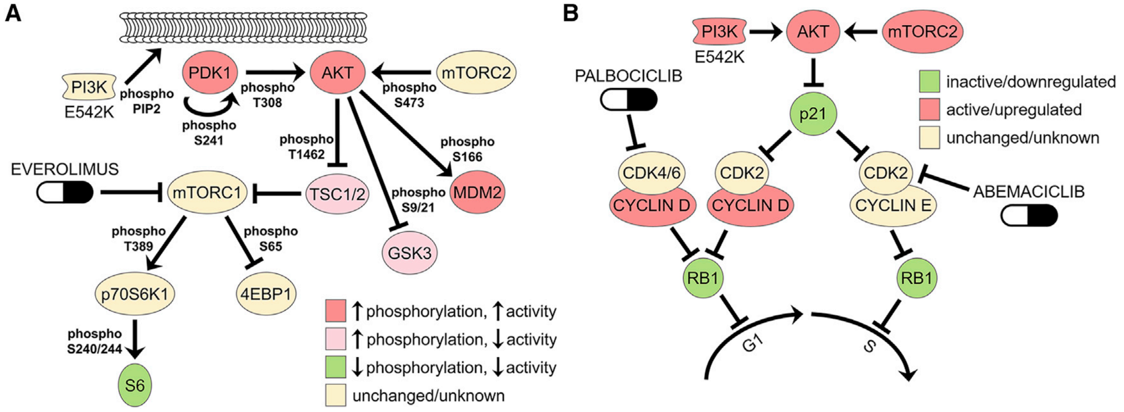

Figure 7. Mechanisms of therapeutic resistance and response suggested by RPPA

(A) Phosphorylation and inferred activation of the PI3K/AKT/mTOR pathway affected by everolimus in Bx2. Decreased phosphorylation of S6 downstream of mTORC1 likely resulted from everolimus inhibition, but increased phosphorylation of proteins downstream of PI3K and AKT, possibly through mutant PI3K E542K activity and/or feedback signaling to mTORC2, may have provided continued oncogenic signaling in the presence of this drug. Proteins are noted as increased activating phosphorylation (>1.4×, red), increased inhibitory phosphorylation (>1.4×, pink), decreased activating phosphorylation (<0.7×, green), or unchanged/unknown phosphorylation (yellow). Changes in phosphorylation in Bx2 versus Bx1: PDK1 = 1.45×, AKT T308 = 1.20×, AKT S473 = 2.69×, TSC2 = 1.43×, GSK3A/B = 1.71×, MDM2 = 1.75×, p70S6K1 = 0.92×, 4EBP1 = 1.37×, S6 S235/236 = 0.69×, S240/244 = 0.14×.

(B) Activation status for cell cycle regulatory pathways affected by palbociclib in Bx2, as inferred from total and phosphoprotein levels. Palbociclib blocks cell division in responsive cells by inhibiting CDK4/6 phosphorylation of RB1, but Bx2 had continued high levels of phospho-RB1 and cell proliferation under treatment with this drug (RB1 P-S807/811 = 0.98× versus pre-treatment Bx1). This is possibly due to degradation of the CDK2 inhibitor p21 (0.50× versus Bx1) by activated PI3K/AKT signaling (see A), which would activate canonical cyclin E/CDK2 complexes to drive cells through G1-S. Alternatively, cell division might be proceeding through the formation of non-canonical cyclin D1/CDK2 complexes because of amplified *CCND1* (Figure S3B), high levels of cyclin D1 protein (1.67× versus Bx1), and low p21. CDK2 activation can be countered with the broad-spectrum CDK inhibitor abemaciclib. Inferred activation status is based on total protein levels or phosphorylation and is designated as relative increases (red), decreases (green), or unchanged/unknown (yellow).

See also Figure 3E and Table S2.

confers resistance to capecitabine.[57] Consequently, *TYMS* amplification may have provided a relative fitness advantage during capecitabine treatment and might explain the temporally late emergence of a clone that branched off early in the evolutionary process (Figure 3B). The *TYMS/YES1* amplicon arose independently in Bx4, presumably because of the earlier capecitabine exposure. But although only *TYMS* was overexpressed in Bx3, both genes were increased in Bx4, indicating that *YES1* may have provided a growth advantage to later lesions after cessation of capecitabine (Figure 2A). YES1 is an SRC family tyrosine kinase and a target of the broad-spectrum kinase inhibitor dasatinib, so inhibition of YES1 might be considered as a possible orthogonal therapeutic strategy for individuals who become resistant to capecitabine via amplification of *TYMS/YES1*. However, dasatinib was administered subsequent to the period covered by this study and did not show efficacy (data not shown), arguing against this strategy.

Comparative analyses of the PT and serial biopsies suggested several mechanisms shaping immune contexture. The most significant was associated with palbociclib treatment at the time of Bx2. mIHC analyses showed increased macrophages/monocytes and T cells and decreased Tregs in Bx2 compared with Bx1 and Bx4 (Figure 4). Th17 cells and Treg cells arise from a common precursor but have opposing functionality upon terminal differentiation, with anti-tumor immunity promoted by Th17 cells and dampened by Treg cells.[58] This suggests that the relatively high frequency of Treg cells in the PT and Bx1 may have contributed to reduced T cell activation, as detected by a lack of PD-1 expression (Figure 4G). Conversely, the Th17 dominance over Treg cells in Bx2 may have supported T cell activation, as evidenced by increased PD-1 expression. These changes were coincident with increases in signaling by IFNγ, ILs, and STATs, as revealed by gene and protein expression profiles (Figure 3C; Table S2), and are consistent with studies in mammary tumor models showing that CDK4/6 inhibitors promote T cell-mediated tumor cell clearance by stimulating type III interferons and suppressing Treg cell proliferation.[59] The utility of an immune checkpoint inhibitor was supported by our observations relating to Bx2 and increased PD-1 expression in T cells. Phase 2 and 3 pembrolizumab treatments were associated with a decrease in the Bx1 and Bx2 lesions (Figure 2C), but the role of pembrolizumab in the decrease in lesion size is unknown as it was given with other drugs. Indeed, a challenge in this and other studies is in deconvoluting effects of individual agents when given in combination. Pembrolizumab and other phase 3 drugs were discontinued upon PD, whereupon the immune contexture changed again, with Bx4 showing more T cells but fewer macrophages/monocytes and Th17 T cells. Although Bx4 also contained fewer Treg cells (similar to Bx2) and the highest proportions of Th1 differentiation (Figure 4G), there was low PD-1 expression on T cells (Figure 4F). So, although the PT, Bx1, and Bx4 showed minimal T cell responses, this may have been due to low neoantigens and antigen presentation as likely barriers to functional anti-tumor immunity in Bx4, whereas T cell-mediated suppression was predominant in the PT and Bx1.

CycIF and FIB-SEM analyses showed tumor cells organized into nests surrounded by stromal cells and substantial collagen I deposits (Figures 5 and 6), suggesting that the lack of neoantigens and/or antigen presentation inferred from immune profiling may be caused, at least in part, by biophysical barriers that diminish tumor-immune cell interactions. Interestingly, the 3D images suggest that fibroblast-like cells are interposed between tumor cells and collagen bundles in most cases, raising the issue of how collagen stiffening leads to more aggressive tumor behavior[60] and how stromal barriers stimulate increased expression of ER and PCNA in closely proximal tumor cells (Figure 5D). From a technical perspective, the complex cellular interactions revealed by FIB-SEM illustrate the difficulties of properly segmenting individual cells during multiplex imaging of 2D sections using mIHC or CycIF (Figure S6C) and of dissociating tightly interacting and potentially fragile cells for single-cell analyses.

The FIB-SEM analyses reveal several ultrastructural features that may influence tumor behavior and/or therapeutic vulnerability. These include the following. (1) FLPs that project from tumor cells into the stromal microenvironment. The receptor-dense, dynamic nature of FLPs may mediate proximal and distal interactions with elements of the microenvironment and enable directed movement therein. This might provide the force needed to produce the elongated tumor cell shown in Figure 6B, with mitochondria aligned along its long axis and inserted into nuclear folds (Figure 6E). FLPs have also been implicated in protein transport between cells.[61] These functions suggest the possible utility of FLP inhibitors. (2) Insinuation of mitochondria into nuclear folds (Video S3; Figures 6B and 6E). These apparently forced interactions may increase the potential for nucleus-mitochondrion signaling that would alter DNA damage repair and/or reactive oxygen species (ROS) signaling.[62,63] This might be countered therapeutically by attacking ROS or by inhibiting FLP function. (3) High abundance of lamellipodia and macropinosomes (Videos S1 and S3; Figures 6C, 6F, and 6G). Nutrient scavenging from the intercellular space and nearby dying cells is a known tumor survival mechanism.[44,45,64] Protein-conjugated drugs might convert this survival mechanism into a therapeutic vulnerability. We speculate that this mechanism may have been partly responsible for the control achieved by treatment with liposomal doxorubicin during phase 3. Macropinocytosis may also diminish communication of neoantigens to immune cells by competing with dendritic cells for exogenous antigens released from dying tumor cells.[65] (4) High-density, densely stained vesicles that appear to be lysosomes (Videos S1 and S3; Figures 6A and 6G). Lysosomes can sequester cancer drugs via a process called lysosomotropism, in which weakly basic drugs become protonated and trapped within the acidic interior of lysosomes.[66] Lysosomotropic sequestration has been implicated as a mechanism of resistance to CDK4/6 inhibitors[67,68] and is suggested in this individual by the increase in lysosomes from Bx1 to Bx2, as seen by FIB-SEM (Figures 6G, 6H, and S6A; Videos S1 and S3). Interestingly, hydroxychloroquine, sometimes used to counter treatment-induced rashes, has been reported to be lysosomotropic and thus might reduce treatment efficacy when co-administered with any basic drug, including CDK4/6 inhibitors.[69] Recent studies suggest that lysosomotropism-mediated doxorubicin resistance can be countered by the β-AR antagonist propranolol, which acts through a β-AR-independent mechanism to increase cytoplasmic doxorubicin concentrations and decrease lysosomal accumulation.[70]

Overall, this OMS atlas shows the promises and challenges of elucidating evolving resistance mechanisms and new therapeutic vulnerabilities in individuals. The study shows that multi-analyte workflows can be executed routinely and safely. Analyses of the data provide insights into mechanisms of tumor response and resistance that can be explored in subsequent studies. The ready availability of the data and protocols in standardized form will encourage further analyses and method development.

Limitations of the study

The overall goal of this study is to elucidate the mechanisms of resistance and therapeutic vulnerability experienced by an individual during extended treatment of metastatic disease, using information from multiple analytical workflows. We acknowledge the difficulty of assigning specific response and resistance mechanisms to individual drugs within a multi-drug treatment regimen, especially for drug combinations targeting overlapping biological pathways, and we note the danger of "cherry picking" mechanisms from the vast published literature. Moreover, working with a single human subject precludes implementation of hypothesis testing, which is *de rigueur* in experimental cell lines, animal models, and clinical studies that would more definitively support our proposed mechanisms. These factors demonstrate the challenges of implementing this type of program and analyzing N-of-1 data in a real-world clinical setting. However, our studies do suggest mechanisms and interpretational processes that can be tested in larger studies.

Several of our methods are too complex to be widely applied as currently implemented. However, when the utility of each assay platform is established, workflows can be simplified and streamlined. Our work shows that further development of analytical methods to integrate and interpret multi-platform omics and imaging datasets is clearly needed for the clinical and research communities. The OMS atlas can serve as a resource in that effort.

Finally, we are aware that we are inferring mechanisms from single biopsies of a metastatic disease that displays remarkable intra- and intermetastatic lesion heterogeneity.[71] A biopsy of a single metastatic lesion at any single time point is unlikely to provide a comprehensive picture of the entire heterogeneous disease within an individuals or even within the biopsied lesion. This is a fundamental limitation of any biopsy-based analytical strategy. Continued advancement of assays that report on overall tumor composition across multiple lesions, such as peripheral blood assays, is one potential avenue toward understanding heterogeneous disease burden. Indeed, our observation that radiation induced a transient increase in ctDNA in peripheral blood suggests that individuals undergoing radiotherapy might have circulating tumor nucleic acids and proteins in sufficient quantities for practical diagnostic measurement and for revealing latent, low-level molecular changes in unbiopsied lesions in almost real time.

STAR★METHODS

Detailed methods are provided in the online version of this paper and include the following:

- KEY RESOURCES TABLE
- RESOURCE AVAILABILITY
  - Lead contact
  - Materials availability
  - Data and code availability
- EXPERIMENTAL MODEL AND SUBJECT DETAILS
  - Human subjects
  - Cell lines
- METHOD DETAILS
  - Clinical decision making
  - Radiology
  - Clinical and exploratory workflows
  - GeneTrails® solid tumor panel
  - Blood collection and DNA isolation
  - Whole exome sequencing
  - Dual index degenerate adaptor sequencing
  - Low-pass whole genome sequencing
  - Whole transcriptome sequencing
  - Reverse phase protein arrays
  - Intracellular signaling protein panel
  - Multiplex immunohistochemistry
  - Cyclic immunofluorescence
  - Scanning electron microscopy
  - Stochastic optical reconstruction microscopy
- QUANTIFICATION AND STATISTICAL ANALYSIS
  - Radiology
  - Whole exome sequencing
  - Dual index degenerate adaptor sequencing
  - Low-pass whole genome sequencing
  - Whole transcriptome sequencing
  - Transcriptional regulator networks
  - Reverse phase protein arrays
  - Intracellular signaling protein panel
  - Integrative analyses
  - Multiplex immunohistochemistry
  - Cyclic immunofluorescence
- ADDITIONAL RESOURCES

SUPPLEMENTAL INFORMATION

Supplemental information can be found online at https://doi.org/10.1016/j.xcrm.2022.100525.

ACKNOWLEDGMENTS

This publication is part of the HTAN (Human Tumor Atlas Network) Consortium paper package. A list of HTAN members is available at humantumoratlas.org/htan-authors/.

This project was carried out with major support from the OHSU SMMART Program, National Institutes of Health (NIH), National Cancer Institute (NCI) Human Tumor Atlas Network (HTAN) Research Center (U2CCA233280), and Prospect Creek Foundation. The program was initiated with support from a Stand Up to Cancer-American Association for Cancer Research Dream Team Translational Cancer Research Grant, SU2C-AACR-DT0409. Additional support came from the OHSU Brenden-Colson Center for Pancreatic Care, the W.M. Keck Foundation, NIH/NCI Cancer Target Discovery and Development (CTD^2) (U01CA217842), the NIH/NCI Cancer Systems Biology Consortium Center (U54CA209988), NIH/NCI U01CA224012 (to L.M.C.), NIH SBIR (R44CA224994) (to K.C.), the Damon Runyon Cancer Research Foundation (to X.N.), and the M.J. Murdock Charitable Trust. Sequencing and multiscale microscopy were supported by a Knight Cancer Institute Cancer Center Support grant (5P30CA69533). Electron microscopy was performed at the Multiscale Microscopy Core; light microscopy was performed using equipment in the Advanced Light Microscopy Core; both are OHSU University Shared Resource Cores. Short read sequencing assays were performed by the OHSU Massively Parallel Sequencing Shared Resource. We acknowledge assistance from the following team members: Taylor Kelley, Marlana Klinger, Max Morris, Anastasiya Olson, Kiara Siex, and Leanna Williams (research operations); Imogen Bentley, Georgia Mayfield, and Matt Viehdorfer (information management); Özgün Babur (omics); Teresa Beechwood, Konjit Betre, Gina Choe, Giovanney Gonzalez, Nell Kirchberger, and Lauren Maloney (mIHC); Steven Adamou, Dylan Blumberg, Cecilia Bueno, Kaylyn Devlin, Yingsi Gao, David Kilburn, Moqing Liu, Kevin Loftis, Hannah Smith, Rebecca Smith, Kevin Stoltz, and Erin S. Stempinski (FIB-SEM); and Jing Wang (STORM).

AUTHOR CONTRIBUTIONS

Conceptualization, R.B., G.B.M., and J.W.G.; methodology, B.E.J., J.M.K., S.P., A.K., K.C., X.N., L.M.H., P.T.S., E.D., L.M.C., C.C., J.G., and J.W.G.; software, A.L.C., M.B.H., P.L., S.S., D.S., G. Thibault, E.D., and Y.H.C.; formal analysis, A.L.C., A.B., C.B., E. Bucher, E. Burlingame, J. Eng, J. Estabrook, M.L., J.L., J.P., S.S., J.S., G. Thibault, R.F.T., and B.R.W.; investigation, B.E.J., J.M.K., B.L.K., S.P., C.B.B., C.B., T.C., K.C., Z.H., J. Eng, J.L.R., X.N., G. Thomas, A.R.G., and C.C.; resources, R.B. and Z.M.; data curation, A.L.C., J.M.K., S.P., S.M., and C.Z.; writing—original draft, B.E.J., A.L.C., J.M.S., J.M.K., S.P., C.B.B., K.C., J.L.R., J.G., and J.W.G.; writing—review & editing, B.E.J., A.L.C., J.M.S., J.M.K., C.B.B., A.B., K.C., A.K., B.L.K., M.L., J.L.R., L.M.C., J.G., R.B., Z.M., G.B.M., and J.W.G.; visualization, A.L.C., J.M.S., S.P., C.B.B., E. Burlingame, K.C., J. Eng, M.L., J.P., J.L.R., and X.N.; supervision, C.C., J.G., R.B., Z.M., G.B.M., and J.W.G.; project administration, B.E.J., S.P., H.S.F., and A.K.; funding acquisition, L.M.C., C.C., J.G., R.B., G.B.M., and J.W.G.

DECLARATION OF INTERESTS

D.S. is employed by Quantitative Imaging Systems. L.M.C. is a paid consultant for Cell Signaling Technologies, Shasqi, and AbbVie; received reagent and/or research support from Plexxikon, Pharmacyclics, Acerta Pharma, Deciphera Pharmaceuticals, Genentech, Roche Glycart AG, Syndax Pharmaceuticals, Innate Pharma, and NanoString Technologies; and is a member of the scientific advisory boards of Syndax Pharmaceuticals, Carisma Therapeutics, Zymeworks, Verseau Therapeutics, Cytomix Therapeutics, and Kineta. G.B.M. has licensed technologies to Myriad Genetics and NanoString; is on the SAB or is a consultant to Amphista, AstraZeneca, Chrysallis Biotechnology, GSK, ImmunoMET, Ionis, Lilly, PDX Pharmaceuticals, Signalchem Lifesciences, Symphogen, Tarveda, Turbine, and Zentalis Pharmaceuticals; and has stock/options/financial interests in Catena Pharmaceuticals, ImmunoMet, SignalChem, and Tarveda. J.W.G. has licensed technologies to Abbott Diagnostics, Zorro Bio, and PDX Pharmaceuticals; has ownership positions in Convergent Genomics, Health Technology Innovations, Zorro Bio, and PDX Pharmaceuticals; serves as a paid consultant to New Leaf Ventures; has received research support from Thermo Fisher Scientific (formerly FEI), Zeiss, Miltenyi Biotech, Cepheid (Danaher), Quantitative Imaging, Health Technology Innovations, and Micron Technologies; and owns stock in Abbott Diagnostics, AbbVie, Alphabet, Amazon, Amgen, Apple, General Electric, Gilead, Intel, Microsoft, Nvidia, and Zimmer Biomet.

Received: July 7, 2021
Revised: November 15, 2021
Accepted: January 19, 2022
Published: February 15, 2022

REFERENCES

1. Von Hoff, D.D., Stephenson, J.J., Jr., Rosen, P., Loesch, D.M., Borad, M.J., Anthony, S., Jameson, G., Brown, S., Cantafio, N., Richards, D.A., et al. (2010). Pilot study using molecular profiling of patients' tumors to find potential targets and select treatments for their refractory cancers. J. Clin. Oncol. *28*, 4877–4883.
2. Schwaederle, M., Zhao, M., Lee, J.J., Eggermont, A.M., Schilsky, R.L., Mendelsohn, J., Lazar, V., and Kurzrock, R. (2015). Impact of precision medicine in diverse cancers: a meta-analysis of phase II clinical trials. J. Clin. Oncol. *33*, 3817–3825.
3. Sanchez, N.S., Mills, G.B., and Mills Shaw, K.R. (2017). Precision oncology: neither a silver bullet nor a dream. Pharmacogenomics *18*, 1525–1539.
4. Mitri, Z.I., Parmar, S., Johnson, B., Kolodzie, A., Keck, J.M., Morris, M., Guimaraes, A.R., Beckett, B.R., Borate, U., Lopez, C.D., et al. (2018). Implementing a comprehensive translational oncology platform: from molecular testing to actionability. J. Transl. Med. *16*, 358.
5. Rozenblatt-Rosen, O., Regev, A., Oberdoerffer, P., Nawy, T., Hupalowska, A., Rood, J.E., Ashenberg, O., Cerami, E., Coffey, R.J., Demir, E., et al. (2020). The human tumor atlas network: charting tumor transitions across space and time at single-cell resolution. Cell *181*, 236–249.
6. Paik, S., Shak, S., Tang, G., Kim, C., Baker, J., Cronin, M., Baehner, F.L., Walker, M.G., Watson, D., Park, T., et al. (2004). A multigene assay to predict recurrence of tamoxifen-treated, node-negative breast cancer. N. Engl. J. Med. *351*, 2817–2826.
7. National Comprehensive Cancer Network. Breast cancer (Version 8.2021).https://www.nccn.org/professionals/physician_gls/pdf/breast.pdf.
8. Eisenhauer, E.A., Therasse, P., Bogaerts, J., Schwartz, L.H., Sargent, D., Ford, R., Dancey, J., Arbuck, S., Gwyther, S., Mooney, M., et al. (2009). New response evaluation criteria in solid tumours: revised RECIST guideline (version 1.1). Eur. J. Cancer *45*, 228–247.
9. Dogruluk, T., Tsang, Y.H., Espitia, M., Chen, F., Chen, T., Chong, Z., Appadurai, V., Dogruluk, A., Eterovic, A.K., Bonnen, P.E., et al. (2015). Identification of variant-specific functions of PIK3CA by rapid phenotyping of rare mutations. Cancer Res. *75*, 5341–5354.
10. Ng, P.K., Li, J., Jeong, K.J., Shao, S., Chen, H., Tsang, Y.H., Sengupta, S., Wang, Z., Bhavana, V.H., Tran, R., et al. (2018). Systematic functional annotation of somatic mutations in cancer. Cancer Cell *33*, 450–462.e410.
11. O'Meara, T.A., and Tolaney, S.M. (2021). Tumor mutational burden as a predictor of immunotherapy response in breast cancer. Oncotarget *12*, 394–400.
12. McGranahan, N., Furness, A.J., Rosenthal, R., Ramskov, S., Lyngaa, R., Saini, S.K., Jamal-Hanjani, M., Wilson, G.A., Birkbak, N.J., Hiley, C.T., et al. (2016). Clonal neoantigens elicit T cell immunoreactivity and sensitivity to immune checkpoint blockade. Science *351*, 1463–1469.
13. Blass, E., and Ott, P.A. (2021). Advances in the development of personalized neoantigen-based therapeutic cancer vaccines. Nat. Rev. Clin. Oncol. *18*, 215–229.
14. Butler, T.M., Boniface, C.T., Johnson-Camacho, K., Tabatabaei, S., Melendez, D., Kelley, T., Gray, J., Corless, C.L., and Spellman, P.T. (2019). Circulating tumor DNA dynamics using patient-customized assays are associated with outcome in neoadjuvantly treated breast cancer. Cold Spring Harb. Mol. Case Stud. *5*, a003772.
15. Parker, J.S., Mullins, M., Cheang, M.C., Leung, S., Voduc, D., Vickery, T., Davies, S., Fauron, C., He, X., Hu, Z., et al. (2009). Supervised risk predictor of breast cancer based on intrinsic subtypes. J. Clin. Oncol. *27*, 1160–1167.
16. Cancer Genome Atlas Network (2012). Comprehensive molecular portraits of human breast tumours. Nature *490*, 61–70.
17. Hanzelmann, S., Castelo, R., and Guinney, J. (2013). GSVA: gene set variation analysis for microarray and RNA-seq data. BMC Bioinformatics *14*, *7*.
18. Liberzon, A., Birger, C., Thorvaldsdottir, H., Ghandi, M., Mesirov, J.P., and Tamayo, P. (2015). The Molecular Signatures Database (MSigDB) hallmark gene set collection. Cell Syst. *1*, 417–425.
19. Chen, M.M., Li, J., Wang, Y., Akbani, R., Lu, Y., Mills, G.B., and Liang, H. (2019). TCPA v3.0: an integrative platform to explore the pan-cancer analysis of functional proteomic data. Mol. Cell Proteomics *18*, S15–S25.
20. Labrie, M., Fang, Y., Kendsersky, N.D., Li, J., Liang, H., Westin, S.N., Mitri, Z., and Mills, G.B. (2019). Using reverse phase protein array (RPPA) to identify and target adaptive resistance. Adv. Exp. Med. Biol. *1188*, 251–266.
21. Tibes, R., Qiu, Y., Lu, Y., Hennessy, B., Andreeff, M., Mills, G.B., and Kornblau, S.M. (2006). Reverse phase protein array: validation of a novel proteomic technology and utility for analysis of primary leukemia specimens and hematopoietic stem cells. Mol. Cancer Ther. *5*, 2512–2521.
22. Labrie, M., Kim, T.B., Ju, Z., Lee, S., Zhao, W., Fang, Y., Lu, Y., Chen, K., Ramirez, P., Frumovitz, M., et al. (2019). Adaptive responses in a PARP inhibitor window of opportunity trial illustrate limited functional interlesional heterogeneity and potential combination therapy options. Oncotarget *10*, 3533–3546.
23. Lee, J., Geiss, G.K., Demirkan, G., Vellano, C.P., Filanoski, B., Lu, Y., Ju, Z., Yu, S., Guo, H., Bogatzki, L.Y., et al. (2018). Implementation of a multiplex and quantitative proteomics platform for assessing protein lysates using DNA-barcoded antibodies. Mol. Cell Proteomics *17*, 1245–1258.
24. Cerami, E.G., Gross, B.E., Demir, E., Rodchenkov, I., Babur, O., Anwar, N., Schultz, N., Bader, G.D., and Sander, C. (2011). Pathway Commons, a web resource for biological pathway data. Nucleic Acids Res. *39*, D685–D690.
25. Babur, Ö., Luna, A., Korkut, A., Durupinar, F., Siper, M.C., Dogrusoz, U., Vaca Jacome, A.S., Peckner, R., Christianson, K.E., Jaffe, J.D., et al. (2021). Causal interactions from proteomic profiles: molecular data meet pathway knowledge. Patterns *2*, 100257.
26. Ahmad, R., Rajabi, H., Kosugi, M., Joshi, M.D., Alam, M., Vasir, B., Kawano, T., Kharbanda, S., and Kufe, D. (2011). MUC1-C oncoprotein promotes STAT3 activation in an autoinductive regulatory loop. Sci. Signal. *4*, ra9.
27. Banik, G., Betts, C.B., Liudahl, S.M., Sivagnanam, S., Kawashima, R., Cotechini, T., Larson, W., Goecks, J., Pai, S.I., Clayburgh, D.R., et al. (2020). High-dimensional multiplexed immunohistochemical characterization of immune contexture in human cancers. Methods Enzymol. *635*, 1–20.
28. Tsujikawa, T., Kumar, S., Borkar, R.N., Azimi, V., Thibault, G., Chang, Y.H., Balter, A., Kawashima, R., Choe, G., Sauer, D., et al. (2017). Quantitative multiplex immunohistochemistry reveals myeloid-inflamed tumor-immune complexity associated with poor prognosis. Cell Rep. *19*, 203–217.
29. Tsujikawa, T., Thibault, G., Azimi, V., Sivagnanam, S., Banik, G., Means, C., Kawashima, R., Clayburgh, D.R., Gray, J.W., Coussens, L.M., et al. (2019). Robust cell detection and segmentation for image cytometry reveal Th17 cell heterogeneity. Cytometry A *95*, 389–398.
30. Barros, M.H., Hauck, F., Dreyer, J.H., Kempkes, B., and Niedobitek, G. (2013). Macrophage polarisation: an immunohistochemical approach for identifying M1 and M2 macrophages. PLoS One *8*, e80908.e8.
31. Skytthe, M.K., Graversen, J.H., and Moestrup, S.K. (2020). Targeting of CD163(+) macrophages in inflammatory and Malignant diseases. Int. J. Mol. Sci. *21*, 5497.
32. Buechler, C., Eisinger, K., and Krautbauer, S. (2013). Diagnostic and prognostic potential of the macrophage specific receptor CD163 in inflammatory diseases. Inflamm. Allergy Drug Targets *12*, 391–402.
33. Pauken, K.E., and Wherry, E.J. (2015). Overcoming T cell exhaustion in infection and cancer. Trends Immunol. *36*, 265–276.

34. Albrengues, J., Shields, M.A., Ng, D., Park, C.G., Ambrico, A., Poindexter, M.E., Upadhyay, P., Uyeminami, D.L., Pommier, A., Kuttner, V., et al. (2018). Neutrophil extracellular traps produced during inflammation awaken dormant cancer cells in mice. Science *361*, eaao4227.

35. Park, J., Wysocki, R.W., Amoozgar, Z., Maiorino, L., Fein, M.R., Jorns, J., Schott, A.F., Kinugasa-Katayama, Y., Lee, Y., Won, N.H., et al. (2016). Cancer cells induce metastasis-supporting neutrophil extracellular DNA traps. Sci. Transl Med. *8*, 361ra138.

36. Coffelt, S.B., Wellenstein, M.D., and de Visser, K.E. (2016). Neutrophils in cancer: neutral no more. Nat. Rev. Cancer *16*, 431–446.

37. Ethier, J.L., Desautels, D., Templeton, A., Shah, P.S., and Amir, E. (2017). Prognostic role of neutrophil-to-lymphocyte ratio in breast cancer: a systematic review and meta-analysis. Breast Cancer Res. *19*, 2.

38. Wellenstein, M.D., Coffelt, S.B., Duits, D.E.M., van Miltenburg, M.H., Slagter, M., de Rink, I., Henneman, L., Kas, S.M., Prekovic, S., Hau, C.S., et al. (2019). Loss of p53 triggers WNT-dependent systemic inflammation to drive breast cancer metastasis. Nature *572*, 538–542.

39. Eng, J., Thibault, G., Luoh, S.W., Gray, J.W., Chang, Y.H., and Chin, K. (2020). Cyclic multiplexed-immunofluorescence (cmIF), a highly multiplexed method for single-cell analysis. Methods Mol. Biol. *2055*, 521–562.

40. Lin, J.R., Fallahi-Sichani, M., and Sorger, P.K. (2015). Highly multiplexed imaging of single cells using a high-throughput cyclic immunofluorescence method. Nat. Commun. *6*, 8390.

41. Riesterer, J.L., Lopez, C.S., Stempinski, E.S., Williams, M., Loftis, K., Stoltz, K., Thibault, G., Lanicault, C., Williams, T., and Gray, J.W. (2020). A workflow for visualizing human cancer biopsies using large-format electron microscopy. Methods Cell Biol. *158*, 163–181.

42. Kerber, M.L., and Cheney, R.E. (2011). Myosin-X: a MyTH-FERM myosin at the tips of filopodia. J. Cell Sci *124*, 3733–3741.

43. Creech, M.K., Wang, J., Nan, X., and Gibbs, S.L. (2017). Superresolution imaging of clinical formalin fixed paraffin embedded breast cancer with single molecule localization microscopy. Sci. Rep. *7*, 40766.

44. Commisso, C., Davidson, S.M., Soydaner-Azeloglu, R.G., Parker, S.J., Kamphorst, J.J., Hackett, S., Grabocka, E., Nofal, M., Drebin, J.A., Thompson, C.B., et al. (2013). Macropinocytosis of protein is an amino acid supply route in Ras-transformed cells. Nature *497*, 633–637.

45. Hosios, A.M., Hecht, V.C., Danai, L.V., Johnson, M.O., Rathmell, J.C., Steinhauser, M.L., Manalis, S.R., and Vander Heiden, M.G. (2016). Amino acids rather than glucose account for the majority of cell mass in proliferating mammalian cells. Dev. Cell *36*, 540–549.

46. Razavi, P., Chang, M.T., Xu, G., Bandlamudi, C., Ross, D.S., Vasan, N., Cai, Y., Bielski, C.M., Donoghue, M.T.A., Jonsson, P., et al. (2018). The genomic landscape of endocrine-resistant advanced breast cancers. Cancer Cell *34*, 427–438.e426.

47. Hanker, A.B., Sudhan, D.R., and Arteaga, C.L. (2020). Overcoming endocrine resistance in breast cancer. Cancer Cell *37*, 496–513.

48. McCartney, A., Migliaccio, I., Bonechi, M., Biagioni, C., Romagnoli, D., De Luca, F., Galardi, F., Risi, E., De Santo, I., Benelli, M., et al. (2019). Mechanisms of resistance to CDK4/6 inhibitors: potential implications and biomarkers for clinical practice. Front. Oncol. *9*, 666.

49. Mi, W., Ye, Q., Liu, S., and She, Q.B. (2015). AKT inhibition overcomes rapamycin resistance by enhancing the repressive function of PRAS40 on mTORC1/4E-BP1 axis. Oncotarget *6*, 13962–13977.

50. O'Reilly, K.E., Rojo, F., She, Q.B., Solit, D., Mills, G.B., Smith, D., Lane, H., Hofmann, F., Hicklin, D.J., Ludwig, D.L., et al. (2006). mTOR inhibition induces upstream receptor tyrosine kinase signaling and activates Akt. Cancer Res. *66*, 1500–1508.

51. Kang, S., Bader, A.G., and Vogt, P.K. (2005). Phosphatidylinositol 3-kinase mutations identified in human cancer are oncogenic. Proc. Natl. Acad. Sci. U S A *102*, 802–807.

52. Sherr, C.J., Beach, D., and Shapiro, G.I. (2016). Targeting CDK4 and CDK6: from discovery to therapy. Cancer Discov. *6*, 353–367.

53. Herrera-Abreu, M.T., Palafox, M., Asghar, U., Rivas, M.A., Cutts, R.J., Garcia-Murillas, I., Pearson, A., Guzman, M., Rodriguez, O., Grueso, J., et al. (2016). Early adaptation and acquired resistance to CDK4/6 inhibition in Estrogen receptor-positive breast cancer. Cancer Res. *76*, 2301–2313.

54. Iida, M., Nakamura, M., Tokuda, E., Toyosawa, D., Niwa, T., Ohuchi, N., Ishida, T., and Hayashi, S.I. (2019). The p21 levels have the potential to be a monitoring marker for ribociclib in breast cancer. Oncotarget *10*, 4907–4918.

55. Vilgelm, A.E., Saleh, N., Shattuck-Brandt, R., Riemenschneider, K., Slesur, L., Chen, S.C., Johnson, C.A., Yang, J., Blevins, A., Yan, C., et al. (2019). MDM2 antagonists overcome intrinsic resistance to CDK4/6 inhibition by inducing p21. Sci. Transl. Med. *11*, eaav7171.

56. Hafner, M., Mills, C.E., Subramanian, K., Chen, C., Chung, M., Boswell, S.A., Everley, R.A., Liu, C., Walmsley, C.S., Juric, D., et al. (2019). Multiomics profiling establishes the polypharmacology of FDA-approved CDK4/6 inhibitors and the potential for differential clinical activity. Cell Chem. Biol. *26*, 1067–1080.e1068.

57. Marangoni, E., Laurent, C., Coussy, F., El-Botty, R., Chateau-Joubert, S., Servely, J.L., de Plater, L., Assayag, F., Dahmani, A., Montaudon, E., et al. (2018). Capecitabine efficacy is correlated with TYMP and RB1 expression in PDX established from triple-negative breast cancers. Clin. Cancer Res. *24*, 2605–2615.

58. Knochelmann, H.M., Dwyer, C.J., Bailey, S.R., Amaya, S.M., Elston, D.M., Mazza-McCrann, J.M., and Paulos, C.M. (2018). When worlds collide: Th17 and Treg cells in cancer and autoimmunity. Cell Mol. Immunol. *15*, 458–469.

59. Goel, S., DeCristo, M.J., Watt, A.C., BrinJones, H., Sceneay, J., Li, B.B., Khan, N., Ubellacker, J.M., Xie, S., Metzger-Filho, O., et al. (2017). CDK4/6 inhibition triggers anti-tumour immunity. Nature *548*, 471–475.

60. Acerbi, I., Cassereau, L., Dean, I., Shi, Q., Au, A., Park, C., Chen, Y.Y., Liphardt, J., Hwang, E.S., and Weaver, V.M. (2015). Human breast cancer invasion and aggression correlates with ECM stiffening and immune cell infiltration. Integr. Biol. *7*, 1120–1134.

61. Desir, S., O'Hare, P., Vogel, R.I., Sperduto, W., Sarkari, A., Dickson, E.L., Wong, P., Nelson, A.C., Fong, Y., Steer, C.J., et al. (2018). Chemotherapy-induced tunneling nanotubes mediate intercellular drug efflux in pancreatic cancer. Sci. Rep. *8*, 9484.

62. Ogawa, L.M., and Baserga, S.J. (2017). Crosstalk between the nucleolus and the DNA damage response. Mol. Biosyst. *13*, 443–455.

63. Saki, M., and Prakash, A. (2017). DNA damage related crosstalk between the nucleus and mitochondria. Free Radic. Biol. Med. *107*, 216–227.

64. Palm, W., Park, Y., Wright, K., Pavlova, N.N., Tuveson, D.A., and Thompson, C.B. (2015). The utilization of extracellular proteins as nutrients is suppressed by mTORC1. Cell *162*, 259–270.

65. Bandola-Simon, J., and Roche, P.A. (2019). Dysfunction of antigen processing and presentation by dendritic cells in cancer. Mol. Immunol. *113*, 31–37.

66. Villamil Giraldo, A.M., Appelqvist, H., Ederth, T., and Ollinger, K. (2014). Lysosomotropic agents: impact on lysosomal membrane permeabilization and cell death. Biochem. Soc. Trans. *42*, 1460–1464.

67. Fassl, A., Brain, C., Abu-Remaileh, M., Stukan, I., Butter, D., Stepien, P., Feit, A.S., Bergholz, J., Michowski, W., Otto, T., et al. (2020). Increased lysosomal biomass is responsible for the resistance of triple-negative breast cancers to CDK4/6 inhibition. Sci. Adv. *6*, eabb2210.

68. Llanos, S., Megias, D., Blanco-Aparicio, C., Hernandez-Encinas, E., Rovira, M., Pietrocola, F., and Serrano, M. (2019). Lysosomal trapping of palbociclib and its functional implications. Oncogene *38*, 3886–3902.

69. Collins, K.P., Witta, S., Coy, J.W., Pang, Y., and Gustafson, D.L. (2021). Lysosomal biogenesis and implications for hydroxychloroquine disposition. J. Pharmacol. Exp. Ther. *376*, 294–305.

70. Saha, J., Kim, J.H., Amaya, C.N., Witcher, C., Khammanivong, A., Korpela, D.M., Brown, D.R., Taylor, J., Bryan, B.A., and Dickerson, E.B.

CellPress
OPEN ACCESS

(2020). Propranolol sensitizes vascular sarcoma cells to doxorubicin by altering lysosomal drug sequestration and drug efflux. Front. Oncol. *10*, 614288.

71. Li, A., Keck, J.M., Parmar, S., Patterson, J., Labrie, M., Creason, A.L., Johnson, B.E., Downey, M., Thomas, G., Beadling, C., et al. (2021). Characterizing advanced breast cancer heterogeneity and treatment resistance through serial biopsies and comprehensive analytics. NPJ Precis Oncol. *5*, 28.
72. Bardia, A., Hurvitz, S.A., DeMichele, A., Clark, A.S., Zelnak, A., Yardley, D.A., Karuturi, M., Sanft, T., Blau, S., Hart, L., et al. (2021). Phase I/II trial of exemestane, ribociclib, and everolimus in women with HR(+)/HER2(-) advanced breast cancer after progression on CDK4/6 inhibitors (TRINITI-1). Clin. Cancer Res. *27*, 4177–4185.
73. Voorwerk, L., Slagter, M., Horlings, H.M., Sikorska, K., van de Vijver, K.K., de Maaker, M., Nederlof, I., Kluin, R.J.C., Warren, S., Ong, S., et al. (2019). Immune induction strategies in metastatic triple-negative breast cancer to enhance the sensitivity to PD-1 blockade: the TONIC trial. Nat. Med. *25*, 920–928.
74. Nelson, E.K., Piehler, B., Eckels, J., Rauch, A., Bellew, M., Hussey, P., Ramsay, S., Nathe, C., Lum, K., Krouse, K., et al. (2011). LabKey Server: an open source platform for scientific data integration, analysis and collaboration. BMC Bioinformatics *12*, 71.
75. Afgan, E., Baker, D., Batut, B., van den Beek, M., Bouvier, D., Cech, M., Chilton, J., Clements, D., Coraor, N., Gruning, B.A., et al. (2018). The Galaxy platform for accessible, reproducible and collaborative biomedical analyses: 2018 update. Nucleic Acids Res. *46*, W537–W544.
76. Allan, C., Burel, J.M., Moore, J., Blackburn, C., Linkert, M., Loynton, S., Macdonald, D., Moore, W.J., Neves, C., Patterson, A., et al. (2012). OMERO: flexible, model-driven data management for experimental biology. Nat. Methods *9*, 245–253.
77. Merker, J.D., Oxnard, G.R., Compton, C., Diehn, M., Hurley, P., Lazar, A.J., Lindeman, N., Lockwood, C.M., Rai, A.J., Schilsky, R.L., et al. (2018). Circulating tumor DNA analysis in patients with cancer: American Society of Clinical Oncology and College of American Pathologists joint review. J. Clin. Oncol. *36*, 1631–1641.
78. Riesterer, J., Stempinski, E., and Lopez, C. (2020). Sample fixation of biopsy tissue for Electron Microscopy (EM). https://doi.org/10.17504/protocols.io.4bigske.
79. Riesterer, J., Stempinski, E., and Lopez, C. (2019). Post-fixation heavy metal staining and resin embedding for electron microscopy (EM). https://doi.org/10.17504/protocols.io.36vgre6.
80. Riesterer, J., Stempinski, E., and Lopez, C.S. (2020). 2D and 3D electron microscopy (EM) imaging of tissue biopsies and resections. https://doi.org/10.17504/protocols.io.bg58jy9w.
81. Schneider, C.A., Rasband, W.S., and Eliceiri, K.W. (2012). NIH Image to ImageJ: 25 years of image analysis. Nat. Methods *9*, 671–675.
82. Belevich, I., Joensuu, M., Kumar, D., Vihinen, H., and Jokitalo, E. (2016). Microscopy image browser: a platform for segmentation and analysis of multidimensional datasets. Plos Biol. *14*, e1002340.
83. Edelstein, A., Amodaj, N., Hoover, K., Vale, R., and Stuurman, N. (2010). Computer control of microscopes using microManager. Curr. Protoc. Mol. Biol. *Chapter 14*, Unit14 20.
84. Nickerson, A., Huang, T., Lin, L.J., and Nan, X. (2015). Photoactivated localization microscopy with bimolecular fluorescence complementation (BiFC-PALM). J. Vis. Exp. *106*, e53154.
85. Yoon, S.H., Kim, K.W., Goo, J.M., Kim, D.-W., and Hahn, S. (2016). Observer variability in RECIST-based tumour burden measurements: a meta-analysis. Eur. J. Cancer *53*, 5–15.
86. Kurland, B.F., Peterson, L.M., Shields, A.T., Lee, J.H., Byrd, D.W., Novakova-Jiresova, A., Muzi, M., Specht, J.M., Mankoff, D.A., Linden, H.M., et al. (2019). Test-retest reproducibility of (18)F-FDG PET/CT uptake in cancer patients within a qualified and calibrated local network. J. Nucl. Med. *60*, 608–614.
87. Li, H., and Durbin, R. (2010). Fast and accurate long-read alignment with Burrows–Wheeler transform. Bioinformatics *26*, 589–595.
88. McKenna, A., Hanna, M., Banks, E., Sivachenko, A., Cibulskis, K., Kernytsky, A., Garimella, K., Altshuler, D., Gabriel, S., Daly, M., et al. (2010). The Genome Analysis Toolkit: a MapReduce framework for analyzing next-generation DNA sequencing data. Genome Res. *20*, 1297–1303.
89. Karczewski, K.J., Francioli, L.C., Tiao, G., Cummings, B.B., Alfoldi, J., Wang, Q., Collins, R.L., Laricchia, K.M., Ganna, A., Birnbaum, D.P., et al. (2020). The mutational constraint spectrum quantified from variation in 141,456 humans. Nature *581*, 434–443.
90. Li, H. (2011). A statistical framework for SNP calling, mutation discovery, association mapping and population genetical parameter estimation from sequencing data. Bioinformatics *27*, 2987–2993.
91. Paradis, E., and Schliep, K. (2019). Ape 5.0: an environment for modern phylogenetics and evolutionary analyses in R. Bioinformatics *35*, 526–528.
92. Talevich, E., Shain, A.H., Botton, T., and Bastian, B.C. (2016). CNVkit: genome-wide copy number detection and visualization from targeted DNA sequencing. PLoS Comput. Biol. *12*, e1004873.
93. Wood, M.A., Nguyen, A., Struck, A.J., Ellrott, K., Nellore, A., and Thompson, R.F. (2020). Neoepiscope improves neoepitope prediction with multivariant phasing. Bioinformatics *36*, 713–720.
94. Szolek, A., Schubert, B., Mohr, C., Sturm, M., Feldhahn, M., and Kohlbacher, O. (2014). OptiType: precision HLA typing from next-generation sequencing data. Bioinformatics *30*, 3310–3316.
95. O'Donnell, T.J., Rubinsteyn, A., Bonsack, M., Riemer, A.B., Laserson, U., and Hammerbacher, J. (2018). MHCflurry: open-source class I MHC binding affinity prediction. Cell Syst. *7*, 129–132.e124.
96. Kennedy, S.R., Schmitt, M.W., Fox, E.J., Kohrn, B.F., Salk, J.J., Ahn, E.H., Prindle, M.J., Kuong, K.J., Shen, J.C., Risques, R.A., et al. (2014). Detecting ultralow-frequency mutations by duplex sequencing. Nat. Protoc. *9*, 2586–2606.
97. Boniface, C., Deig, C., Halsey, C., Kelley, T., Heskett, M.B., Thomas, C.R., Jr., Spellman, P.T., and Nabavizadeh, N. (2021). The feasibility of patient-specific circulating tumor DNA monitoring throughout multi-modality therapy for locally advanced esophageal and rectal cancer: a potential biomarker for early detection of subclinical disease. Diagnostics *11*, 73.
98. Montoya, J.A., GF, P., and González-Sánchez, D. (2019). Statistical inference for the Weitzman overlapping coefficient in a family of distributions. Appl. Math. Model. *71*, 558–568.
99. Adalsteinsson, V.A., Ha, G., Freeman, S.S., Choudhury, A.D., Stover, D.G., Parsons, H.A., Gydush, G., Reed, S.C., Rotem, D., Rhoades, J., et al. (2017). Scalable whole-exome sequencing of cell-free DNA reveals high concordance with metastatic tumors. Nat. Commun. *8*, 1324.
100. Tatlow, P.J., and Piccolo, S.R. (2016). A cloud-based workflow to quantify transcript-expression levels in public cancer compendia. Sci. Rep. *6*, 39259.
101. Bray, N.L., Pimentel, H., Melsted, P., and Pachter, L. (2016). Near-optimal probabilistic RNA-seq quantification. Nat. Biotechnol. *34*, 525–527.
102. Frankish, A., Diekhans, M., Ferreira, A.M., Johnson, R., Jungreis, I., Loveland, J., Mudge, J.M., Sisu, C., Wright, J., Armstrong, J., et al. (2019). GENCODE reference annotation for the human and mouse genomes. Nucleic Acids Res. *47*, D766–D773.
103. Soneson, C., Love, M.I., and Robinson, M.D. (2015). Differential analyses for RNA-seq: transcript-level estimates improve gene-level inferences. F1000Res. *4*, 1521.
104. Peixoto, L., Risso, D., Poplawski, S.G., Wimmer, M.E., Speed, T.P., Wood, M.A., and Abel, T. (2015). How data analysis affects power, reproducibility and biological insight of RNA-seq studies in complex datasets. Nucleic Acids Res. *43*, 7664–7674.

105. Risso, D., Ngai, J., Speed, T.P., and Dudoit, S. (2014). Normalization of RNA-seq data using factor analysis of control genes or samples. Nat. Biotechnol. *32*, 896–902.

106. Leek, J.T., Johnson, W.E., Parker, H.S., Jaffe, A.E., and Storey, J.D. (2012). The sva package for removing batch effects and other unwanted variation in high-throughput experiments. Bioinformatics *28*, 882–883.

107. Jassal, B., Matthews, L., Viteri, G., Gong, C., Lorente, P., Fabregat, A., Sidiropoulos, K., Cook, J., Gillespie, M., Haw, R., et al. (2020). The reactome pathway knowledgebase. Nucleic Acids Res. *48*, D498–D503.

108. Akbani, R., Ng, P.K., Werner, H.M., Shahmoradgoli, M., Zhang, F., Ju, Z., Liu, W., Yang, J.Y., Yoshihara, K., Li, J., et al. (2014). A pan-cancer proteomic perspective on the Cancer Genome Atlas. Nat. Commun. *5*, 3887.

109. Saldanha, A.J. (2004). Java Treeview–extensible visualization of microarray data. Bioinformatics *20*, 3246–3248.

110. Eisen, M.B., Spellman, P.T., Brown, P.O., and Botstein, D. (1998). Cluster analysis and display of genome-wide expression patterns. Proc. Natl. Acad. Sci. U S A *95*, 14863–14868.

111. Molania, R., Gagnon-Bartsch, J.A., Dobrovic, A., and Speed, T.P. (2019). A new normalization for Nanostring nCounter gene expression data. Nucleic Acids Res. *47*, 6073–6083.

112. Sivagnanam, S., Betts, C., and Coussens, L. (2021). HTAN multiplex IHC image cytometry V0.1. https://doi.org/10.17504/protocols.io.bsqjndun.

113. Bay, H., Ess, A., Tuytelaars, T., and Van Gool, L. (2008). Speeded-up robust features (SURF). Comput. Vis. Image Understand. *110*, 346–359.

114. McQuin, C., Goodman, A., Chernyshev, V., Kamentsky, L., Cimini, B.A., Karhohs, K.W., Doan, M., Ding, L., Rafelski, S.M., Thirstrup, D., et al. (2018). CellProfiler 3.0: next-generation image processing for biology. PLoS Biol. *16*, e2005970.

115. Eng, J. (2021). SMMART-BC.https://github.com/engjen/SMMART-BC.

116. Young Hwan, C., Thibault, G., Madin, O., Azimi, V., Meyers, C., Johnson, B., Link, J., Margolin, A., and Gray, J.W. (2017). Deep learning based nucleus classification in pancreas histological images. Annu. Int. Conf. IEEE Eng. Med. Biol. Soc. *2017*, 672–675.

117. Stringer, C., Wang, T., Michaelos, M., and Pachitariu, M. (2021). Cellpose: a generalist algorithm for cellular segmentation. Nat. Methods *18*, 100–106.

118. Johnson, W.E., Li, C., and Rabinovic, A. (2007). Adjusting batch effects in microarray expression data using empirical Bayes methods. Biostatistics *8*, 118–127.

119. Wolf, F.A., Angerer, P., and Theis, F.J. (2018). SCANPY: large-scale single-cell gene expression data analysis. Genome Biol. *19*, 15.

120. Traag, V.A., Waltman, L., and van Eck, N.J. (2019). From Louvain to Leiden: guaranteeing well-connected communities. Sci. Rep. *9*, 5233.

121. napari Contributors (2019). napari: a multi-dimensional image viewer for python. https://doi.org/10.5281/zenodo.3555620.

122. Virtanen, P., Gommers, R., Oliphant, T.E., Haberland, M., Reddy, T., Cournapeau, D., Burovski, E., Peterson, P., Weckesser, W., Bright, J., et al. (2020). SciPy 1.0: fundamental algorithms for scientific computing in Python. Nat. Methods *17*, 261–272.

STAR★METHODS

KEY RESOURCES TABLE

REAGENT or RESOURCE	SOURCE	IDENTIFIER
Antibodies		
Mouse monoclonal anti-PD-1	Abcam	Cat# ab52587; RRID: AB_881954
Rabbit monoclonal anti-CD3	Thermo Fisher Scientific	Cat# MA1-90582; RRID: AB_1956722
Mouse monoclonal anti-RORgT	EMD Millipore Sigma	Cat# MABF81; RRID: AB_11205416
Mouse monoclonal anti-NKp46	R&D Systems	Cat# MAB1850; RRID: AB_2149153
Mouse monoclonal anti-CD8a	Thermo Fisher Scientific	Cat# MA5-13473; RRID: AB_11000353
Rabbit monoclonal anti-T-bet	Cell Signaling Technology	Cat# 13232; RRID: AB_2616022
Mouse monoclonal anti-GATA-3	BioCare Medical	Cat# CM 405 A; RRID: AB_10895444
Mouse monoclonal anti-FoxP3	Thermo Fisher Scientific	Cat# 14-4777-82; RRID: AB_467556
Rabbit monoclonal anti-PD-L1	Cell Signaling Technology	Cat# 13684; RRID: AB_2687655
Mouse monoclonal anti-CD20	Abcam	Cat# ab9475; RRID: AB_307267
Mouse monoclonal anti-CD20	Santa Cruz Biotechnology	Cat# sc-70582; RRID: AB_1120279
Mouse monoclonal anti-CD45	Thermo Fisher Scientific	Cat# 14-0459-82; RRID: AB_467274
Mouse monoclonal anti-Tryptase	Abcam	Cat# ab2378; RRID: AB_303023
Mouse monoclonal anti-CD68	Abcam	Cat# ab783; RRID: AB_306119
Rabbit monoclonal anti-CSF1R	Abcam	Cat# ab183316; RRID: AB_2885197
Mouse monoclonal anti-DC-SIGN (DC28)	Santa Cruz Biotechnology	Cat# sc-65740; RRID: AB_1121347
Mouse monoclonal anti-CD66b	BD Bioscience	Cat# 555723; RRID: AB_396066
Rat monoclonal anti-DC-LAMP	Novus	Cat# DDX0191P-100; RRID: AB_2827532
Mouse monoclonal anti-HLA-DPB1	Abcam	Cat# ab157210; RRID: AB_2827533
Mouse monoclonal anti-CD163	Thermo Fisher Scientific	Cat# MA5-11458; RRID: AB_10982556
Mouse monoclonal anti-CD4	Thermo Fisher Scientific	Cat# MA5-12259; RRID: AB_10989399
Mouse monoclonal anti-CD56	Thermo Fisher Scientific	Cat# 07-5603; RRID: AB_2532931
Mouse monoclonal anti-pan cytokeratin	Abcam	Cat# ab27988; RRID: AB_777047
Rabbit monoclonal anti-Ki67	Abcam	Cat# ab16667; RRID: AB_302459
Rabbit polyclonal anti-EOMES (Tbr2)	EMD Millipore Sigma	Cat# AB2283; RRID: AB_10806889
Mouse monoclonal anti-IDO	EMD Millipore Sigma	Cat# MAB10009; RRID: AB_1977068
Rabbit monoclonal anti-Granzyme-B	EMD Millipore Sigma	Cat# 262R-1; RRID: AB_2889344
Mouse monoclonal anti-IL-10	LifeSpan Bio	Cat# LS-B7411-500; RRID: AB_11233179
Rabbit monoclonal anti-ICOS/CD278	LifeSpan Bio	Cat# LS-C210350; RRID: AB_2827535
Rabbit monoclonal anti-CD4	Abcam	Cat# ab213215; RRID: AB_2861280
Rabbit monoclonal anti-CD8	Abcam	Cat# 4207-1; RRID: AB_764503
Mouse monoclonal anti-Siglec-1/CD169	Novus	Cat# NB 600-534; RRID: AB_526814
Rabbit monoclonal anti-CD11b	Abcam	Cat# ab133357; RRID: AB_2650514
Rabbit monoclonal anti-MHC class I (HLA A+ HLA B)	Abcam	Cat# 2307-1; RRID: AB_1267243
Rabbit monoclonal anti-CD11c	Abcam	Cat# ab52632; RRID: AB_2129793
Rabbit monoclonal anti-T-bet/Tbx21	Abcam	Cat# ab150440; RRID: AB_2889209
Mouse monoclonal anti-CCR2	R&D Systems	Cat# MAB150; RRID: AB_2247178
Mouse monoclonal anti-MHC class II HLA-DP/DR/DQ	LifeSpan Bio	Cat# LS-C58506-200; RRID: AB_1511620
Histofine Simple Stain Human MAX PO (Rat) (for Rat primary antibody)	Nacalai USA	Cat# 414311F
Histofine Simple Stain Human MAX PO (R) (for Rabbit primary antibody)	Nacalai USA	Cat# 414144F

(Continued on next page)

Continued

REAGENT or RESOURCE	SOURCE	IDENTIFIER
Histofine Simple Stain Human MAX PO (M) (for Mouse primary antibody)	Nacalai USA	Cat# 414134F
Mouse monoclonal anti-α-SMA	Santa Cruz	Cat# sc-32251; RRID: AB_262054
Rabbit monoclonal anti-CD3	Abcam	Cat# ab213608; RRID: AB_764498
Rabbit monoclonal anti-CD31	Abcam	Cat# ab218582; RRID: AB_2857973
Rabbit monoclonal anti-CD4	Abcam	Cat# ab196147
Rabbit monoclonal anti-CD44	Abcam	Cat# ab216647; RRID: AB_764499
Rabbit monoclonal anti-CD45	Abcam	Cat# ab200317; RRID: AB_726545
Rabbit monoclonal anti-CD45	Abcam	Cat# ab214437; RRID: AB_726545
Mouse monoclonal anti-CD68	Biolegend	Cat# 916104; RRID: AB_2616797
Mouse monoclonal anti-CD8	Abcam	Cat# ab213017; RRID: N/A
Mouse monoclonal anti-CK14	Abcam	Cat# ab7800; RRID: AB_306091
Rabbit monoclonal anti-CK17	Abcam	Cat# ab185032; RRID: AB_2889195
Mouse monoclonal anti-CK19	Biolegend	Cat# 628502; RRID: AB_439773
Rabbit monoclonal anti-CK5	Abcam	Cat# ab193894
Rabbit monoclonal anti-CK7	Abcam	Cat# ab203434
Rabbit monoclonal anti-Ecad	Abcam	Cat# ab201499
Rabbit monoclonal anti-ER	Abcam	Cat# ab205851; RRID: AB_2728817
Mouse monoclonal anti-HER2	Santa Cruz	Cat# sc-33684; RRID: AB_627996
Rabbit monoclonal anti-Ki67	Cell Signaling Technology	Cat# 12075; RRID: AB_2728830
Mouse monoclonal anti-PCNA	Cell Signaling Technology	Cat# 8580; RRID: AB_11178664
Rabbit monoclonal anti-Phospho-Histone H3	Cell Signaling Technology	Cat# 3465; RRID: AB_10695860
Rabbit monoclonal anti-Vimentin	Cell Signaling Technology	Cat# 9854; RRID: AB_10829352
Rabbit monoclonal anti-Collagen I	Abcam	Cat# ab215969
Mouse monoclonal anti-Collagen IV	Thermo Fisher Scientific	Cat# 51-9871-82; RRID: AB_10853027
Rabbit monoclonal anti-EGFR	Cell Signaling Technology	Cat# 5108; RRID: AB_10694337
Rabbit monoclonal anti-CK8	Abcam	Cat# ab192467; RRID: AB_2864346
Trastuzumab	Genentech	N/A
RPPA Antibodies	MD Anderson Cancer Center Functional Proteomics RPPA Core Facility	https://www.mdanderson.org/research/research-resources/core-facilities/functional-proteomics-rppa-core/antibody-information-and-protocols.html
nCounter Vantage 3D Protein Solid Tumor Panel for FFPE (D) Antibodies	Nanostring	https://www.nanostring.com/wp-content/uploads/2021/01/LBL-10372-03_nCounter_Vantage_3D_Protein_Solid_Tumor_Panel_D_Probe_List_FFPE.xlsx
Biological samples		
Universal Human Reference RNA	Agilent Technologies	Cat# 740000
Chemicals, peptides, and recombinant proteins		
Human Epidermal Growth Factor	Sigma-Aldrich	Cat# SRP3027
Human Epidermal Growth Factor	Cell Signaling Technology	Cat# 8916
Tris Buffered Saline-Tween (with 0.05% Tween-20, pH 7.4)	Boston Bioproducts	Cat# IBB-181R
McCoy's 5A (Modified) Medium	Thermo Fisher Scientific	Cat# 16600082
Fetal Bovine Serum	Thermo Fisher Scientific	Cat# 10082147
Alexa Fluor 647 NHS Ester (Succinimidy Ester)	Thermo Fisher Scientific	Cat# A37566

(*Continued on next page*)

Continued

REAGENT or RESOURCE	SOURCE	IDENTIFIER
Glucose Oxidase	Sigma-Aldrich	Cat# G2133–50KU
Catalase	Sigma-Aldrich	Cat# C100-50MG
Dextrose (D-Glucose), Anhydrous	Fisher Scientific	Cat# D16–500
Cysteamine	Sigma-Aldrich	Cat# 30070
Hematoxylin, ready-to-use	Dako/Agilent	Cat# S330130-2
Antigen Retrieval Citra Plus Solution (10x Concentrated)	BioGenex	Cat# HK0809K
Peroxidase and Alkaline Phosphatase Blocking Reagent (Dual Endogenous Enzyme-Block)	Dako/Agilent	Cat# S2003
AEC Substrate Kit, Peroxidase (HRP), (3-amino-9-ethylcarbazole)	Vector Laboratories	Cat# SK-4200
Mol Bio Grad Ethanol (200 proof)	Sigma-Aldrich	Cat# E7023-500ML
Citrate Monohydrate	Sigma-Aldrich	Cat# C1909; CAS: 5949-29-1
Target Retrieval Solution, pH 9 (10X)	Agilent	Cat# S236784-2
Phosphate Buffered Saline (10X)	Fisher Scientific	Cat# BP39920
Normal Goat Serum	Vector Laboratories	Cat# S-1000
Bovine Serum Albumin	Sigma-Aldrich	Cat# A7906
SlowFade Gold Antifade Mountant with DAPI	Thermo Fisher Scientific	Cat# S36938
Sodium Hydroxide (Pellets)	Fisher Scientific	Cat# S318-500; CAS: 1310-73-2
Hydrogen peroxide solution, 30% (w/w)	Sigma-Aldrich	Cat# H1009-500ML; CAS: 7722-84-1
Paraformaldehyde	Electron Microscopy Sciences	Cat# 15714; CAS: 30525-89-4
Glutaraldehyde	Electron Microscopy Sciences	Cat# 16120; CAS: 11-30-8
Sodium cacodylate	Electron Microscopy Sciences	Cat# 12300; CAS: 124-65-2
Sucrose	J.T. Baker	Cat# 4072; CAS: 57-50-1
Osmium tetroxide	Ted Pella, Inc.	Cat# 18463; CAS: 20816-12-0
Potassium ferricyanide	Sigma-Aldrich	Cat# 702587; CAS: 13746-66-2
Thiocarbohydrazide	Sigma-Aldrich	Cat# 223220; CAS: 2231-57-4
Uranyl acetate	Electron Microscopy Sciences	Cat# 22400; CAS: #541-09-3
Lead nitrate	Electron Microscopy Sciences	Cat# 17900; CAS: 10099-74-8
Aspartic acid	Sigma-Aldrich	Cat# 11195; CAS: 323194-76-9
Acetone	Electron Microscopy Sciences	Cat# 10014; CAS: 67-64-1
EMbed 812 Embedding Kit with BDMA	Electron Microscopy Sciences	Cat# 14121
**Critical commercial assays**		
nCounter FLEX analysis system	Nanostring	https://www.nanostring.com/products/ncounter-analysis-system/flex-system/
nCounter Vantage 3D Protein Solid Tumor Panel for FFPE (D)	Nanostring	Cat# VPODC-SPKP-HSTF-12
Agencourt AMPure XP PCR Purification Beads	Beckman-Coulter	Cat# A63880
2100 Bioanalyzer High Sensitivity DNA Kit	Agilent	Cat# 5067-4626
KAPA HyperPrep Kit	Roche	Cat# KR8500
KAPA HiFi Hotstart PCR master mix	Roche	Cat# KK8500
Qubit 3 HS dsDNA Assay Kit	Thermo Scientific	Cat# Q33231
xGen Hybridization and Wash Kit	IDT	Cat# 1080584
TruSeq RNA Library Prep Kit	Illumina	Cat# 20020189
Tempus xE	Tempus Labs, Inc.	https://www.tempus.com/genomic-profiling/

(*Continued on next page*)

Continued

REAGENT or RESOURCE	SOURCE	IDENTIFIER
GeneTrails Solid Tumor Panel	OHSU Knight Diagnostic Laboratories	https://knightdxlabs.ohsu.edu/home/test-details?id=GeneTrails+Comprehensive+Solid+Tumor+Panel
Reverse Phase Protein Array	MD Anderson Cancer Center Functional Proteomics RPPA Core Facility	https://www.mdanderson.org/research/research-resources/core-facilities/functional-proteomics-rppa-core.html
QIAamp DNA FFPE Tissue Kit	QIAGEN	Cat# 56404
DNeasy Blood & Tissue Kit	QIAGEN	Cat# 69504
NucleoSnap cfDNA Kit	Macherey-Nagel	Cat# 740300.10
SureSelectXT Reagent Kit, HiSeq Platform	Agilent Technologies	Cat# G9611B
SureSelectXT Human All Exon V5, 96	Agilent Technologies	Cat# 5190-6209
Deposited data		
gnomAD	[89]	https://gnomad.broadinstitute.org/
Human reference genome UCSC hg19 (GRCh37/hg19)	Genome Sequencing Consortium	https://genome.ucsc.edu/cgi-bin/hgGateway
GENCODE	[102]	https://www.gencodegenes.org/
Raw next-generation sequencing data	This work.	https://www.ncbi.nlm.nih.gov/projects/gap/cgi-bin/study.cgi?study_id=phs002371.v1.p1
TCGA BRCA gene expression	[100]	https://osf.io/gqrz9
MSigDB	[18]	https://www.gsea-msigdb.org/gsea/msigdb/
Processed next-generation sequencing data	This work.	Participant ID: HTA9_1; https://htan-portal-nextjs.vercel.app/explore
Protein expression data from RPPA and the Intracellular Signaling Protein Panel	This work.	Participant ID: HTA9_1; https://htan-portal-nextjs.vercel.app/explore
Raw and processed image data from CycIF, mIHC, and EM	This work.	Participant ID: HTA9_1; https://htan-portal-nextjs.vercel.app/explore
Processed images (web viewing)	This work.	Participant ID: HTA9_1; https://idp.tissue-atlas.org/
Experimental models: Cell lines		
Human MCF7	Characterized Cell Line Core (MDACC)	https://www.atcc.org/products/all/HTB-22.aspx; RRID: CVCL_0031
Human MDA-MB-468	Characterized Cell Line Core (MDACC)	https://www.atcc.org/products/all/HTB-132.aspx; RRID: CVCL_0419
Human MDA-MB-231	ATCC	https://www.atcc.org/products/all/HTB-26.aspx#generalinformation; RRID: CVCL_0062
Human BT474	ATCC	https://www.atcc.org/products/all/HTB-20.aspx; RRID: CVCL_0179
Human HCC1954	ATCC	https://www.atcc.org/products/crl-2338; RRID: CVCL_1259
Human SKBR3	ATCC	RRID: CVCL_0033; https://www.atcc.org/products/htb-30
HCC1143	ATCC	RRID: CVCL_1245; https://www.atcc.org/products/crl-2321
HCC3153	UT-Southwestern	RRID: CVCL_3377
T47D	ATCC	RRID: CVCL_0553; https://www.atcc.org/products/htb-133
AU565	ATCC	RRID: CVCL_1074; https://www.atcc.org/products/crl-2351

(*Continued on next page*)

Continued

REAGENT or RESOURCE	SOURCE	IDENTIFIER
MDAMB436	ATCC	RRID: CVCL_0623; https://www.atcc.org/products/htb-130
**Oligonucleotides**		
See Table S2	This paper	N/A
**Software and algorithms**		
LabVantage	LabVantage Solutions Inc.	https://www.labvantage.com/
LabKey	[74]	https://www.labkey.com/
Removal of Unwanted Variation (RUV-III)	[111]	https://cran.r-project.org/package=ruv
Removal of Unwanted Variation (RUVSeq)	[105]	https://bioconductor.org/packages/release/bioc/html/RUVSeq.html
MATLAB	MathWorks	https://www.mathworks.com/products/matlab.html
syngo.via	Siemens Healthcare GmbH	https://www.siemens-healthineers.com/en-us/molecular-imaging/pet-ct/syngo-via
Horos	Nimble Co LLC	https://horosproject.org/download-horos/
Cluster 3.0	[110]	http://bonsai.hgc.jp/~mdehoon/software/cluster/software.htm
TreeView	[109]	http://jtreeview.sourceforge.net/
MATLAB, Computer vision toolbox	MathWorks	https://www.mathworks.com/help/vision/
SURF algorithm (for MATLAB)	[113]	https://www.mathworks.com/help/vision/ref/detectsurffeatures.html
ImageJ	[81]	https://imagej.nih.gov/ij/
RGB to CMYK (FIJI Plugin)	[81]	https://imagej.net/tutorials/rgb-to-cmyk
CellProfiler 3.0	[114]	https://cellprofiler.org/
FCS Express Image Cytometry RUO	DeNovo Software	https://denovosoftware.com/
mpileup (Samtools)	[90]	http://samtools.sourceforge.net/
BWA-MEM	[87]	http://bio-bwa.sourceforge.net/
GATK	[88]	https://gatk.broadinstitute.org/hc/en-us
Galaxy	[75]	https://galaxyproject.org/
ape	[91]	http://ape-package.ird.fr/
CNVkit	[92]	https://github.com/etal/cnvkit
Trim Galore	Babraham Institute	https://www.bioinformatics.babraham.ac.uk/projects/trim_galore/
Kallisto	[101]	https://github.com/pachterlab/kallisto
tximport	[103]	https://bioconductor.org/packages/release/bioc/html/tximport.html
SVA/Combat	[106]	https://bioconductor.org/packages/release/bioc/html/sva.html
GSVA	[17]	https://www.bioconductor.org/packages/release/bioc/html/GSVA.html
Pathway Commons	[24]	https://www.pathwaycommons.org/
Transcriptional Regulator Analysis	This work.	https://dx.doi.org/10.5281/zenodo.5608590
CausalPath	[25]	https://causalpath.org/; https://github.com/PathwayAndDataAnalysis/causalpath
Neoepiscope	[93]	https://github.com/pdxgx/neoepiscope
OptiType	[94]	https://github.com/FRED-2/OptiType
MHCflurry	[95]	https://github.com/openvax/mhcflurry
DIDA-Seq Error Correction Scripts	[14]	https://github.com/ohsu-cedar-comp-hub/DIDA-Seq

(*Continued on next page*)

Continued

REAGENT or RESOURCE	SOURCE	IDENTIFIER
R 3.6	R Foundation for Statistical Computing	https://www.R-project.org/
DIDA-Seq Bayesian Overlap Script	[97]	https://github.com/mheskett/SMMART
Zen 2.3 Slidescan	Zeiss	https://www.zeiss.com/microscopy/us/products/microscope-software.html
Cellpose	[117]	https://github.com/MouseLand/cellpose
ComBat	[118]	https://doi.org/10.1093/biostatistics/kxj037
scanpy	[119]	https://github.com/theislab/scanpy
SciPy	[122]	https://github.com/scipy/scipy
Umap	https://arxiv.org/abs/1802.03426	https://github.com/lmcinnes/umap
napari	[121]	https://zenodo.org/record/3555620
OMERO	[76]	https://www.openmicroscopy.org/omero/
Cyclic Immunofluorescence Analysis	This work.	https://dx.doi.org/10.5281/zenodo.5637447
maps2ometiff	This work.	https://dx.doi.org/10.5281/zenodo.5608828
em_segmentation	https://doi.org/10.1101/2021.05.27.446019	https://github.com/archana2890/em_segmentation
Microscopy Image Browser	[82]	http://mib.helsinki.fi/
Amira Software	Thermo Fisher Scientific	https://www.thermofisher.com/us/en/home/electron-microscopy/products/software-em-3d-vis/amira-software.html
Dragonfly	Object Research Systems	https://www.theobjects.com/dragonfly/
IrfanView	Irfan Skiljan	https://www.irfanview.com/
Maps Software	Thermo Fisher Scientific	https://www.thermofisher.com/us/en/home/electron-microscopy/products/software-em-3d-vis/maps-software.html
ichorCNA	[99]	https://github.com/broadinstitute/ichorCNA
microManager	[83]	https://micro-manager.org/Download_Micro-Manager_Latest_Release
Matlab packages for raw PALM/STORM image data processing	[84]	https://www.ohsu.edu/school-of-medicine/nan-lab/resources
**Other**		
Signature Series Cover Glass	Thermo Fisher Scientific	Cat# 12460S
Microscope Slides	Mercedes Scientific	Cat# TNR WHT45AD
Plastic Coverslips	IHC World	Cat# IW-2601
Rectangular Cover Glass	Corning	Cat# 2980-243, 2980-245
Nunc Lab-Tek II Chambered Coverglass	Thermo Fisher Scientific	Cat# 155409
SEM pin stub	Ted Pella, Inc.	Cat# 16145
Leitsilber conductive paint	Ted Pella, Inc.	Cat# 16035
H20E Epo-TEK Silver conductive epoxy	Ted Pella, Inc.	Cat# 16014
Fisherbrand Transfer pipettes	Fisher Scientific	Cat# 13-711-7M
Axygen Centrifuge microtubes	Fisher Scientific	Cat# 14-222-180
Cytivia Whatman filter paper	Fisher Scientific	Cat# 1001-090
Flat embedding mold	Ted Pella, Inc.	Cat# 10504

(*Continued on next page*)

Continued

REAGENT or RESOURCE	SOURCE	IDENTIFIER
Helios NanoLab G3 DualBeam FIB-SEM	FEI Company (now Thermo Fisher Scientific)	https://www.thermofisher.com/us/en/home/electron-microscopy/products/dualbeam-fib-sem-microscopes.html
Gemini 550 Crossbeam FIB-SEM	ZEISS International	https://www.zeiss.com/microscopy/us/products/fib-sem-instruments/crossbeam.html
Ultramicrotome Leica EM UC7	Leica Microsystems	https://www.leica-microsystems.com/products/sample-preparation-for-electron-microscopy/p/leica-em-uc7/
Trim90 diamond knife	DiATOME	https://www.diatomeknives.com/product.aspx?pid=416
EM ACE600 High Vacuum Sputter Coater	Leica Microsystems	https://www.leica-microsystems.com/products/sample-preparation-for-electron-microscopy/p/leica-em-ace600/
Managed workforce (800 hours)	CloudFactory	https://www.cloudfactory.com/

RESOURCE AVAILABILITY

Lead contact

Further information and requests for resources and reagents should be directed to and will be fulfilled by the lead contact, Joe Gray (grayjo@ohsu.edu).

Materials availability

This study did not generate new unique reagents.

Data and code availability

- Raw data generated by next-generation sequencing platforms have been deposited at dbGaP and are publicly available as of the date of publication. The project accession number is listed in the key resources table. Processed next-generation sequencing data, protein expression data, as well as raw and processed image data have been deposited with the HTAN Data Coordinating Center. They are publicly available through the HTAN Data Portal as part of the HTAN OHSU Atlas as of the date of publication. The case number is listed in the key resources table. Processed images are also available for web-based viewing through the HTAN Imaging Data Portal as of the date of publication. The case number is listed in the key resources table. This paper also analyzes existing, publicly available data. The sources for these datasets are listed in the key resources table.
- All original code has been deposited at Zenodo and is publicly available as of the date of publication. DOIs are listed in the key resources table.
- Any additional information required to reanalyze the data reported in this paper is available from the lead contact upon request.

EXPERIMENTAL MODEL AND SUBJECT DETAILS

Human subjects

This study was approved by the Oregon Health & Science University (OHSU) Institutional Review Board (IRB). All biospecimens and data were acquired and analyzed under the OHSU IRB-approved protocols Molecular Mechanisms of Tumor Evolution and Resistance to Therapy (IRB#16113) and Reconstructing the Tumor Genome in Peripheral Blood (IRB#10163). Participant eligibility was determined by the enrolling physician and informed written consent was obtained from all subjects. This study includes a single 64 year-old female woman (HTAN participant ID HTA9_1).

Cell lines

Cell line SKBR3 was cultured in McCoy's 5A (modified) Medium supplemented with 10% Fetal Bovine Serum (FBS). For cell lines used as controls for the Intracellular Signaling Protein Panel, MCF7, and MDAMB468 were acquired from the Characterized Cell

Line Core (CCLC) while MDAMB231, BT474, and HCC1954 were acquired from the American Type Culture Collection (ATCC). MCF7, MDAMB468, and MDAMB231 were cultured in Dulbecco's Modified Eagle's Medium (DMEM) with 10% FBS. BT474 was cultured in ATCC Hybri-Care Medium with 10% FBS. HCC1954 was cultured in Roswell Park Memorial Institute 1640 (RPMI1640) with 10% FBS. For cell lines used as controls during CycIF, HCC1143, T47D, BT474, AU565, and MDAMB436 were acquired from the ATCC while HCC3153 was acquired from UT-Southwestern. MDAMB436 was cultured in DMEM with 10% FBS. BT474, HCC1143, T47D, AU565, and HCC3153 were cultured in RPMI1640 with 10% FBS. All cell lines were derived from human female breast cancers, incubated in 5% CO2 at 37°C, and grown to near confluency. Genomic DNA was extracted from each cell line using DNeasy Blood and Tissue Kits (QIAGEN) and submitted to either Labcorp Cell Line Testing (Genetica) or the MD Anderson Cytogenetics and Cell Authentication Core (CCAC) for short tandem repeat (STR) analysis. Cell line identities were confirmed by comparison with reference STR profiles from the ATCC, DSMZ STR, or CCAC database.

METHOD DETAILS

Clinical decision making

All clinical decisions were the treating physicians' discretion, with reference to clinical analytics results, established treatment guidelines, appropriate data from clinical trials, and input from an IRB-approved multidisciplinary tumor board. Research Use Only (RUO) data from exploratory analytics were not used for clinical decision making. In Phase 1, palbociclib and fulvestrant were used as the standard frontline for progression on adjuvant aromatase inhibitors. Everolimus was added due to rising tumor markers and clinical concern for symptomatic progression as well as emerging data from the TRINITI-1 trial that suggested PI3K/AKT/mTOR pathway targeting along with CDK4/6 to prevent resistance.[72] In Phase 2, doxorubicin was introduced to counter progressive disease. Pembrolizumab was added to doxorubicin based on emerging data from the TONIC trial suggesting that anthracyclines (and platinum) were the best immunotherapy combination.[73] In Phase 3, doxorubicin was replaced with capecitabine to counter progressive disease. Enzalutamide was added because of increased high expression of AR. Fulvestrant was added to counter persistent ER signaling. Pembrolizumab was continued since it was well-tolerated in Phase 2. Carboplatin was introduced in Phase 4 to counter disease progression.

Drugs given to moderate aspects of therapy-induced toxicities included (a) denosumab to reduce risk of skeletal related events due to bone metastases; (b) pegfilgrastim to stimulate production of neutrophils; and (c) hydroxychloroquine for suspected drug-induced amyopathic dermatomyositis as recommended by dermatology.

Radiology

FDG-PET/CT imaging was performed according to the standard institutional protocol, with patients fasting for 6 hours following 24 hours of rest. Prior to the examination and FDG injection, blood glucose levels were confirmed to be less than 200 mg/dL. The patient received a dose of ^{18}F-FDG of 370 to 555 MBq (10–15 mCi) on the basis of body weight. After an uptake period of 90 minutes, a vertex-to-mid-thigh FDG-PET/CT scan was performed using 3 min/bed position on a CTI Biograph duo PET/CT scanner (Siemens Medical Systems, Hoffman Estates, Illinois, USA) or a CTI Biograph TruePoint 40 PET/CT scanner (Siemens Medical Systems, Knoxville, Tennessee, USA). CT imaging was performed according to the standard institutional protocol from clavicles to mid-thigh on a Phillips Brilliance CT 128slice helical scanner (Philips Medical Systems, Amsterdam, NE).

Clinical and exploratory workflows

All blood and metastatic biopsy biospecimens used in this study were prospectively collected by trained study coordinators. Preservation procedures for biopsy tissue were started within two to five minutes of removal of tissue from the patient in order to preserve the molecular and architectural features of the tumor that may quickly degrade. Clinical analytics were performed in CLIA-certified, CAP-accredited laboratories. RUO exploratory analyses were performed in academic research laboratories or core facilities. Both manual and automated abstraction from the patient's medical record were used to generate the clinical metadata, including detailed information about anticancer treatments and supportive care for integration with the analytic results.

Biospecimens were tracked and managed using a custom implementation of the LabVantage laboratory information management system. The LabKey system was used to store and visualize both clinical data and analytic results.[74] The Galaxy computational workbench was used to create and run multi-step analysis workflows that process raw omics and imaging datasets.[75] The OMERO system was used to visualize multiplex imaging and electron microscopy datasets.[76]

GeneTrails® solid tumor panel

Formalin-fixed biopsy tissue was submitted to the CLIA-certified/CAP-accredited OHSU Knight Diagnostic Laboratories for targeted DNA sequencing with the clinical GeneTrails Solid Tumor Panel assay. There, genomic DNA was extracted from macro-dissected, tumor-rich regions of FFPE. Next-generation sequencing libraries were prepared using custom QIASeq chemistry (QIAGEN) with multiplexed PCR and sequenced on an Illumina NextSeq500/550. The DNA library was generated by 9,229 custom-designed primer extension assays covering 613,343 base pairs across 225 cancer-related genes (including whole exons of 199 genes and hotspot regions of 26 genes). This panel is routinely sequenced to an average read depth of >2,000, providing high sensitivity for SNVs, short insertions/deletions, and copy number alterations. All variants identified were reported out clinically.

Blood collection and DNA isolation

Up to 40 mL (range 6–40 mL) of blood were collected in 5 × 6-mL or 4 × 10-mL, purple-capped EDTA tubes. Consistent with published recommendations, blood plasma was isolated within 6 hours of collection by first spinning whole blood at 1000g for 10 min, separating the top plasma layer into 1 mL aliquots, then spinning those aliquots at 15,000g for 10 min, transferring the supernatant to cryovials, and storing at −80°C.[77] DNA extraction of tumor tissue from FFPE was carried out using QIAamp DNA FFPE Tissue Kit (QIAGEN). DNA was extracted from plasma using the NucleoSnap cfDNA kit (Macherey-Nagel) and from buffy coat using the DNeasy Blood & Tissue Kit (QIAGEN). DNA isolated from both FFPE samples and buffy coat were fragmented by sonication to 150 bp using a Covaris E220 prior to library preparation.

Whole exome sequencing

Sequencing libraries were prepared from 100-500 ng of cell free DNA (cfDNA) or sonicated genomic DNA using the KAPA Hyper-Prep Kit (KAPA Biosystems), enriched using the SureSelectXT Target Enrichment System (Agilent Technologies) and the SureSelectXT Human All Exon V5 capture baits (Agilent Technologies). Next generation sequencing was carried out using the Illumina NextSeq500 or HiSeq 2500 platform with 2x79-144 cycles by the OHSU Massively Parallel Sequencing Shared Resource to an average depth of 100x per library replicate. For Bx3 and Bx4 only, DNA isolated from both FFPE samples and buffy coat were submitted to Tempus Labs, Inc. for whole exome sequencing (WES) with the Tempus xE assay (Tempus Labs, Inc., Chicago, IL, USA).

Dual index degenerate adaptor sequencing

Bait Design: Single nucleotide variants (SNVs) were filtered by frequency (>5% in the tumor and <2% in the matched normal) and depth (>30x in the tumor and >15x in a patient-matched matched buffy coat normal). A set of 20–40 SNVs per tumor tissue sample were then hand-selected for bait design based on high variant allele frequency (VAF) and potential clinical relevance. In total, 55 mutations were selected with varying presence across the primary tumor and all four biopsies, to monitor longitudinal blood draws for the presence of tumor-derived circulating tumor DNA (ctDNA). Biotinylated oligonucleotides (IDT, Coralville, IA, USA) matching the 120bp UCSC GRCh37/hg19 human genome reference sequence spanning each mutation were synthesized for use as baits in hybridization capture library preparation (Table S2). Oligonucleotides for mutations in INVS and LILRA3 were eventually discarded for inconsistent coverage and high error rates.

Library Preparation: Dual Index Degenerate Adaptor Sequencing (DIDA-Seq) error-correction libraries were created using the KAPA Biosystems HyperPrep kit (KAPA Biosystems) using at least 30 ng of cell-free DNA (cfDNA) as input as previously described,[14] using a single over-night capture incubation instead of two incubations. Briefly, DIDA-Seq adaptors (Table S2) were ligated to extracted cfDNA using KAPA Hyper Prep Reagents and protocol (with 16°C overnight incubation) and then PCR amplified for 8 cycles using library amplification primers (Table S2). Approximately 250 ng of amplified libraries were pooled (4–5 libraries per pool) and overnight hybridization capture and purification was carried out using the xGen Hybridization and Wash kit (IDT, Coralville, IA, USA). Libraries were then PCR amplified for 10 cycles as before. Amplified libraries were then purified 1:1 with Agencourt AMPure XP beads (Beckman-Coulter) and assessed using the 2100 Bioanalyzer system (Agilent Technologies, Santa Clara, CA, USA) High Sensitivity dsDNA kit. Samples were then sequenced on either the Illumina HiSeq 2500, paired-end 100 bp with dual 14-bp indexing cycles (high-capacity, rapid run mode) or the Illumina NextSeq 500, paired-end 75 bp with dual 14-bp indexing cycles (high-capacity, 150-cycle kit).

Low-pass whole genome sequencing

Low-Pass Whole Genome Sequencing (LP-WGS) libraries were prepared with 50 ng of sonicated tumor DNA (extracted from FFPE as described above) and patient-matched buffy coat DNA using the KAPA Hyper-Prep kit (KAPA Biosystems) with Illumina single index TruSeq adaptors (IDT, Coralville, IA, USA) and sequenced on the Novaseq S4 platform (Illumina) to 0.9X mean coverage.

Whole transcriptome sequencing

Library construction and sequencing: RNA was extracted from macro-dissected, tumor-rich regions of FFPE at the OHSU Knight Diagnostic Laboratories. Sequencing libraries are constructed with the TruSeq RNA Library Prep Kit, followed by sequencing on Illumina NextSeq500. A Universal Human Reference RNA (UHR; Agilent Technologies) was sequenced with each batch of samples to allow for assessment and removal of technical artifacts (due to, e.g., library preparation).

Reverse phase protein arrays

Tumor tissue collected from core needle biopsies was flash frozen in liquid nitrogen within two to five minutes of removal from the patient and stored at −80°C. Frozen tissue was then submitted to the MD Anderson Cancer Center Functional Proteomics RPPA Core for proteomic profiling of the abundances of 450 proteins and phosphoproteins with the Reverse Phase Protein Array (RPPA) assay.[21,22]

Intracellular signaling protein panel

The GeneTrails® Intracellular Signaling Protein Panel is an assay validated for clinical use within the Knight Diagnostic Laboratories at OHSU. It utilizes the NanoString nCounter Vantage 3D Solid Tumor Panel for FFPE, including 23 antibodies, with 12 targeting

phosphorylated proteins, specifically designed to interrogate the MAPK and PI3K/mTOR signaling pathways (Table S2).[23] This multiplexed panel allows for the simultaneous quantification of multiple proteins from a single section of FFPE tissue. Four micrometer FFPE sections from six control cancer cell lines [MCF7, MDA-MB-468, MDA-MB-231, BT474, HCC1954, and human Epidermal Growth Factor (Sigma-Aldrich) treated MDA-MB-468] and tumor biopsy tissue were deparaffinized in Xylene, 100% ethanol, and 95% ethanol sequentially and subjected to citrate-based antigen retrieval (pH6.0) in a high-pressure cooker. Samples were blocked with blocking buffer (Buffer W) for 1 hour and incubated overnight at 4°C with the cocktail of target-specific oligonucleotide-tagged antibodies. After washing with TBS-T buffer, the oligo-tags were released by UV light (3 minutes on a UV lightbox) and hybridized overnight with NanoString's four color-barcoded probe tagsets. Hybridized codesets (oligo target + probe tagset) were bound and immobilized to the nCounter cartridge during purification process. Target protein expression levels were semi-quantitatively measured with codeset molecule counts using the NanoString nCounter FLEX analysis system. Six cancer cell lines were selected as positive controls and were included on every run to assess antibody and control performance and to correct for batch effects.

Multiplex immunohistochemistry

Immunohistochemical staining: Glass mounted FFPE tissue sections (5 μm) were baked at 60°C for 60 minutes, deparaffinized with xylene, and rehydrated in serially graded alcohols, then place in distilled water. Slides were stained with hematoxylin (Dako) for 1 minute, mounted with 1x Tris Buffered Saline-Tween (TBST) buffer (Boston Bioproducts), coverslipped with Signature Series Cover Glass (Thermo Scientific), and subjected to whole slide digital scanning at 20x magnification using an Aperio ImageScope (Leica Biosystems). Slides were de-coverslipped with 1 min of agitation in TBST and subjected to heat-mediated antigen retrieval in 1x pH 6.0 citrate buffer (Biogenex Laboratories) for 20 min at 95°C, followed by blocking of endogenous peroxidase activity with Peroxidase and Alkaline Phosphatase Blocking Reagent (Dako, per manufacturer's instructions). Slides were then subjected to 12 cycles of multiplex immunohistochemistry (mIHC), each cycle consisted of either 1 or 2 rounds of IHC. Each round consisted of application of primary antibody, HRP-linked secondary antibody (Histofine Simple Stain Max PO, Nacalai USA, 414311F, 414144F, 414134F), and HRP-mediated development of AEC chromogen (Vector Laboratories, SK4200), followed by whole slide scanning. Citrate antigen retrieval was used between cycles to remove primary antibodies, and HRP inactivation was used between rounds (Dako, S2003, per manufacturer's instructions) to eliminate HRP carry-over as described previously.[27,28] Several antibody panels (and variations thereof) were utilized for the current study (Table S2). Each mIHC antibody panel required one FFPE tissue specimen, thus, in instances where more than one antibody panel was used on a single timepoint (e.g., PT, Bx1, and Bx2), serial sections were used. In all other instances (e.g., Bx3 and Bx4), only one FFPE tissue section was used for one antibody panel (Discovery). Where IHC and chromogenic staining did not pass QC, they were not included in analysis: e.g., PD-L1 and CSF1R on the myeloid panel, and CD68 and ICOS on the functional panel(s). Several antibodies were not common across all or some panels, thus not included in results: IDO on functional panel (Bx1), Tryptase on myeloid panel (Primary, Bx1 and Bx2), RORyT and GATA3 on the lymphoid panel (primary, Bx1, and Bx2), and CCR2, HLA class-I, CD169, CD11b, and CD11c on the discovery panel (23 antibodies) (Bx3 and Bx4).

Cyclic immunofluorescence

Immunofluorescence analysis of tumor tissue: FFPE biopsy tissues and control biospecimens prepared from normal breast tissue, tonsil, and six cell lines representing basal-like (HCC1143, HCC3153), claudin-low (MDAMB436), luminal (T47D), and HER2 positive (BT474, AU565) breast cancers were sectioned at 4 μm and mounted on adhesive microscope slides (Mercedes Scientific). The slides were baked overnight in an oven at 55°C (Robbin Scientific, Model 1000) and an additional 30 minutes at 65°C (Clinical Scientific Equipment, NO. 100). Tissues were deparaffinized with xylene and rehydrated with graded ethanol baths. Two step antigen retrieval was performed in the Decloaking Chamber (Biocare Medical) using the default settings. After completion of the first step in 10 mM citrate buffer pH 6 (Sigma-Aldrich) in the chamber, slides were further incubated in pre-boiled Target Retrieval Solution, pH 9 (Agilent) for 15 minutes. Slides were then washed in two brief changes of deionized water (diH_2O) for ~2 seconds and once for 5 minutes in 1x phosphate buffered saline (PBS), pH 7.4 (Fisher Scientific). Sections were blocked in 10% normal goat serum (NGS, Vector Laboratories), 1% bovine serum albumin (BSA, Sigma-Aldrich) in PBS for 30 minutes at 20°C in a humid chamber, followed by PBS washes. Direct labeled primary antibodies (Table S2, key resources table) were diluted in 5% NGS, 1% BSA in 1x PBS and applied overnight at 4°C in a humid chamber, covered with plastic coverslips (IHC World). Following overnight incubation, tissues were washed 3 × 10 min in 1x PBS. Rectangular Cover Glass (Corning) were mounted in SlowFade Gold Antifade Mountant plus DAPI mounting media (Thermo Fisher Scientific).

Fluorescence Microscopy: Fluorescently stained slides were scanned on the Zeiss AxioScan.Z1 (Zeiss, Germany) with a Colibri 7 light source (Zeiss). The filter cubes used for image collection were DAPI (Zeiss 96 HE), Alexa Fluor 488 (AF488, Zeiss 38 HE), AF555 (Zeiss 43 HE), AF647 (Zeiss 50), and AF750 (Chroma 49007 ET Cy7). The exposure time was determined individually for each slide and stain to ensure good dynamic range but not saturation. Full tissue scans were taken with the 20x objective (Plan-Apochromat 0.8NA WD = 0.55, Zeiss), and stitching was performed in Zen Blue image acquisition software (Zeiss).

Quenching Fluorescence Signal: After successful scanning, slides were soaked in 1x PBS for 10–30 minutes in a glass Coplin jar, waiting until the glass coverslip slid off without agitation. Quenching solution containing 20 mM sodium hydroxide (NaOH) and 3% hydrogen peroxide (H_2O_2) in 1x PBS was freshly prepared from stock solutions of 5 M NaOH and 30% H_2O_2, and each slide placed in 10 ml quenching solution. Slides were quenched under incandescent light, for 30 minutes for FFPE tissue slides. Slides were then

CellPress
OPEN ACCESS

removed from the chamber with forceps and washed times for 2 min in 1x PBS. The next round of primary antibodies was applied, diluted in blocking buffer as previously described, and imaging and quenching were repeated over ten rounds for FFPE tissue slides.

Scanning electron microscopy

Sample Fixation:[41,78] Tumor tissue for scanning electron microscopy (SEM) was collected at the time of biopsy and placed into SEM-specific fixative (2.5% paraformaldehyde, 2.5% glutaraldehyde in 0.1M sodium cacodylate buffer) as rapidly as possible to preserve tissue ultrastructure. Tissues were then stored in fixative at 4°C indefinitely until processing could take place. No tissue was available for SEM from Bx3.

Sample Preparation:[41,79] Tissue samples were prepared for SEM by post-fixation heavy metal infiltration followed by epoxy-resin embedding with the EMbed 812 Embedding Kit. Heavy metal staining using osmium tetroxide, uranyl acetate, and lead aspartate provided contrast for imaging by dissociating the metals and allowing them to bind to lipids and proteins within cellular membranes and organelles. After staining and resin embedding, polymerized blocks were mounted directly to SEM pin-style stubs (Ted Pella, Inc.) and trimmed to create a flat surface using a Ultramicrotome Leica EM UC7 (Leica Microsystems) equipped with trim 90 diamond knives (DiATOME). Mounted blocks were conductively coated with 8-nm carbon using an EM ACE600 High Vacuum Sputter Coater (Leica Microsystems).

Imaging:[41,80] Two-dimensional large-format SEM maps were collected on trimmed block faces using a Helios NanoLab G3 DualBeam^{TM} (FEI) focused ion-beam-scanning electron microscope (FIB-SEM) equipped with the Thermo Scientific Maps Software package. Using this software for automation, hundreds of tiled images were collected over the entire block surface and stitched together, creating a pyramidal viewing architecture that provides observations starting at the millimeter-scale and zooms all the way down to 4-nm/pixel spatial resolution. Imaging conditions were 3 keV, 200–400 pA, 4-mm working distance, and 3 μs dwell time using the concentric backscatter detector (CBS). A custom script converts these large maps from TIFF into OME-TIFF format (maps2ometiff, part of the ometiff_converters library) for web-based viewing and sharing via OMERO.[76]

Regions of interest for three-dimensional electron microscopy (3DEM) were selected from the high-resolution maps. Three separate 3DEM datasets collected using FIB-SEM technology were generated using vendor-specific automated serial-sectioning software: two high-resolution, small volumes (4-nm/voxel, 25 × 20 × 6–10 μm^3) on each respective biopsy and one lower resolution, larger volume (10-nm/voxel, 48 × 48 × 17 μm^3). The high-resolution image stacks were collected using the aforementioned Helios FIB-SEM with the same electron beam conditions and the In-Column Detector (ICD). The large volume was collected from the pretreatment biopsy using a Gemini 550 Crossbeam FIB-SEM (ZEISS International), using 1.5 keV, 1.0 nA, 5-mm working distance, 1.6 μs dwell time, and the Energy-Selective Backscatter (EsB) detector.

Segmentation: 2D SEM maps were manually reviewed using ImageJ and IrfanView.[81] Segmentation of image stacks was performed manually with the assistance of a CloudFactory managed workforce using Microscopy Image Browser.[82] Deep learning models were utilized for nucleus and nucleoli segmentation on the high-resolution image stacks (em_segmentation; 10.1101/2021.05.27.446019). 3D reconstruction and movies were created using Amira Software and Dragonfly.

Stochastic optical reconstruction microscopy

Alexa Fluor 647 conjugated trastuzumab was prepared using Alexa Fluor 647 NHS Ester (Thermo Fisher Scientific) and purified according to manufacturer recommended procedures; the final dye to antibody conjugation ratio was measured to be around 2:1 using a UV-Vis spectrometer.

SKBR3 cells were cultured in McCoy's 5A (Modified) Medium (Thermo Fisher Scientific) supplemented with 10% Fetal Bovine Serum (Thermo Fisher Scientific). For Stochastic Optical Reconstruction Microscopy (STORM) experiments, the cells were plated in Nunc Lab-Tek II Chambered Coverglass (Thermo Fisher Scientific) for 36 to 48 hours before labeling and imaging. To prepare for imaging, the cells were first serum starved overnight (~16 h); on the day of imaging, the cells were treated with 100 nM Alexa Fluor 647 conjugated trastuzumab for ~15 min, washed with pre-warmed blank medium, and placed on the microscope stage for imaging. Next, fresh STORM imaging buffer was added at 1:1,000 v/v dilution to the medium; the buffer is Phosphate Buffered Saline (PBS) supplemented with 0.5 mg/mL glucose oxidase (Sigma-Aldrich), 40 μg/mL catalase (Sigma-Aldrich), and 10% D-Glucose (Fisher Scientific); this was followed by addition of 10 mM (final concentration) cysteamine (Sigma-Aldrich). The sample was then explored at low 647 nm laser power (~100 W/cm^2; this avoids unnecessary loss of AF647 due to photobleaching) to identify regions of interest. Human Epidermal Growth Factor (EGF; Cell Signaling Technology) was diluted from a 1 mg/mL stock in PBS to 10 mg/mL and then added to the cell culture at 1:100 v/v dilution to yield a final concentration of 10 ng/mL. Image acquisition was initiated right after adding EGF, as described below. Throughout the imaging process, the cells were kept in an on-stage incubator (TokaiHit) at 37°C with 5% CO_2.

The STORM microscope setup was the same as described previously.[43] A custom single-molecule fluorescence imaging setup was built on a Nikon Ti-U microscope frame, with other essential components including an objective lens with high numerical aperture (Nikon 60x oil, TIRF, NA = 1.49), a 647 nm laser (Coherent OBIS, max output = 140 mW; for exciting and converting AF647 into a dark state), a 405 nm laser (Coherent CUBE; for converting AF647 to fluorescent on-state), and an EM-CCD (Evolve 512 Delta, Photometrics), as well as other components including dichroic mirrors and emission filters. Image acquisition was performed using microManager with an EM-CCD gain setting typically set at 300 and the frame acquisition time 8 ms (possible by selecting a small

region of interest).[83] Typical power densities for the 647 nm and the 405 nm lasers were 1–2 kW/cm^2 and 1–20 W/cm^2, respectively. Raw STORM images were processed and reconstructed using custom Matlab scripts.[84]

QUANTIFICATION AND STATISTICAL ANALYSIS

Radiology

Pre- and on-treatment FDG PET/CT studies were reviewed by an expert nuclear medicine physician with analysis performed by a body imager with 15 years of experience in oncologic imaging. Target lesions were selected by having maximum standard uptake values (SUVmax) greater than normal mediastinum average (lymph nodes) and greater than background liver SUV (liver lesions) and were recorded on the pre- and on-treatment scans at the same tumor region. Image analysis was performed using syngo.via advanced visualization software (Siemens Healthcare GmbH, Erlangen, Germany) and Horos visualization software. All lesions meeting these criteria were recorded both on FDG-PET/CT and combined with long axis measurements (e.g., liver, splenic, lung lesions) and long and short-axis measures (lymph nodes) at all time points during the care of the patient. Variability in the measurement of the long axis of each lesion was estimated to be about 20%.[85] Change in tumor burden was assessed for each phase of treatment using RECIST 1.1 criteria.[8] All SUVmax measures were normalized by subtracting the mean background SUVmax from the organ of origin (e.g., mediastinum or liver). The uncertainty in SUVmax measurements was estimated to be up to 18% based on historical test-retest reproducibility.[86]

Whole exome sequencing

Somatic mutation calling: sequence read FASTQ data files were aligned to the UCSC GRCh37/hg19 human genome build using BWA-MEM (0.7.12, GATK, Broad Institute), followed by marking duplicate reads (Mark Duplicates, GATK) and base recalibration (BQSR, GATK).[87,88] Bam files for replicate libraries were merged and somatic variants were called using MuTect2 (4.0.4.0, GATK, Broad Institute) between tumor or cfDNA and the patient's matched normal from buffy coat.[88] A panel of normal (PON) and the gnomAD (2.0.1) germline reference resource were used to filter out technical sequencing artifacts and common polymorphisms, respectively.[89] All analysis tools were run using an OHSU Galaxy instance (v17.09).[75]

Phylogenetic and Clonal Analysis: Mutect2 (GATK) and mpileup (Samtools) were used to call or detect presence of variants across all samples.[88,90] Only sequence reads with base quality greater than 20 and mapping quality greater than 30 were used for mpileup. Variants with VAF lower than 5% or depth lower than 30 reads were filtered. The R package ape was used for phylogenetic analysis.[91] A binary table of variants present across all tumor samples was generated as input. Genetic distance was estimated using the dist.gene function with the pairwise method. Minimum Evolution (ME) fit with ordinary least-squares (OLS) using the FastME function was used to reconstruct the phylogeny.

Copy Number Analysis: Copy number analysis of WES data was performed with CNVkit (v0.9.4a0) using the tumor/ctDNA aligned reads (BAM) and a pooled normal reference.[92] On- and off-target read depths from each sample were median-centered log2 normalized, followed by GC bias correction and repeat masking. Tumor copy ratios were estimated by subtracting the log2-normallized depths for each bin. Corrected copy ratio profiles were segmented using circular binary segmentation (CBS). Tumor purity estimates were then used to call each segment's absolute integer copy number.

Tumor Mutational Burden (TMB) and Neoepitope Prediction: TMB was calculated based on the number of somatic nonsynonymous mutations per megabase of the targeted regions. Neoepitopes were identified across all biopsy samples using neoepiscope v.0.5.0,[93] with HLA types predicted for all samples using OptiType v1.3.3[94] and MHC binding affinities predicted using MHCflurry v2.0.[95] All potential neoepitopes that could arise from each identified mutation were subsequently filtered based on MHC binding affinity, retaining only neoepitopes with binding affinity <500 nM for at least one patient allele.

Dual index degenerate adaptor sequencing

Error-Correction, Bait Evaluation, and Variant Analysis: The DIDA-Seq computational pipeline was implemented as previously described,[14] based on substantial modification of previous work.[96] Indexing reads containing multiplexing barcodes and degenerate unique molecular identifiers (UMI) were appended to the read header of each set of paired-end sequencing reads. Next, paired-end reads were aligned using BWA-MEM[87] and UMI families were collapsed to generate consensus sequences (requiring at least three reads and 90% agreement between reads, otherwise resulting in read omission or an "N" at a given consensus site), which were output as a FASTQ file. These FASTQ files were then realigned using BWA-MEM,[87] 3 bases from either end were replaced with "N"s, and overlapping reads were collapsed to avoid double-counting. Final BAM files were used for downstream VAF calculation (mutant allele read count divided by total read count at each site of interest) for each mutation of interest at each timepoint. All hybrid capture baits were also evaluated using unrelated patient cfDNA samples as negative controls. We sequenced each library to an average post-error correction depth of 4,000–15,000x for each site of interest and determined the VAF in these negative controls.

Quantification and Analysis: To determine the significance of a given VAF measurement, we compared the mutation-specific VAF in the patient's plasma sample data to the VAF of the same site in a set of pooled negative controls (sequenced to an average post error-correction depth of 100,000X per site, giving an average error rate of 1 in 30,000 reads) as previously described.[97] We used a Bayesian approach to test the null hypothesis that the sample VAF and negative control VAF were generated from the same distribution using R. To do this, A p value was generated for each site and mutational group of sites (Figure S2E) using the overlap

coefficient of the beta distributions between the VAF in the sample and VAF in the negative controls.[98] Any site with greater than 1% VAF in the negative controls was omitted from further evaluation. Data points having a p value greater than 0.05 were considered not statistically different from the negative controls, effectively determining our lower limit of detection given the individual or aggregated sites' sequencing depth at each time point.

Low-pass whole genome sequencing

FASTQ files were aligned to the UCSC GRCh37/hg19 human genome build using BWA-MEM,[87] and copy number alterations were called using ichorCNA with window set to "50000".[99]

Whole transcriptome sequencing

Gene Quantification: Transcript quantification followed the methods described by Tatlow and Piccolo.[100] The raw sequence reads were quality trimmed using Trim Galore, followed by pseudo-alignment and transcription quantification using Kallisto with GENCODE reference transcriptome (version 24).[101,102] Transcript level expression was aggregated to gene level abundance using the R package tximport yielding expression values for 60554 Ensembl genes.[103]

Batch Correction: Genes were filtered based on a minimum of 3 transcripts per million (TPM) in at least 3 of 48 samples, which included 29 ER+ metastatic breast cancer samples and 19 UHR samples. The filtered gene expression matrix (16,364 genes) was batch corrected by removing unwanted variation (RUV; RUVSeq).[104,105] RUV correction uses factor analysis to identify the factors of unwanted variation observed in the UHR batch control and corrects for them across all samples. RUVSeq was applied by removing 1 factor (k) using the 5% of genes with the lowest standard deviation. In addition to intra-cohort batch correct, the patient samples were batch adjusted for analyses comparing to TCGA BRCA.[100] TCGA BRCA gene expression matrix was filtered to samples with a Luminal (A or B) molecular subtype and joined with the RUV corrected patient sample gene expression. The combined matrix was log transformed, filtered to genes with a minimum of 3 log2 TPMs in at least 3 samples, and batched corrected using SVA/ComBat with TCGA samples set as the reference.[106]

Molecular Subtype Signature: The PAM50 subtype gene signature was used to classify samples into intrinsic molecular subtypes.[15] A cohort of 20 ER+ and 20 ER- samples was used as the background for classifying the patient samples' subtypes. The gene expression matrix using these 40 samples and the patient samples was mean centered and correlated (Spearman) to the pre-defined centroids from Parker et al.[15] The samples were assigned to the molecular subtype with the highest Spearman correlation to the subtype centroid.

Pathway enrichment analysis: Gene set variation analysis (GSVA) was used to estimate pathway enrichment of the MSigDB Cancer Hallmark Pathways (50 gene sets), All MSigDB Pathways (~20K gene sets), and Reactome Pathway Database (~2K gene sets).[17,18,107] GSVA used a Gaussian kernel for estimating the cumulative density function and the enrichment statistic was calculated as the difference between the maximum positive/negative random walk deviations. This analysis was applied to the RUV/ComBat adjusted log2 gene expression matrix that included both TCGA BRCA Luminal Samples and the patient samples.

Transcriptional regulator networks

Regulatory pathway and molecular interactions network: The regulatory network used to generate enrichment signatures is derived from the aggregation of publicly available molecular interactions and biological pathway databases provided by the Pathway Commons (PC) resource.[24] The aggregated data is represented in the standard Biological Pathway Exchange (BioPAX) language and provides the most complete and rich representation of the biological network models stored in PC. These complex biochemical reactions were reduced to pairwise relationships using rules to generate a Simple Interaction Format (SIF) representation of BioPAX interactions. The reduction of BioPAX interactions to the SIF allows for the representation of pairwise molecular interactions in the context of specific binary relationships. The feature space of the SIF regulatory network was restricted to primary and secondary downstream interactions for genes within Pathway Commons. The regulatory network was then reduced to edges that are associated with the binary relationship "controls-expression-of", defined as any reaction where the first protein controls a conversion or a template reaction that changes the expression of the second protein.

Network weight assignment: Weights are assigned to the protein-protein edges within the graph for each regulator-target pair within the regulatory network that is represented in the expression data set. These weights are derived from the integration of an F-test statistic to capture linear dependency and the Spearman rank-order correlation coefficient for a given regulator-target pair.

Regulon enrichment: This method leverages pathway information and gene expression data to produce regulon-based protein activity scores. Our method tests for positional shifts in experimental-evidence supported networks consisting of transcription factors and their downstream signaling pathways when projected onto a rank-sorted gene-expression signature. The gene-expression signature is derived by comparing all features to the median expression level of all samples considered within the data-set. After weights have been assigned to the regulatory network, the positive and negative edges of each regulator are rank ordered. The first component of the enrichment signature, the local delta concordance signature, is derived by capturing the concordance between the magnitude of the weight assigned to a particular edge and the ranked position of that edge. The features associated with activation, positive edges within the regulatory network, are monotonically ranked from most lowly to highly expressed in the restricted feature space, where the features that are repressed are ranked by a monotonically decreasing function. This component of the signature

considers positive and negative edges independently, which captures support for an enrichment signature even if one of the edge groups is underrepresented in the network graph. The second component of the enrichment signature, the local enrichment signature, captures positional shifts in the local gene ranked signature and integrates those shifts with the weights assigned to overlapping features for a given regulon and the expression data set. The last component of the enrichment signature considers the entire feature space and projects the rank-sorted local signature onto this global ranked feature space. We derive our global enrichment signature from this projection for each regulator we consider. We use the median of robust quantile-transformed ranked positions as the enrichment scores for both the local enrichment and global enrichment signatures. We then integrate these three individual signatures together, which allows us to capture differences between individual regulator signatures within the context of an individual patient as well as at a cohort level.

Reverse phase protein arrays

In order to scale the reported protein expression values, the RPPA data from the patient samples was merged within the TCGA breast cancer RPPA dataset, using the replicate-based normalization (RBN) method.[108] The protein expression values were then z-scored by using the median and standard deviation, and a heat-map was generated using Rank-Sum ordering of the proteins fold change. The heat map was produced using publicly available Cluster 3.0 and TreeView software.[109,110]

Pathway Analysis: All pathway predictors have been previously described.[22] Proteins used as predictors of the different pathways are listed in Table S2. To determine a pathway score, for each sample all positively associated predictors were summed minus the predictors that are negatively associated with the pathway. The total was then divided by the numbers of predictors in the pathway. To generate the pathway scores histograms, the distribution of each TCGA samples subtype was plotted and the value of the patient pre- and post-treatment sample was added to the histograms.

Intracellular signaling protein panel

Batch correction was performed using Removal of Unwanted Variation (RUV-III) using the replicate positive controls to estimate the factors associated with batch effect.[111] RUV parameters were optimized by measuring the consistency of replicate controls and careful evaluation of outliers to ensure validity of results. The GeneTrails Intracellular Signaling Protein Panel assay reports out protein expression levels from RUV-normalized data relative to tumor-type-matched reference cohorts, in this case a panel of 57 metastatic breast cancer patient samples.

Integrative analyses

Multi-omic integrated pathway analysis: CausalPath was used for integrated pathway analysis of protein, phosphoprotein, gene abundance, and transcriptional regulator activity.[25] CausalPath is a hypothesis generating tool that uses literature-grounded interactions from Pathway Commons to produce a graphical representation of causal relationships that are consistent with patterns in a multi-omic datasets.[25] This integrative approach allows for holistic evaluation of signaling networks and pathway activity across longitudinal biopsies. The CausalPath analysis used the log fold change of total and phosphoprotein (RPPA) and gene expression from Bx1 and Bx2 with the following parameters: threshold-for-data-significance = 0.3 for RNA, protein, and phosphoprotein, value-transformation = max, calculate-network-significance = true, permutations-for-significance = 10,000, color-saturation-value = 2.5, data-type-for-expressional-targets = rna and protein, show-all-genes-with-proteomic-data = true. The resulting network was pruned to include the neighborhoods encompassing MTOR, AKT, MUC1, STAT3, MYC, and E2F1 to highlight biologically interesting patterns discussed in the text. For additional depth, the difference in transcriptional regulon enrichment activity between Bx2 and Bx1 was mapped to and overlaid on the pruned CausalPath network.

Integrated Heatmap: The gene, protein, phosphoprotein abundances, and transcriptional regulon enrichment activities were integrated into a single heatmap. Each data type was independently scaled to -1 to 1 with the exception of protein/phosphoprotein, which were scaled together. Fold change of Bx2 to Bx1 was calculated for each scaled feature and represented as a heatmap grouped by pathway categories of interest.

Multiplex immunohistochemistry

Image analysis pipeline:[112] Regions of interest (ROIs) were selected from hematoxylin-stained images in ImageScope (Leica) based on histopathological assessment. ROIs were selected to capture all possible tumor area (composed of neoplastic cells and surrounding stroma), while excluding regions of adjacent normal appearing tissue, heavy RBC infiltrate, necrosis, acellular material, or areas of tissue deformation/folding which are all known to create artifactual results. The number of ROIs selected per sample was variable and sample dependent (range 2–4, average 3). ROI size was also variable (range 0.6–7.9 mm^2, average 3.7 mm^2). The tissue area and cell counts from each ROI on a given tissue were summed in order to generate global immune composition. Across the cohort, the average amount of tissue assessed per sample was 11.1 mm^2, containing an average of 68,223 nucleated cells. Digitally acquired images were registered in MatLab (MathWorks) utilizing the SURF algorithm[113] in the Computer Vision Toolbox. Nuclear segmentation and color deconvolution were performed using FIJI[81] (ImageJ, NIH). Watershed based segmentation on hematoxylin only stained tissue was used to identify single cell objects. In short, preprocessing to isolate signal and remove background was performed, then nuclear objects were identified by watershed and standard image processing (erosion, dilation, and noise removal). AEC chromogenic signal was extracted by converting images from RGB to CMYK in ImageJ[81] using the NIH plugin RGB_to_CMYK. The contrast

of AEC chromogen intensities on a 0–255 scale in the yellow channel, as compared to RGB or the built-in AEC deconvolution vector, utilizes the full range of intensity without a threshold. For single cell quantification, each channel was normalized by dividing all pixels in each image by the max intensity of that image to rescale intensity values to a range of 0–1. Next, mean intensity from each stain was quantified for every indexed nuclear object in Cell Profiler 3.1.5[114] (Broad Institute). Image cytometry hierarchical gating was performed in FCS Express Image Cytometry RUO 6.1.4 (DeNovo Software) to quantify distinct populations of cells.

Cyclic immunofluorescence

Quantification and Analysis:[115] Each image acquired was registered based on DAPI features acquired from each round of staining.[116] Cellpose, a generalist algorithm for cellular segmentation, was used to generate nuclear and cell segmentation masks with a pre-trained neural network classifier.[117] Extracted single-cell features included centroids and mean intensity of each marker from its biologically relevant segmentation mask (e.g., Ecad_Cytoplasm, Ki67_Nuclei). The last round DAPI image was used to filter out cells lost during each round of cyclic immunofluorescence staining.

For cell type determination and composition analysis, single cell mean intensities from each biopsy were batch corrected using the ComBat algorithm.[118] ComBat was used to adjust the mean and variance of fluorescence intensity on control tissue-microarrays (TMAs) that were stained with each biopsy, and the same adjustments were applied to the corresponding biopsies. Eighteen markers were selected for clustering; some markers were excluded due to tissue loss in the TMA controls. Principal component analysis was performed with scanpy to reduce dimensionality, and Umap (https://arxiv.org/abs/1802.03426) was run on the top 17 principal components to calculate a nearest neighbor graph based on the 30 nearest neighbors.[119] Leiden clustering was performed on the nearest neighbor graph to define clustering-based cell types.[120] The Leiden clustering resolution of 0.5 was selected based on appropriate clustering of technical replicates in the control TMAs.

Immune, endothelial, and stromal cells were identified by manual thresholding and gating. Endothelial cells were defined as $CD31^+$, immune cells were either $CD45^+$ or $CD68^+$ and CD31-, and stromal cells were cytokeratin-, E-cadherin-, CD31-, CD45-, and CD68-. Tumor was defined as cytokeratin+, and proliferating cells were Ki67+. Cell segmentation borders of manually defined cell types were visualized on the images using napari.[121]

To calculate distance to extracellular matrix proteins, a threshold was applied to create a pixel mask of positive staining. The distance from each nuclear centroid to the nearest mask pixel was measured. Cells were grouped into bins of 0–25 microns, 25-50 microns, and 50-75 microns from the mask, and the intensity distributions of cells (n = 2 to 32324 cells) were compared using ANOVA implemented in SciPy.[122] Significance was assigned to Bonferroni-corrected p values < 0.001.

ADDITIONAL RESOURCES

Serial Measurements of Molecular and Architectural Responses to Therapy (SMMART): https://www.ohsu.edu/knight-cancer-institute/serial-measurements-molecular-and-architectural-responses-therapy

Human Tumor Atlas Network (HTAN): https://humantumoratlas.org/

Cell Reports Medicine, Volume *3*

Supplemental information

An omic and multidimensional spatial atlas from serial biopsies of an evolving metastatic breast cancer

Brett E. Johnson, Allison L. Creason, Jayne M. Stommel, Jamie M. Keck, Swapnil Parmar, Courtney B. Betts, Aurora Blucher, Christopher Boniface, Elmar Bucher, Erik Burlingame, Todd Camp, Koei Chin, Jennifer Eng, Joseph Estabrook, Heidi S. Feiler, Michael B. Heskett, Zhi Hu, Annette Kolodzie, Ben L. Kong, Marilyne Labrie, Jinho Lee, Patrick Leyshock, Souraya Mitri, Janice Patterson, Jessica L. Riesterer, Shamilene Sivagnanam, Julia Somers, Damir Sudar, Guillaume Thibault, Benjamin R. Weeder, Christina Zheng, Xiaolin Nan, Reid F. Thompson, Laura M. Heiser, Paul T. Spellman, George Thomas, Emek Demir, Young Hwan Chang, Lisa M. Coussens, Alexander R. Guimaraes, Christopher Corless, Jeremy Goecks, Raymond Bergan, Zahi Mitri, Gordon B. Mills, and Joe W. Gray

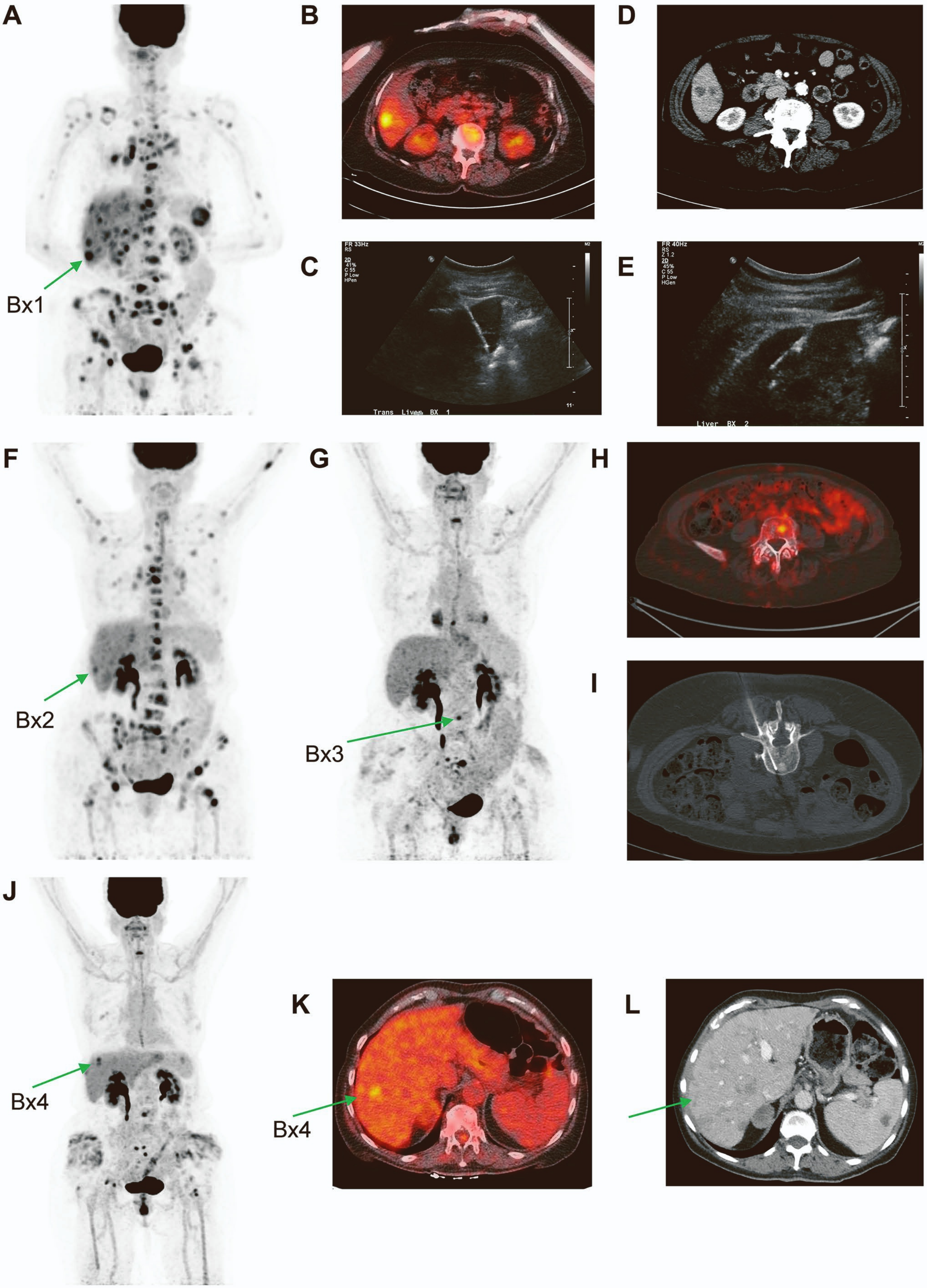

Figure S1. FDG-PET and CT Imaging, Related to Figure 2.
(A) Maximum intensity projection (MIP) FDG-PET from the beginning of Phase 1 treatment demonstrates multifocal FDG avid disease throughout the mediastinum, liver, spleen, and skeleton, including target of Bx1 (arrow). (B) Axial FDG-PET

(from same timepoint as in A) superimposed on CT showing FDG avid segment 6 liver lesion targeted by Bx1. (C) Ultrasound image from Bx1. (D) Axial contrast enhanced CT taken just before Bx2 demonstrates interval growth of a separate segment 5,6 liver lesion subsequently targeted by Bx2. (E) Ultrasound image from Bx2. (F) FDG-PET MIP image from the beginning of Phase 3 demonstrates a decrease in FDG avid lesions. Bx2 targeted lesion indicated (arrow). (G) FDG-PET taken in Phase 3 one month before Bx3 demonstrates new FDG avid lesions in the L4 vertebral body, including the Bx3 targeted lesion (arrow). (H) Axial FDG-PET (from same timepoint as in G) superimposed on attenuation correction CT showing FDG avid segment targeted by Bx3. (I) CT image from Bx3 demonstrates successful biopsy of the FDG avid, lytic lesion within the L4 vertebral body. Note the patient is prone during the biopsy. (J) FDG-PET from the end of Phase 3 demonstrates continued response in most organs but a possible new progressing liver lesion subsequently targeted by Bx4 (arrow). (K) Axial FDG-PET (from same timepoint as J) superimposed on CT showing FDG avid liver lesion targeted by Bx4 (arrow). (L) Axial contrast enhanced CT taken during month 37 demonstrates nodular, heterogeneous morphology of the liver showing signs of pseudocirrhosis (arrow).

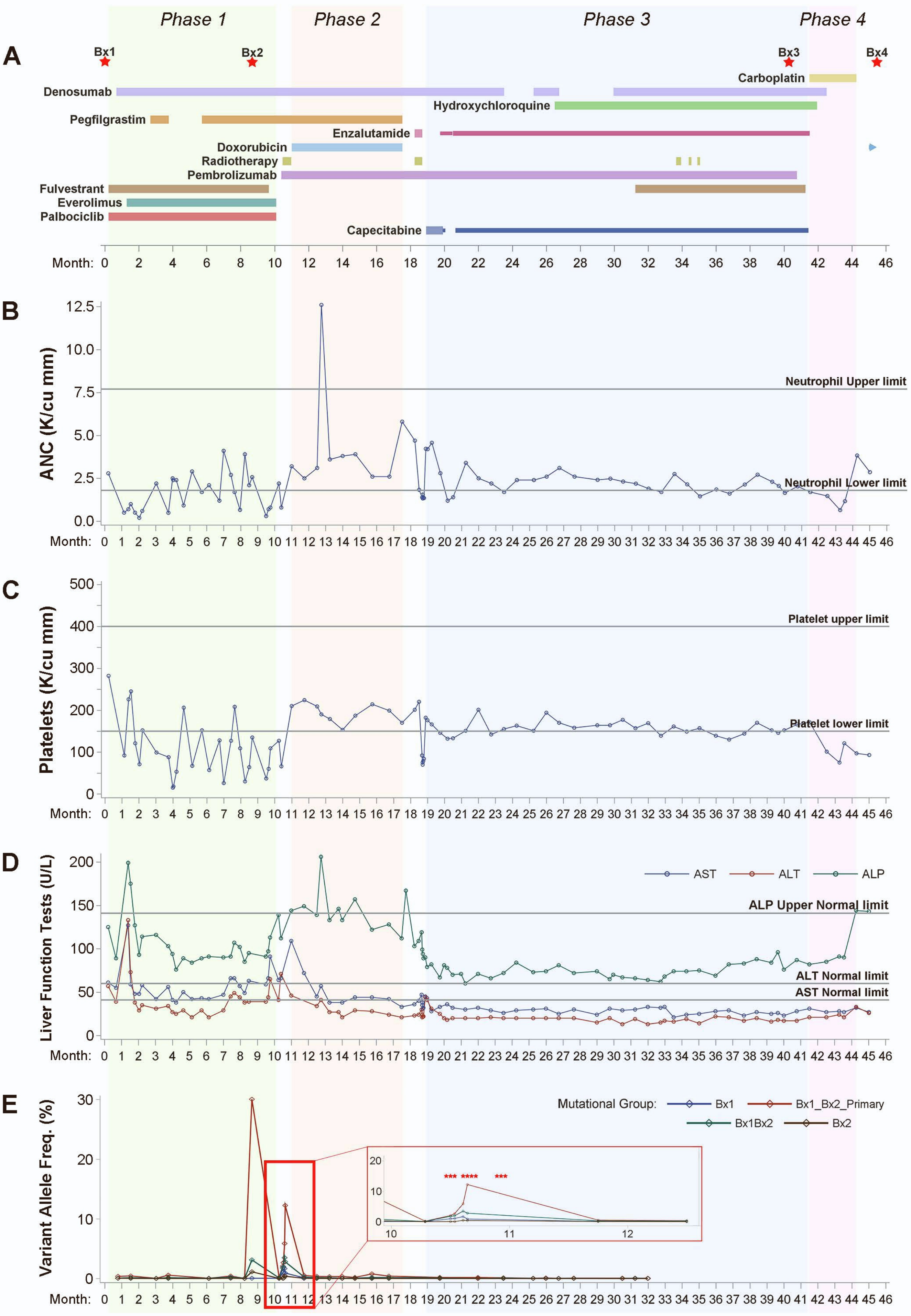

A
Phase 1
Phase 2
Phase 3
Phase 4
Bx1
Bx2
Bx3
Bx4
Carboplatin
Denosumab
Hydroxychloroquine
Pegfilgrastim
Enzalutamide
Doxorubicin
Radiotherapy
Pembrolizumab
Fulvestrant
Everolimus
Palbociclib
Capecitabine
Month:
B
ANC (K/cu mm)
Neutrophil Upper limit
Neutrophil Lower limit
C
Platelets (K/cu mm)
Platelet upper limit
Platelet lower limit
D
Liver Function Tests (U/L)
AST
ALT
ALP
ALP Upper Normal limit
ALT Normal limit
AST Normal limit
E
Variant Allele Freq. (%)
Mutational Group:
Bx1
Bx1_Bx2_Primary
Bx1Bx2
Bx2
***
****
***

Figure S2. Toxicity-Related Blood Chemistries and ctDNA Monitoring, Related to Figure 2.
(A) Treatment schedule and biopsy timing (red stars) over the course of four phases of treatment (green, orange, blue, and pink areas). Timeline sectioned into 28-day months. The duration and relative dose for each drug is indicated by the extent and width of a horizontal bar, respectively. Continuation of a drug after the end of Phase 4 is indicated by a right pointing arrow. (B) Clinically reported Absolute Neutrophil Count (ANC) in thousands per cubic millimeter (K/cu mm). (C) Clinically reported Platelet Count in thousands per cubic millimeter (K/cu mm). (D) Clinically reported results of liver function tests, including alkaline phosphatase (ALP), alanine aminotransferase (ALT), and aspartate aminotransferase (AST). (E) Longitudinal tracking of the average circulating tumor DNA (ctDNA) Variant Allele Frequency (VAF) of four different groups of mutations: Variants private to Bx1 (Bx1), private to Bx2 (Bx2), shared between the primary, Bx1, and Bx2 (Bx1_Bx2_Primary), and shared by Bx1 and Bx2 but not the primary (Bx1_Bx2). Red boxed inset shows expanded ctDNA VAF timeline during a course of palliative radiotherapy during month 10. Red asterisks indicate dates of individual radiation fractions given.

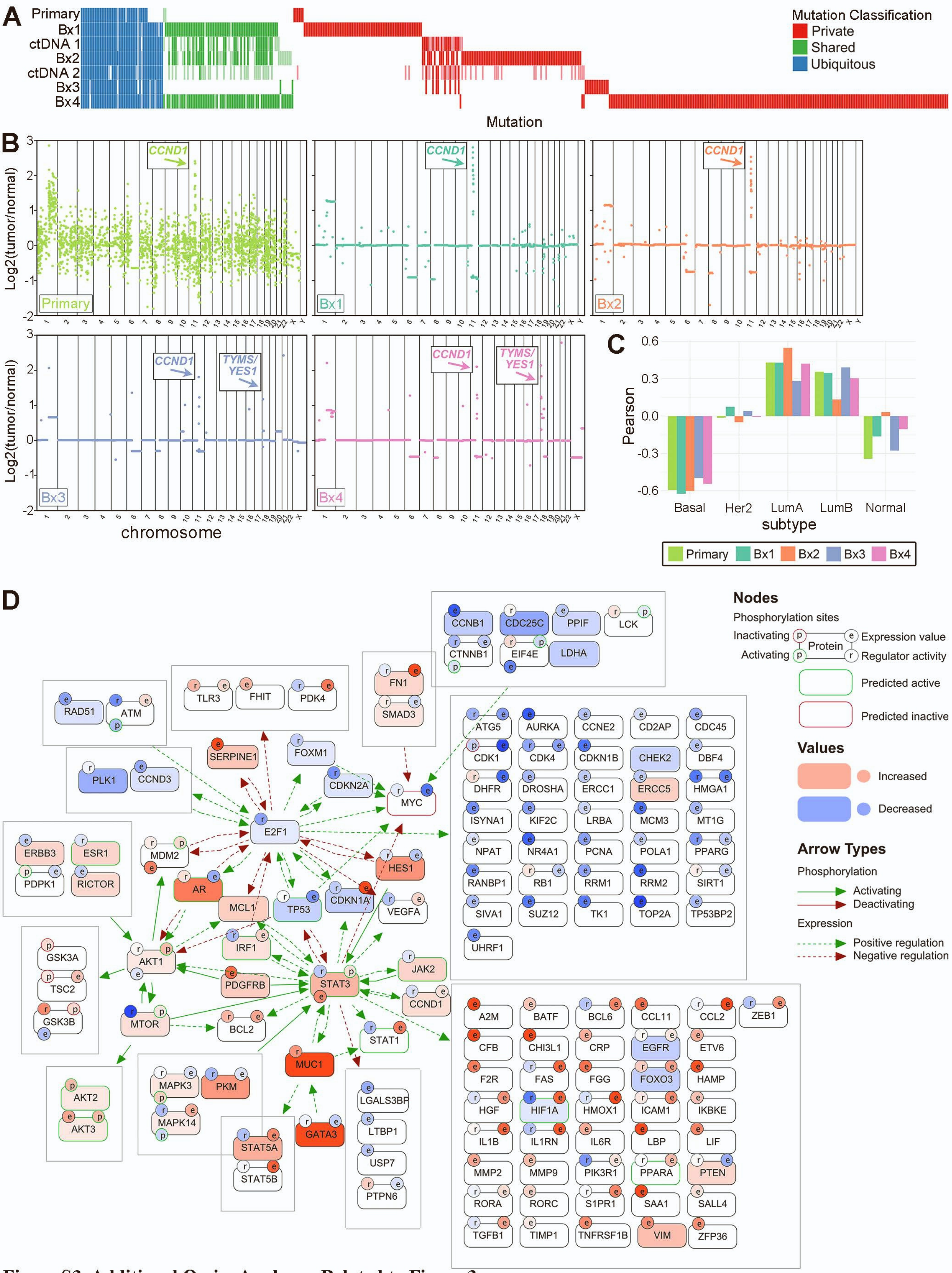

Figure S3. Additional Omics Analyses, Related to Figure 3.

(A) Non-silent SNVs and Indels identified from WES of tissue samples and classified as Ubiquitous (blue), Shared (green), or Private (red) (variants private to ctDNA timepoints not shown). Mutational status in each biopsy sample or in circulating tumor DNA from peripheral blood is indicated as independently called (colored), detected in at least 2 sequencing reads but not independently called (reduced opacity), or absent (white). (B) Scatter plots of genome-wide, segmented,

log2 copy number profiles from WES (Primary, Bx1, and Bx2) and LP-WGS (Bx3 and Bx4): Primary (yellow-green), Bx1 (green), Bx2 (orange), Bx3 (blue), and Bx4 (pink). (C) Molecular Subtype. Bar plots show the Pearson correlation of the Primary (yellow-green), Bx1 (green), Bx2 (orange), Bx3 (blue), and Bx4 (pink) samples to the PAM50 subtype centroids. (D) Integrated multi-omic pathway analysis. Pathway diagrams generated with CausalPath represent the integration of protein abundance (rectangles), phosphoprotein abundance (circle labeled ‘p’; green outline indicates activating; red outline indicates inactivating), gene expression (circle labeled ‘e’), and transcriptional regulator activity (circle labeled ‘r’), and show the change in Bx2 relative to Bx1. Networks were generated using protein/phosphoprotein abundance and gene expression, while transcriptional regulator activity was mapped on following network pruning. The red and blue fill represent higher and lower expression/activity, respectively.

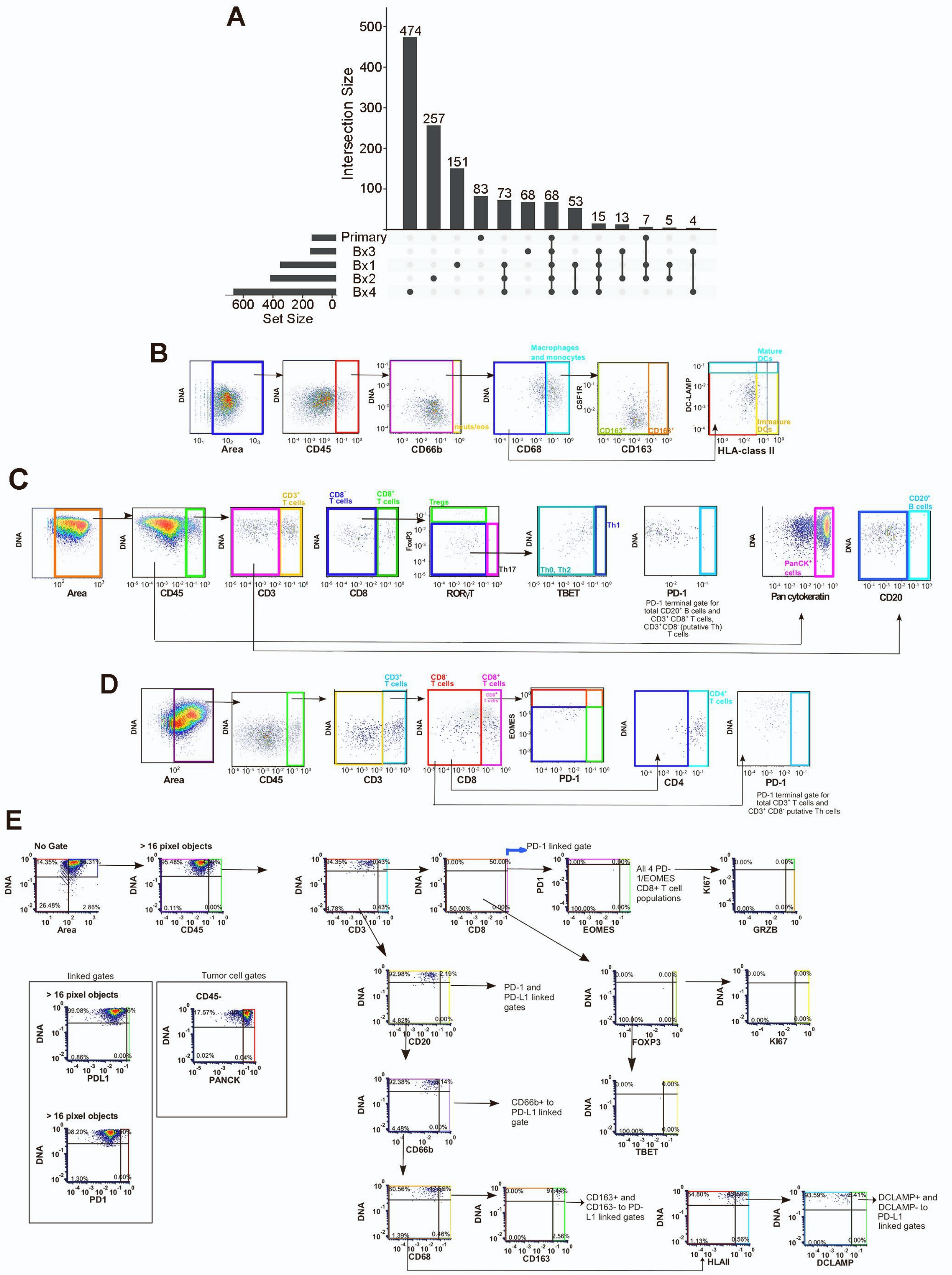

Figure S4. Predicted Neoepitopes and mIHC Gating, Related to Figure 4.

(A) Upset plot showing intersection of neoepitopes predicted to bind to at least one patient MHC allele. (B-E) Hierarchical gating via image cytometry for identification of cell types and functional states elaborated by mIHC for: (B) myeloid, (C) lymphoid, (D) functional, and (E) discovery panel of antibodies.

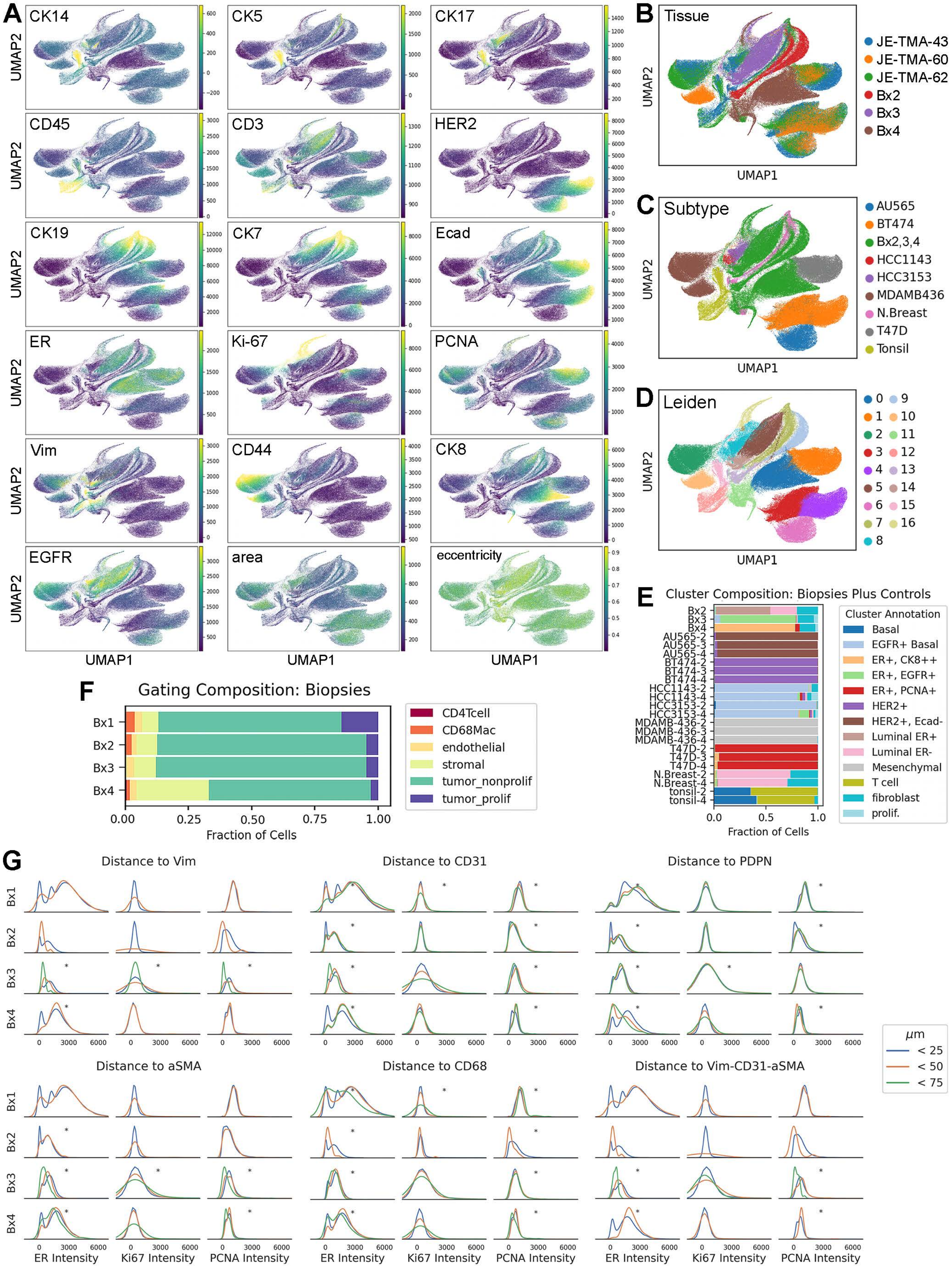

Figure S5. Additional CycIF Analyses, Related to Figure 5.
(A) Umap projection of 18 features used for clustering, colored by feature. (B) Tissues included in cluster analysis. Controls for each biopsy are as follows, Bx2: JE-TMA-43, Bx3: JE-TMA-60, Bx4: JE-TMA-62. TMAs were used for normalization, so mixing of cells from all TMAs indicates successful normalization. (C) Umap colored by cell lines and normal tissues included in clustering. (D) Umap projection colored by Leiden clustering result. (E) Composition of biopsies and

controls by annotated Leiden cluster. Number following cell line and tissue names indicates the biopsy that was paired with those controls. (F) Composition of biopsies based on manual thresholding and gating (see methods). (G) Intensity of ER, Ki67, and PCNA at 0-25, 25-50, and 50-75 μm away from various markers. Vim = vimentin, in mesenchymal cells such as fibroblasts; aSMA = Alpha Smooth Muscle Actin expressed in fibroblasts, pericytes and myoepithelial cells; PDPN = podoplanin, expressed in fibroblasts and lymphatic vessels; CD31= cluster of differentiation 31, expressed in endothelial cells; CD68 = Cluster of Differentiation 68, expressed by monocytes and macrophages; Vim-CD31-aSMA = pixels positive for any of vimentin, CD31, aSMA. Asterisks indicate significant ($p < 0.001$) difference in mean intensity between distances (ANOVA).

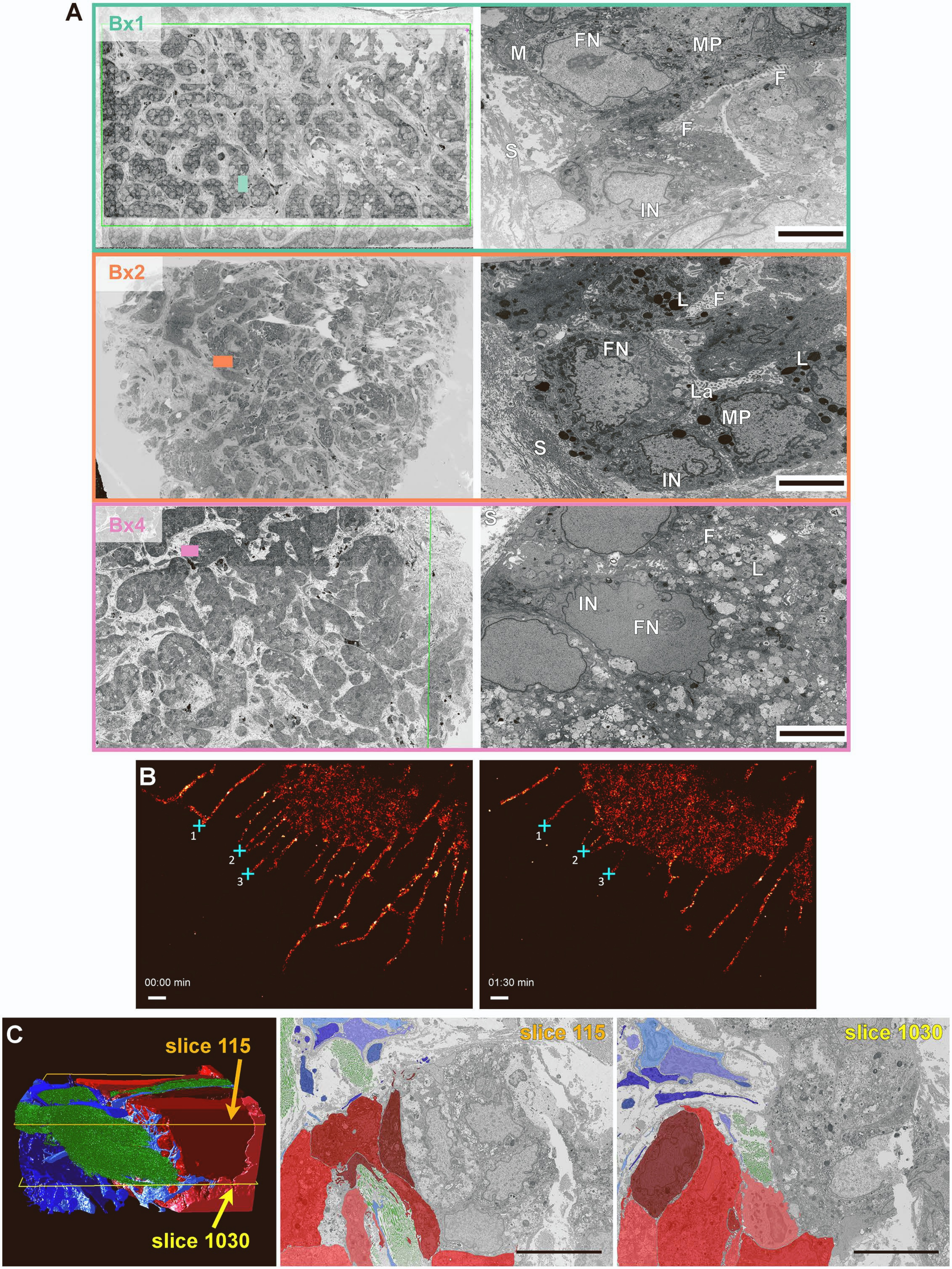

A
Bx1
M
FN
MP
F
F
S
IN
Bx2
L
F
FN
L
La
MP
S
IN
Bx4
S
F
L
IN
FN
B
1
2
3
00:00 min
01:30 min
C
slice 115
slice 1030

Figure S6. 2-D SEM, STORM, and FIB-SEM imaging, Related to Figure 6.
(A) The left column shows top-down, high-resolution blockface maps collected via 2D SEM from Bx1, Bx2, and Bx4. The boxes marked on the maps measure 25 μm in the long direction and indicate where 3D FIB-SEM was collected. The right column shows the respective first slice from the FIB-SEM volumes. Ultrastructural features of interest are marked as the following: (IN) invaginated nuclei, (FN) fenestrated nucleoli, (M) mitochondria, (L) lysosome, (S) stroma, (F) filopodia, (La) lamellipodia, (MP) macropinosomes. Scale bars, 4 μm. Bx1 shows well-defined nests of tumor cells separated by thick bands of collagen. Bx2 also shows tumor cell nests, but the tissue is denser and collagen band thickness is reduced. Bx4 shows a return to thick stromal bands and clear tumor cell nest formation, but the high-resolution view on the right shows ultrastructure different to Bx1, particularly with respect to lysosomes and macropinosomes. (B) Filopodia-like protrusions (FLPs) direct EGF-induced cell movement of SKBR3 cells. Time-lapse stochastic optical reconstruction microscopy (STORM) images of SKBR3 cells labeled with Alexa Fluor 647-conjugated Herceptin, showing abundant FLPs decorated by HER2. Left image shows initial positions of the FLPs upon addition of EGF (10 ng/mL at around -30 s). Right image shows the same field of view after 1:30 min (90 s). The cyan crosses (1, 2, and 3) mark the original locations of the tips of corresponding FLPs. Scale bars, 1 mm. (C) Segmentation of any imaging modality is limited by the 2D plane being viewed. As shown by the FIB-SEM volume, the organization, number, and area of tumor cells (red), stromal cells (blue), and collagen (green), are different depending on the depth (image slice number) of the 2D plane within the tissue sample. Slices 115 and 1030 are separated by ~9 μm. Scale bars, 10 μm.

Supplemental References

S1. Hanker, A.B., Sudhan, D.R., and Arteaga, C.L. (2020). Overcoming Endocrine Resistance in Breast Cancer. Cancer Cell 37, 496-513.

S2. Shiino, S., Kinoshita, T., Yoshida, M., Jimbo, K., Asaga, S., Takayama, S., and Tsuda, H. (2016). Prognostic Impact of Discordance in Hormone Receptor Status Between Primary and Recurrent Sites in Patients With Recurrent Breast Cancer. Clin. Breast Cancer 16, e133-140.

S3. O'Leary, B., Cutts, R.J., Liu, Y., Hrebien, S., Huang, X., Fenwick, K., Andre, F., Loibl, S., Loi, S., Garcia-Murillas, I., et al. (2018). The Genetic Landscape and Clonal Evolution of Breast Cancer Resistance to Palbociclib plus Fulvestrant in the PALOMA-3 Trial. Cancer Discov. 8, 1390-1403.

S4. Razavi, P., Chang, M.T., Xu, G., Bandlamudi, C., Ross, D.S., Vasan, N., Cai, Y., Bielski, C.M., Donoghue, M.T.A., Jonsson, P., et al. (2018). The Genomic Landscape of Endocrine-Resistant Advanced Breast Cancers. Cancer Cell 34, 427-438 e426.

S5. Nayar, U., Cohen, O., Kapstad, C., Cuoco, M.S., Waks, A.G., Wander, S.A., Painter, C., Freeman, S., Persky, N.S., Marini, L., et al. (2019). Acquired HER2 mutations in ER(+) metastatic breast cancer confer resistance to estrogen receptor-directed therapies. Nat. Genet. 51, 207-216.

S6. Giltnane, J.M., Hutchinson, K.E., Stricker, T.P., Formisano, L., Young, C.D., Estrada, M.V., Nixon, M.J., Du, L., Sanchez, V., Ericsson, P.G., et al. (2017). Genomic profiling of ER(+) breast cancers after short-term estrogen suppression reveals alterations associated with endocrine resistance. Sci. Transl. Med. 9.

S7. Levine, K.M., Priedigkeit, N., Basudan, A., Tasdemir, N., Sikora, M.J., Sokol, E.S., Hartmaier, R.J., Ding, K., Ahmad, N.Z., Watters, R.J., et al. (2019). FGFR4 overexpression and hotspot mutations in metastatic ER+ breast cancer are enriched in the lobular subtype. NPJ Breast Cancer 5, 19.

S8. Fu, X., Creighton, C.J., Biswal, N.C., Kumar, V., Shea, M., Herrera, S., Contreras, A., Gutierrez, C., Wang, T., Nanda, S., et al. (2014). Overcoming endocrine resistance due to reduced PTEN levels in estrogen receptor-positive breast cancer by co-targeting mammalian target of rapamycin, protein kinase B, or mitogen-activated protein kinase kinase. Breast Cancer Res. 16, 430.

S9. Pearson, A., Proszek, P., Pascual, J., Fribbens, C., Shamsher, M.K., Kingston, B., O'Leary, B., Herrera-Abreu, M.T., Cutts, R.J., Garcia-Murillas, I., et al. (2020). Inactivating NF1 Mutations Are Enriched in Advanced Breast Cancer and Contribute to Endocrine Therapy Resistance. Clin. Cancer Res. 26, 608-622.

S10. Lu, R., Hu, X., Zhou, J., Sun, J., Zhu, A.Z., Xu, X., Zheng, H., Gao, X., Wang, X., Jin, H., et al. (2016). COPS5 amplification and overexpression confers tamoxifen-resistance in ERalpha-positive breast cancer by degradation of NCoR. Nat Commun 7, 12044.

S11. Gupta, A., Hossain, M.M., Miller, N., Kerin, M., Callagy, G., and Gupta, S. (2016). NCOA3 coactivator is a transcriptional target of XBP1 and regulates PERK-eIF2alpha-ATF4 signalling in breast cancer. Oncogene 35, 5860-5871.

S12. Haricharan, S., Punturi, N., Singh, P., Holloway, K.R., Anurag, M., Schmelz, J., Schmidt, C., Lei, J.T., Suman, V., Hunt, K., et al. (2017). Loss of MutL Disrupts CHK2-Dependent Cell-Cycle Control through CDK4/6 to Promote Intrinsic Endocrine Therapy Resistance in Primary Breast Cancer. Cancer Discov. 7, 1168-1183.

S13. Hinohara, K., Wu, H.J., Vigneau, S., McDonald, T.O., Igarashi, K.J., Yamamoto, K.N., Madsen, T., Fassl, A., Egri, S.B., Papanastasiou, M., et al. (2018). KDM5 Histone Demethylase Activity Links Cellular Transcriptomic Heterogeneity to Therapeutic Resistance. Cancer Cell 34, 939-953 e939.

S14. Simoes, B.M., O'Brien, C.S., Eyre, R., Silva, A., Yu, L., Sarmiento-Castro, A., Alferez, D.G., Spence, K., Santiago-Gomez, A., Chemi, F., et al. (2015). Anti-estrogen Resistance in Human Breast Tumors Is Driven by JAG1-NOTCH4-Dependent Cancer Stem Cell Activity. Cell Rep. 12, 1968-1977.

S15. Alves, C.L., Elias, D., Lyng, M.B., Bak, M., and Ditzel, H.J. (2018). SNAI2 upregulation is associated with an aggressive phenotype in fulvestrant-resistant breast cancer cells and is an indicator of poor response to endocrine therapy in estrogen receptor-positive metastatic breast cancer. Breast Cancer Res. 20, 60.

S16. Bacci, M., Lorito, N., Ippolito, L., Ramazzotti, M., Luti, S., Romagnoli, S., Parri, M., Bianchini, F., Cappellesso, F., Virga, F., et al. (2019). Reprogramming of Amino Acid Transporters to Support Aspartate and Glutamate Dependency Sustains Endocrine Resistance in Breast Cancer. Cell Rep. 28, 104-118 e108.

S17. Yang, J., AlTahan, A., Jones, D.T., Buffa, F.M., Bridges, E., Interiano, R.B., Qu, C., Vogt, N., Li, J.L., Baban, D., et al. (2015). Estrogen receptor-alpha directly regulates the hypoxia-inducible factor 1 pathway associated with antiestrogen response in breast cancer. Proc. Natl. Acad. Sci. U. S. A. 112, 15172-15177.

S18. Dunbier, A.K., Ghazoui, Z., Anderson, H., Salter, J., Nerurkar, A., Osin, P., A'Hern, R., Miller, W.R., Smith, I.E., and Dowsett, M. (2013). Molecular profiling of aromatase inhibitor-treated postmenopausal breast tumors identifies immune-related correlates of resistance. Clin. Cancer Res. 19, 2775-2786.

S19. Stender, J.D., Nwachukwu, J.C., Kastrati, I., Kim, Y., Strid, T., Yakir, M., Srinivasan, S., Nowak, J., Izard, T., Rangarajan, E.S., et al. (2017). Structural and Molecular Mechanisms of Cytokine-Mediated Endocrine Resistance in Human Breast Cancer Cells. Mol. Cell 65, 1122-1135 e1125.

S20. Joffroy, C.M., Buck, M.B., Stope, M.B., Popp, S.L., Pfizenmaier, K., and Knabbe, C. (2010). Antiestrogens induce transforming growth factor beta-mediated immunosuppression in breast cancer. Cancer Res. 70, 1314-1322.
